# Chemical crosslinking and mass spectrometry to elucidate the topology of integral membrane proteins

**DOI:** 10.1371/journal.pone.0186840

**Published:** 2017-10-26

**Authors:** Mykhaylo O. Debelyy, Patrice Waridel, Manfredo Quadroni, Roger Schneiter, Andreas Conzelmann

**Affiliations:** 1 Division of Biochemistry, Department of Biology, University of Fribourg, Fribourg, Switzerland; 2 Protein Analysis Facility, Center of Integrative Genomics, Faculty of Biology and Medicine, University of Lausanne, Lausanne, Switzerland; Consejo Superior de Investigaciones Cientificas, SPAIN

## Abstract

Here we made an attempt to obtain partial structural information on the topology of multispan integral membrane proteins of yeast by isolating organellar membranes, removing peripheral membrane proteins at pH 11.5 and introducing chemical crosslinks between vicinal amino acids either using homo- or hetero-bifunctional crosslinkers. Proteins were digested with specific proteases and the products analysed by mass spectrometry. Dedicated software tools were used together with filtering steps optimized to remove false positive crosslinks. In proteins of known structure, crosslinks were found only between loops residing on the same side of the membrane. As may be expected, crosslinks were mainly found in very abundant proteins. Our approach seems to hold to promise to yield low resolution topological information for naturally very abundant or strongly overexpressed proteins with relatively little effort. Here, we report novel XL-MS-based topology data for 17 integral membrane proteins (Akr1p, Fks1p, Gas1p, Ggc1p, Gpt2p, Ifa38p, Ist2p, Lag1p, Pet9p, Pma1p, Por1p, Sct1p, Sec61p, Slc1p, Spf1p, Vph1p, Ybt1p).

## Introduction

All living cells define their boundaries by membranes containing multispan membrane proteins (MSPs), which often serve as selective pores or active pumps for nutrients and ions or as enzymes, for example, to produce membrane lipids. The elucidation of the 3D structure of such MSPs is work intensive as it typically requires the crystallization and X-ray diffraction of purified proteins maintained in solution by a belt of micellar detergent. We were motivated by recent data indicating that the active site of numerous enzymes involved in lipid biosynthesis, e.g. of the membrane bound O-acyltransferases (MBOATs), probably resides in the ER lumen, but these claims need confirmation [1–3]. Localizing the active site of such enzymes is of particular interest in that it suggests the existence of membrane transporters for substrates and products that may have to cross the membrane.

Attempting to obtain topological information by using MS/MS we initially tried to derivatize organelles with ionic, supposedly membrane-impermeant, lysine specific agents, digest membranes proteins with trypsin and detect the derivatized peptides by mass spectrometry. However, with respect to the ER, the organelle that contains the majority of enzymes of lipid biosynthesis, we failed to obtain microsomes that would be impermeant to the derivatizing agents that we used, such as for example DTSSP (3,3′-dithiobis[sulfosuccinimidyl-propionate]). In recent years, chemical cross-linking coupled with protease digestion and analysis by tandem mass spectrometry (XL-MS) has become a viable approach for determination of structural features in single proteins and oligomeric complexes [4, 5]. The latest development indicate that XL-MS could be applicable even in complex mixtures [5]. Here we report on experiments in which we used non-cleavable bifunctional crosslinkers to crosslink reactive amino acids of MSPs on both sides of the membrane; we hoped that the membrane bilayer would be thick enough to spatially separate reactive amino acids residing on opposite sides of the membrane so that crosslinks would indicate vicinity of loops on the same side of the membrane and hence reveal topological information about the MSP. This approach allowed us to obtain XL-MS based topological information for 17 yeast MSPs (Akr1p, Fks1p, Gas1p, Ggc1p, Gpt2p, Ifa38p, Ist2p, Lag1p, Pet9p, Pma1p, Por1p, Sct1p, Sec61p, Slc1p, Spf1p, Vph1p, Ybt1p).

## Materials and methods

### Yeast strains and growth conditions

Wild-type yeast strain BY4742 (*MATα his3Δ1 leu2Δ0 lys2Δ0 ura3Δ0*) was grown in YEPD medium (1% yeast extract, 2% peptone, 2% glucose) to exponential phase (OD_660nm_ of about 1) at 30°C. Strains overexpressing the TAP-tagged proteins [6] were grown in synthetic dextrose media lacking uracil (SD-URA) medium (6.7 g/l yeast nitrogen base, 2% glucose, 2.0 g amino acid dropout mix lacking uracil, pH 6.5). Expression of the TAP-tagged fusion protein was induced by growing cells in synthetic medium containing 2% galactose for 12h at 30°C.

### Isolation of a microsomal fraction

To produce spheroplasts, cells were washed two times in 1 x PBS, pH 7.4, pretreated by resuspension in DTT buffer (100 mM Tris/HCl, 10 mM DTT, pH 9.5) and incubated at 30°C for 20 min. Cells were washed in zymolyase buffer (50 mM K_2_HPO_4_, 1.4 M sorbitol, pH 7.5), resuspended in the same buffer containing the appropriate amount of zymolyase-20T (5 mg of Zymolyase-20T/g cell wet weight), and incubated at 30°C for 30 min. Spheroplasts were pelleted by centrifugation at 3’000 x g for 10 min. All subsequent steps were carried out on ice/water (0°C). Spheroplasts were washed in ice-cold homogenization buffer (50 mM K_2_HPO_4_, 1.4 M sorbitol, 2 mM EDTA, 5 mM MgCl_2_, 5 μg/ml pepstatin A, 10 μg/ml leupeptin, 2 μg/ml aprotinin, 50 μg/ml DNase, pH 7.4) and then homogenized in a Potter-Elvehjem glass homogenizer using a tight fitting pestle. After addition of 1 volume of ice-cold homogenization buffer, unbroken cells, nuclei, and large debris were removed by centrifuging at 800 x g for 5 min. The resulting supernatant was centrifuged at 3’000 x g for 5 min, then at 9’000 x g for 10 min, and finally at 20’000 x g for 30 min. The 20’000 x g microsomal pellet was resuspended in ice-cold homogenization buffer and centrifuged once again at 20’000 x g for 30 min.

For the carbonate treatment, the microsomal membrane pellet was resuspended in ice-cold SCB buffer (100 mM Na_2_CO_3_, pH 11.5) and homogenized in a pre-chilled Potter-Elvehjem glass homogenizer. After dilution by 10 volumes of ice-cold SCB buffer, the membranes were centrifuged at 20’000 x g for 60 min. The membrane pellet was resuspended in SCB buffer using the glass Potter as described above, 10 volumes of ice-cold SCB buffer were added and membranes were centrifuged at 20’000 x g for 60 min. Carbonate treated membranes were then resuspended in ice-cold XLB buffer (20 mM HEPES, 300 mM NaCl, pH 7.5) using a glass homogenizer. After addition of 10 volumes of ice-cold XLB buffer, membranes were pelleted by centrifugation at 20’000 x g for 60 min. The final membrane pellet was resuspended in ice-cold XLB buffer (10 ml of buffer/g pellet) and protein concentration was determined using the Pierce BCA kit (Thermo Fischer Scientific).

### Crosslinking reactions

Tubes with BS3-d0 and BS3-d4 crosslinkers (bis[sulfosuccinimidyl] suberate; d4 contains 4 deuteriums instead of the hydrogens present in d0) were equilibrated at ambient temperature before opening to prevent condensation. Crosslinkers were weighed immediately before use and added as dry powder (to 10 mM) to membrane suspensions (3 mg/ml) in XLB buffer. The samples were kept in an ice water bath with shaking for 1 hour. Unreacted BS3-d0/BS3-d4 was quenched by adding 1 M Tris-HCl buffer pH 7.4 (1/10 of reaction volume), and samples were mixed on an overhead rotary shaker during 15 min at room temperature. Samples were diluted by the addition of 10 volumes of ice-cold XLB buffer and membranes were sedimented at 20’000 x g for 60 min. Then, ice-cold XLB buffer was added to pellets (1 ml of buffer/g of wet pellet) and the pellet was homogenized in a pre-chilled Potter-Elvehjem glass homogenizer. The protein concentration was determined using the Pierce BCA kit and aliquots of 40 μg of crosslinked membrane proteins were placed into Eppendorf tubes, which were snap frozen in liquid nitrogen and then stored at -80°C.

Crosslinking with EDC (1-ethyl-3-(3-dimethylaminopropyl) carbodiimide hydrochloride was performed essentially as described for BS3, but using a higher concentration of EDC (25 mM final concentration.) and the addition of Sulfo-NHS (*N*-hydroxysulfosuccinimide, 12.5 mM final concentration) to enhance the efficiency of EDC crosslinking.

### Protein digestion

Crosslinked membrane samples were centrifuged in Eppendorf tubes at 16’000 x g for 60 min (4°C). The supernatant was removed and the membrane pellet was resuspended in urea buffer (8.0 M urea, 100 mM Tris/HCl, pH 7.5) by vortexing at room temperature during 10 min. Disulfide bridges were then reduced by addition of DTT (5 mM) and samples were incubated at 37°C for 30 min. Denatured proteins were alkylated by adding iodoacetamide or sodium iodoacetate (20 mM), depending on the experiment (see S1 Table) and incubating at room temperature in the dark for 20 min. Excess sodium iodoacetate was quenched by addition of DTT (20 mM) and samples were incubated at room temperature for 10 min. Then, proteins were digested by the addition of the endoproteinase LysC to a final concentration of 0.002 μg/μg protein. Samples were incubated in the dark at 37°C on a shaker (150 rpm) for 4 h. After addition of ammonium bicarbonate buffer (50 mM NH_4_HCO_3_, pH 7.5) to dilute urea to 2.0 M, trypsin was added at a final concentration of 0.05 μg/μg protein. Samples were incubated at 37°C with shaking (150 rpm) during 15 h. Peptides were then dried in a SpeedVac rotary evaporator at room temperature, before being resuspended in a minimal volume (typically 50 μl) of 0.1% trifluoroacetic acid in HPLC-grade water. Peptides were desalted by using C18 Millipore ZipTip Pipette Tips. Samples were dried in a SpeedVac at room temperature and resuspended in a minimal volume (typically 20 μl) of 0.1% trifluoroacetic acid in HPLC-grade water. They were then frozen in liquid nitrogen in 1.5 ml Eppendorf tubes and kept at -80°C for MS/MS analysis. Some samples (see S1 Table) were further fractionated on a SCX micro-column [7].

### LC-MS/MS analyses

Samples were injected on a Dionex RSLC 3000 nanoHPLC system (Dionex, Sunnyvale, CA, USA) interfaced via an Easy Spray source to a high resolution mass spectrometer QExactive Plus (Thermo Fisher, Bremen, Germany). Peptides were first trapped onto a microcolumn Acclaim PepMap100 C18 (20 mm x 100 μm ID, 5 μm, Dionex) before separation on an Easy Spray C18 PepMap nanocolumn (25 or 50cm x 75μm ID, 2μm, 100Å, Dionex) at a flow rate of 0.30 (25 cm column) or 0.25 μl/min (50 cm column). A gradient from 4 to 76% acetonitrile in 0.1% formic acid was used for peptide separation (total time: 140min). Full MS survey scans were performed at 70,000 resolution, and the 10 most intense multiply charged precursor ions in each window were selected for higher energy collision-induced dissociation (HCD, normalized collision energy NCE = 27%) and analysis in the Orbitrap at 17’500 resolution. The window for precursor isolation was of 1.5 m/z units, and selected masses were then excluded for 60s from further analysis.

### Data analysis

MS/MS spectra were extracted from raw files and converted to mgf format (Mascot generic file) using ProteoWizard 3.0 (http://proteowizard.sourceforge.net). For identification of (normal) linear peptides, spectra were analyzed using Mascot (Matrix Science, London, UK; version 2.5.1), set up to search the UniProt (http://www.uniprot.org) database restricted to *Saccharomyces cerevisiae* taxonomy (UniProt, December 2015 version: 7,903 sequences) and a custom database containing usual contaminants (digestion enzymes, keratins, etc.). A fragment ion mass tolerance of 0.020 Da and a parent ion tolerance of 10 ppm were used assuming the digestion enzyme trypsin with two or three missed cleavages. Carboxymethylation or carbamidomethylation of cysteine was specified in Mascot as a fixed modification. Oxidation of methionine and acetylation of the protein N-terminus were specified as variable modifications. Additional variable monosubstitution modifications of lysine and protein N-termini by suberate (light or D4 heavy version) were also considered in samples treated with BS3-d0 and BS3-d4 crosslinkers.

Scaffold 4 (Proteome Software Inc., Portland, OR) was used to validate MS/MS based peptide and protein identifications. Peptide identifications were accepted if they could be established at greater than 90.0% probability by the Scaffold Local FDR algorithm. Protein identifications were accepted if they could be established at greater than 95.0% probability and contained at least 2 identified peptides. Protein probabilities were assigned by the Protein Prophet algorithm [8]. Proteins that contained similar peptides and could not be differentiated based on MS/MS analysis alone were grouped to satisfy the principles of parsimony. Proteins sharing peptides with significant sequence homologies were grouped into clusters.

### Crosslinking analysis

To identify crosslinked peptides StavroX version 3.5.1 [9] and pLink version 1.23 [10] were used for MS/MS data interpretation with the following settings. Cleavage enzyme and specificity: Trypsin (specific cleavage); Protease cleavage sites: K, R; missed cleavages: K: 1, R: 3; sites blocked by phosphorylation (P) are not cleavable; minimum peptide length: StavroX: 7 amino acids; pLink: 4 amino acids. Variable modifications: methionine oxidation (maximum: 2 per XL). Static modifications: cysteine reduction and carboxymethylation or cysteine reduction and carbamidomethylation (depending on the experiment, see S1 Table). Crosslinker-I: bis(sulfosuccinimidyl)suberate (BS3), used as a 1:1 mixture of BS3-H4/BS3-D4; crosslinked amino acids: (1) K, N-terminus; (2) K, S, T, Y, N-terminus; maximal retention time (RT) difference: 20 seconds; Crosslinker-II: 1-ethyl-3-(3-dimethylaminopropyl)carbodiimide hydrochloride (EDC); crosslinked amino acids: (1) K, N-terminus; (2) D, E, C-terminus; Both crosslinkers: Precursor precision: deviation < 5.0 ppm; fragment ion precision: deviation < 0.03 Da; lower mass limit: 200 Da; upper mass limit: 6000 Da; signal to noise (S/N) ratio: 1.0; ion types: a, b, and y, unless indicated otherwise; neutral loss: only of identified fragments (H_2_O and NH_3_). In StavroX, a score cutoff of 50 was selected to control the FDR (False Discovery Rate) to within 5%.

## Results

### Experimental strategy

**Fig 1:**
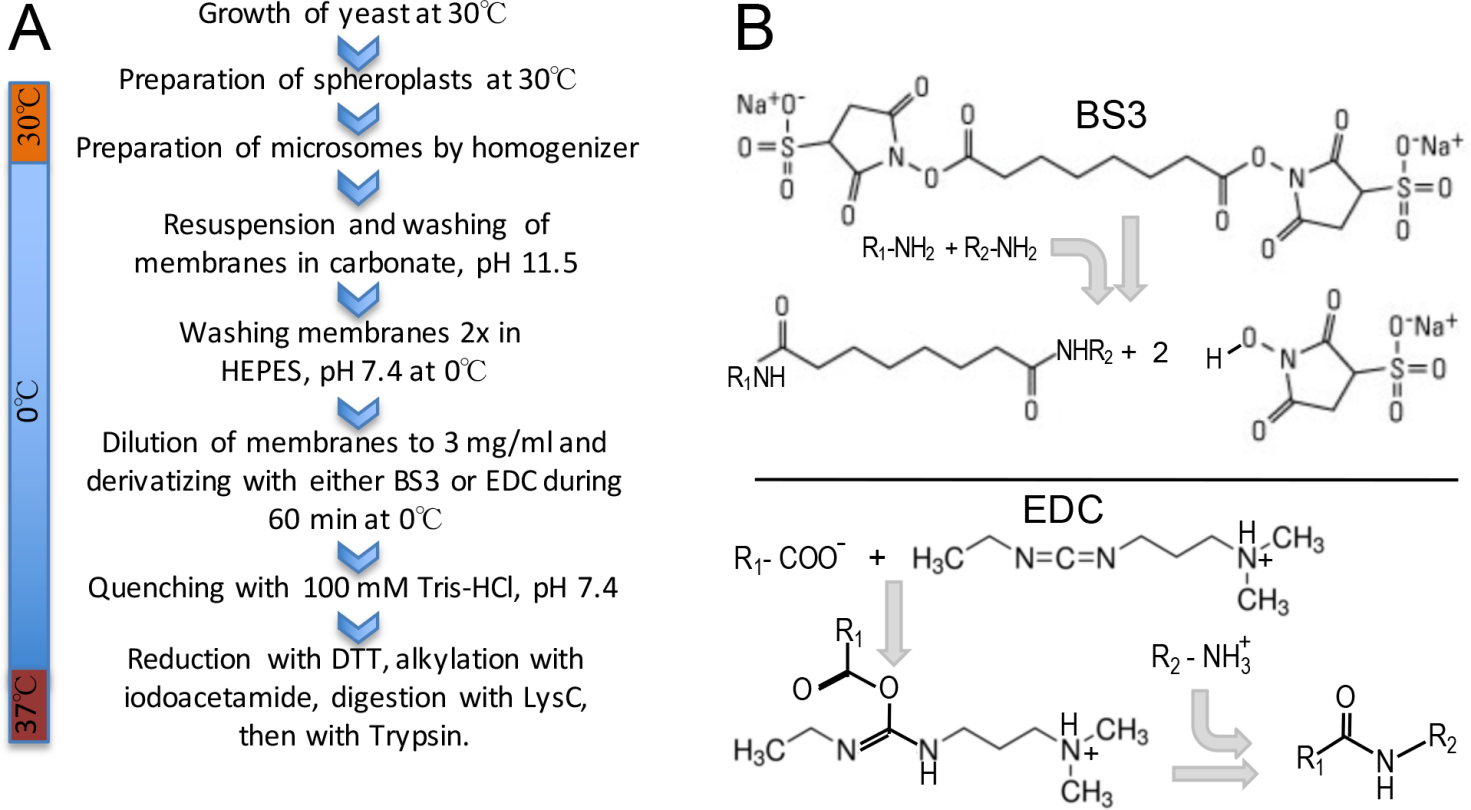
Experimental workflow and chemical structure and properties of the crosslinking agents employed. **A,** schematic representation of the experimental workflow used for growing cells, preparing microsomes, carbonate wash of membranes, chemical crosslinking of proteins and preparation of peptides for MS analysis. **B,** chemical structure and reactivity of the crosslinkers employed BS3 (bis[sulfosuccinimidyl] suberate) and EDC (1-ethyl-3-(3-dimethylaminopropyl) carbodiimide hydrochloride.

The work flow of experiments is depicted in Fig 1A. Microsomes were obtained from spheroplasts as described in Materials and Methods. We estimated that a carbonate wash (100 mM Na_2_CO_3_, pH 11.5 at 0°C (ice water bath) to remove peripheral membrane proteins and ribosomes would be beneficial as it would improve the accessibility of the integral membrane proteins to crosslinkers and reduce the complexity of the sample. Obviously, this incurred the risk that proteins may be denatured by the high pH. We used two distinct crosslinking agents, shown in Fig 1B. BS3 reacts with primary amines and hydroxyls in certain contexts [11] whereas EDC activates carboxyl groups for spontaneous reaction with primary amines [12, 13]. BS3 introduces a spacer of 11.4 Å, whereas EDC crosslinks directly the primary amine of Lys or protein N-termini to the carboxyl group of Glu, Asp or the C-terminus without adding any additional atom and causing a net loss of one water molecule. Data reported here are integrating the crosslinks (XLs) of 15 different experiments, 9 of which were done with a 1:1 mixture of BS3-d0 and BS3-d4 (BS3-d4 containing 4 deuterium instead of hydrogen atoms), one experiment was performed with only BS3-d0, and 5 experiments used EDC.

For 13 of the 15 crosslinking experiments, microsomes were prepared from wild-type yeast cells cultivated in rich media (YEPD) to the exponential phase. For the remaining 2 experiments, strains overexpressing one of the following TAP-tagged MSPs were used: Aur1p, Elo2p, Elo3p, Gpt2p, Ipt1p, Sct1p, Sec61p, Sur2p, Ydc1p, Ypc1p, and Slc1p. These cells were grown in galactose containing synthetic media lacking uracil to induce the overexpression of one of these 11 proteins from a *GAL1* promoter [6]. Equal number of cells from these 11 strains were then pooled before microsomes were prepared. Microsomes were treated with carbonate and proteins were crosslinked by the addition of BS3-d0/BS3-d4 (10 mM) or EDC (25 mM).

After crosslinking for 1 h at 0°C and quenching of excess crosslinker, protein samples were sequentially treated with LysC, a protease cleaving after Lys only, then with trypsin, cleaving after Lys and Arg as described [14] and desalted peptides were analyzed by high-resolution mass spectrometry on a QExactive Plus instrument.

### Software used to identify intramolecular crosslinks

In comparison to tryptic peptides generated from samples that are not derivatized or derivatized with mono-functional reagents, the theoretical number of possible combinations of two peptides linked after chemical crosslinking is enormous. We however assumed that XLs would occur most frequently between amino acids belonging to a single protein (intramolecularly) or between subunits of a protein complex. We mainly used StavroX 3.5.1 and pLink 1.23 to analyze the MS data [9, 10]. These software tools can be programmed to calculate the masses of all tryptic peptides of a given protein with a user-defined number of missed cleavage sites and modifications and then determine the masses of all possible XLs between any two peptides after adding the mass of the spacer. The masses and fragmentations of the theoretical XLs of a given protein are then compared to the masses and fragmentations obtained in the experiments and present in the Mascot generic files (mgf). For MSPs of interest the XLs identified in the 15 experiments are listed in supplemental tables (S10 Table for StavroX, and S14 Table for pLink). The.raw files, Mascot.dat and.mgf files as well as Proteome Software Scaffold files (.sf3 files) showing non-crosslinked peptides are available at the ProteomeXchange server (accession PXD006707).

Based on the observation that, disuccinimidyl suberate, a non-sulfated version of BS3 also reacts with Ser, Thr and Tyr, we specified the pLink and StavroX searches for BS3 experiments to detect not only XLs between two Lys residues but also a primary amine and Ser, Thr and Tyr residues [11]. For EDC experiments only Lys-Asp or Lys-Glu XLs were permitted. Note that the EDC strategy utilized here involved addition of N-hydroxysulfosuccinimide (Sulfo-NHS) so that carboxyls of Asp and Glu could be transformed into acyl-sulfosuccinimides, which then could react with lysines. This theoretically also opens the possibility that carboxyls would be crosslinked to residues such as Ser, Thr and Tyr, but we have not investigated whether this had been occurring.

### Validation

The main challenge was to find criteria that would reliably discriminate between true and false XLs. StavroX was developed to analyze the products generated by crosslinkers added to purified proteins or protein complexes whereas pLink was designed to find XLs in complex samples. To define parameters that would allow to filter false positive results, we used StavroX to analyze our mgf files for XLs within proteins that were not present in our samples, such as bovine serum albumin (BSA, gi|767930), the human homologue of yeast Drs2p (gi|115527934), or the human homologue of Neo1p (gi|115527934) (S11 Table). This analysis allowed us to define filtering constraints that allowed the elimination of such false positive XLs. These include that the shorter of the cross-linked peptides should be at least 7 amino acids long. This ≥7 amino acids rule also removed all XLs linking two different yeast MSPs that were known to reside in different cellular locations or in different protein complexes and therefore were expected not to be crosslinkable. Thus, when the ≥7 amino acids rule was applied, there were no XLs between the mitochondrial inner membrane acyltransferase Taz1p and proteins of the secretory pathway such as Cho2p, Lcb1p, Ipt1p, Neo1p, Dnf1p, nor where there any XLs found between Gpi8p, the catalytic subunit of the GPI transamidase complex and Lcb1p, being part of the Orm complex or between Gpi8p and Gpt2p, an ER protein not residing in a multisubunit complex (S12 Table). The data establishing the ≥7 amino acids rule are graphically shown in S2 Table. However, there was a major problem in as much as numerous MS spectra were claimed as a XL by two different proteins, whereby in EDC-XLs this “promiscuity” could be reduced to about 6% of XLs, when the mass deviation cut off was reduced to < 2.5 ppm (S3 Table). The same restriction did not reduce promiscuity in BS3-XLs (not shown). Also, StavroX was finally abandoned because of 5260 BS3-XLs identified in 95 yeast MSPs, only 14 had significant scores for both isotopic forms of BS3, BS3-d0 and BS3-d4 (not shown). Finally, even when the ≥7 amino acids rule was applied, StavroX discovered very numerous BS3-XLs in the abundant yeast plasma membrane proton ATPase Pma1p not only when searching the BS3-mgf files, but also when searching the EDC-mgf files and inversely, it found very numerous EDC-XLs for Pma1p also in the BS3-mgf files (S5 Table). As we could not define parameters that would differentiate between XLs found in the corresponding and the non-corresponding mgf file, we considered StavroX inappropriate for the analysis of our complex sample. Nevertheless, some XLs in very abundant proteins gave reasonably fragmented ions and StavroX results are available in supplemental section (S1 Fig, and S10–S13 Tables) to allow getting a general impression of the quality of analysis of XLs by StavroX.

**Fig 2:**
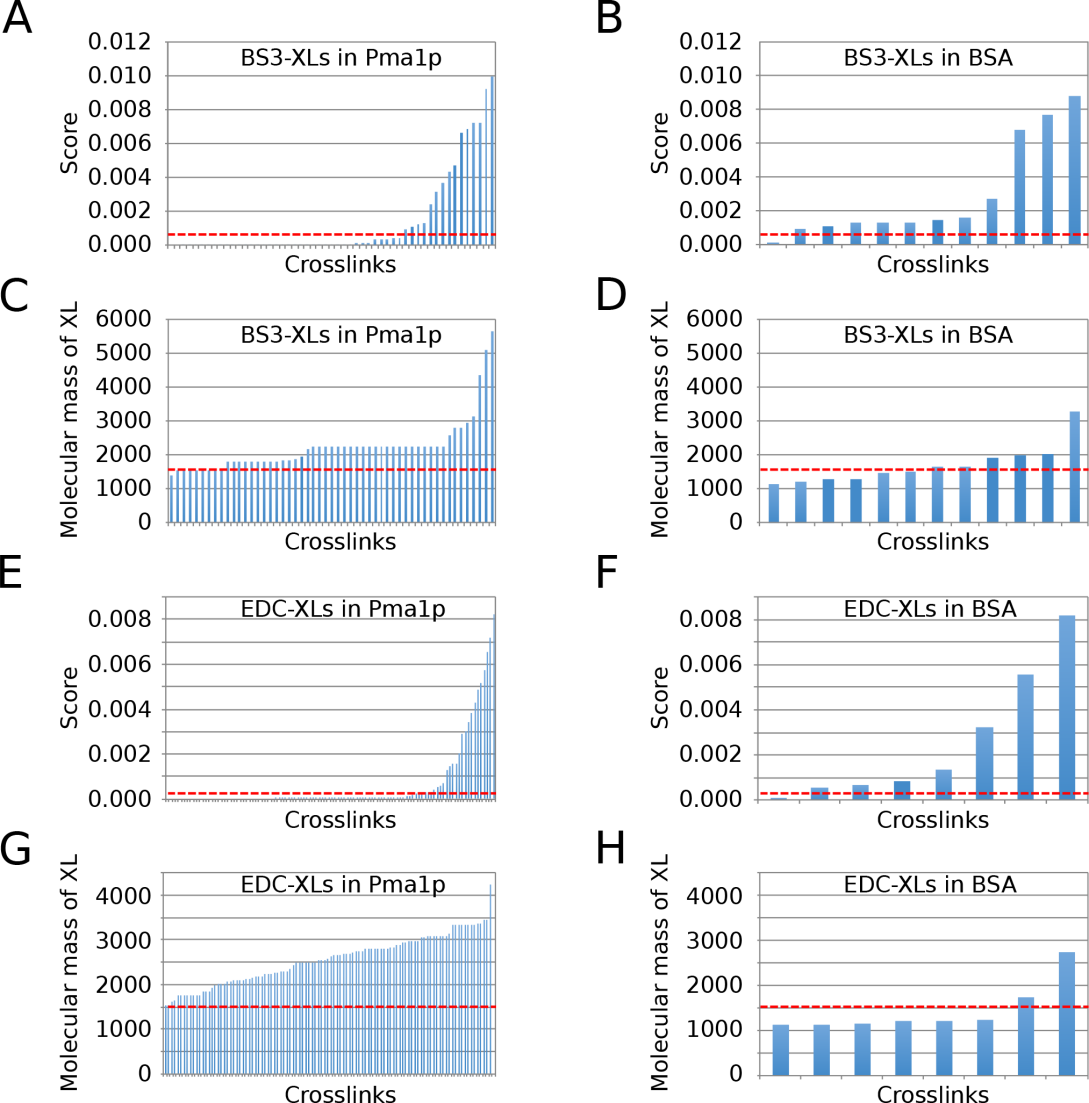
Molecular mass and scores of XLs found by pLink for Pma1p and BSA. Distributions of scores and calculated molecular masses of XLs generated by pLink by scanning through all mgf files from BS3 experiments (**A-D**) or EDC experiments (**E-H**) for Pma1p and BSA, a protein that is not present in the sample, are plotted as calculated in S7 Table. The cut off lines used to filter all pLink XLs (stippled red lines) indicate a minimal molecular mass of XL of 1’500 Da and scores of ≤ 7.5 x 10^−4^ for BS3-XLs, or ≤ 4 x 10^−4^ for EDC-XLs.

As for pLink, the software found 8 BS3-XLs for Pma1p in the corresponding BS3-mgf files and none in the EDC-mgf files (S6 and S14 Tables). In the inverse case, pLink found 59 EDC-XLs in the corresponding file (S6 and S14 Tables). pLink also found many BS3- and EDC-XLs in proteins that were not present in our sample (e.g., BSA, human Drs2 and human Neo1 orthologs) (S15 Table). Comparison of the scores and theoretical masses of the XLs in MSPs and those found in BSA, an absent protein, allowed to propose a filtering that removed almost all false positive XLs (Fig 2, and S7 Table).

This filtering removed XLs having a theoretical mass of ≥ 1500 Da and scores ≤ 7.5 x 10^−4^ for BS3-XLs, or ≤ 4 x 10^−4^ for EDC-XLs. These limits removed all but one XL found for BSA, all XLs found for two absent proteins, human Drs2p and human Neo1p orthologs (S15 Table), and also removed all EDC-XLs pLink found in the inappropriate BS3-mgf files (S6 Table). Thus, this filtering removed 71of 72 false crosslinks. XLs were identified more efficiently by pLink when searching for XLs with the BS3-d4/BS3-d0 mode than with the BS3-d4 mode only (S8 Table).

### Frequency of reliable crosslinks depends on protein abundance

**Fig 3:**
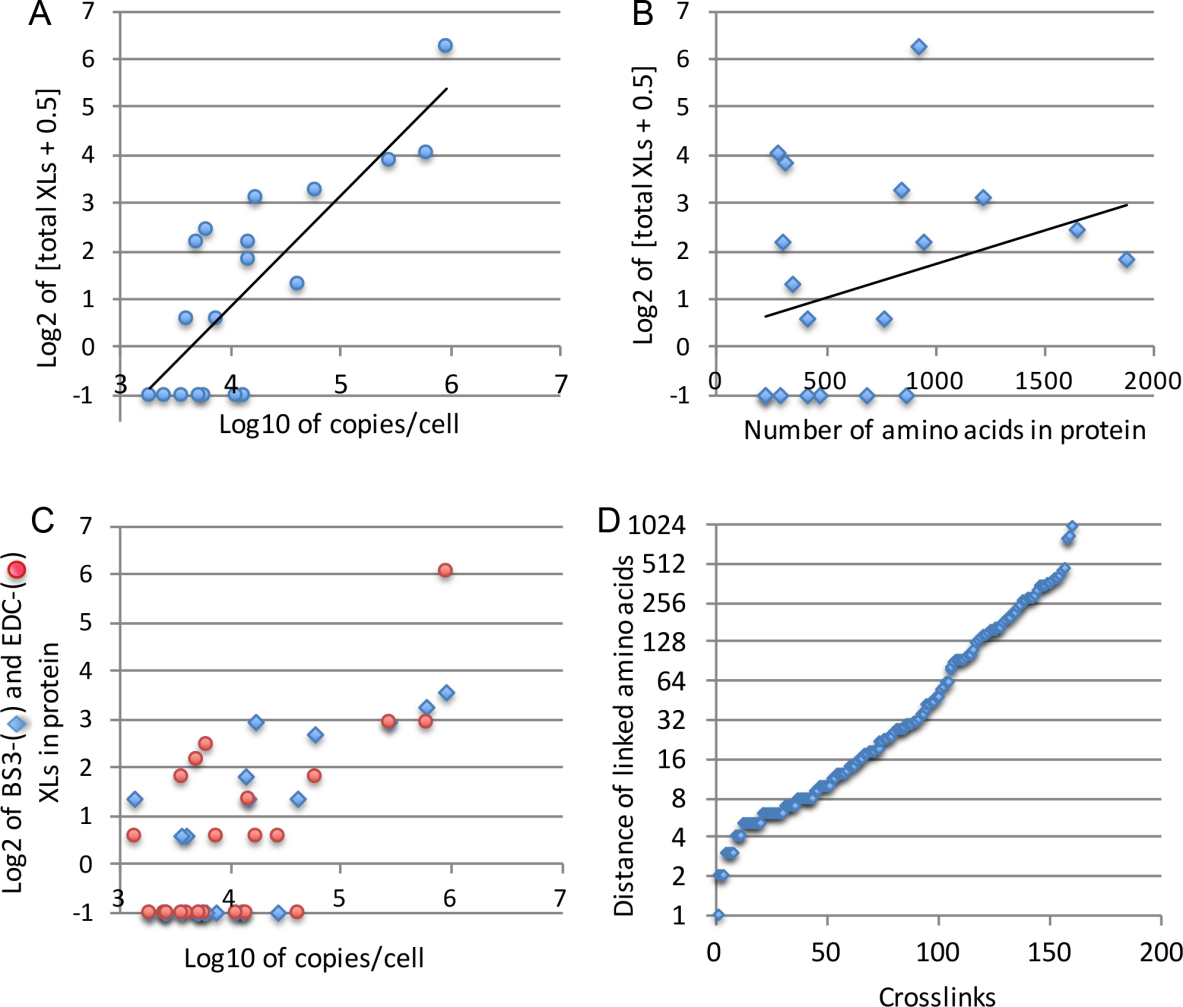
Frequency of XLs found by pLink for a given protein is correlated with its copy number. The number of XLs for 18 arbitrary chosen MSPs listed in S4 Table is plotted as a function of the copy number of the respective MSP (**A**) or its amino acid length (**B**). In panel **C**, the number of BS3- and EDC-generated XLs for these 18 proteins are plotted separately, as a function of copy number. In panel **D**, the distance in the primary sequence between crosslinked amino acids in 160 XLs found in 27 proteins of S14 Table are plotted.

The filtering of pLink data removed about 30% of XLs, so that we finally ended up with merely 160 XLs in a total of 27 different proteins analyzed (S14 Table), quite in contrast with the thousands of XLs found by StavroX. When only considering the 18 arbitrarily chosen MSPs, which had not been overexpressed (S4 Table), 86% of XLs of a total of 143 XLs were present in the 5 most abundant proteins (Pma1p, Por1p, Vph1p, Pet9p and Spf1p), and Pma1p alone made up for more than half of the XLs (76 XLs). Indeed, Pma1p is one of the most abundant MSP and reported to be present at 1’260’000 and 113’952 copies per cell by two independent studies [6, 7]. Indeed, the number of XLs found per protein after filtering was strongly correlated with the abundance of the protein as reported in the literature (R = 0.913, Fig 3A) [6, 7], but only weakly with the size of the protein (R = 0.124, Fig 3B). This was true for both, BS3- and EDC-generated XLs (Fig 3C). The strong correlation between copy number/cell and number of XLs/protein (Fig 3) in itself is in favor of the XLs that remain after filtering being true XLs.

### Topological information to be gathered from crosslinks

Many XLs linked two amino acids that were close to each other in the primary sequence, as expected, but others linked amino acids that are positioned far from each other (Fig 3D, S9 Table). XLs linking amino acids that are close to each other and therefore reside on the same hydrophilic loop do not yield topological information on secretory MSPs. The links between more distant amino acids, however, often yield topological information, if one assumes that linked amino acids have to be on the same site of the membrane. This assumption is based on what we observed in MSPs of known structure, but since the number of such structures and XLs is very limited, this assumption needs to be tested through further experiments (see Discussion). TOPCONS and a HHPRED model propose for Pma1p 10 transmembrane domains (TMDs), 4 cytosolic loops and cytosolic N- and C-termini: Only 12 XLs out of 76 were topologically relevant and they linked 4 out of the proposed 6 cytosolic parts amongst each other. No links were observed amongst the 4 loops predicted to be luminal but also, these luminal loops contain a grand total of 43 amino acids, whereas cytosolic parts contain 612 amino acids, making cytosolic XLs much more likely than luminal ones.

**Fig 4:**
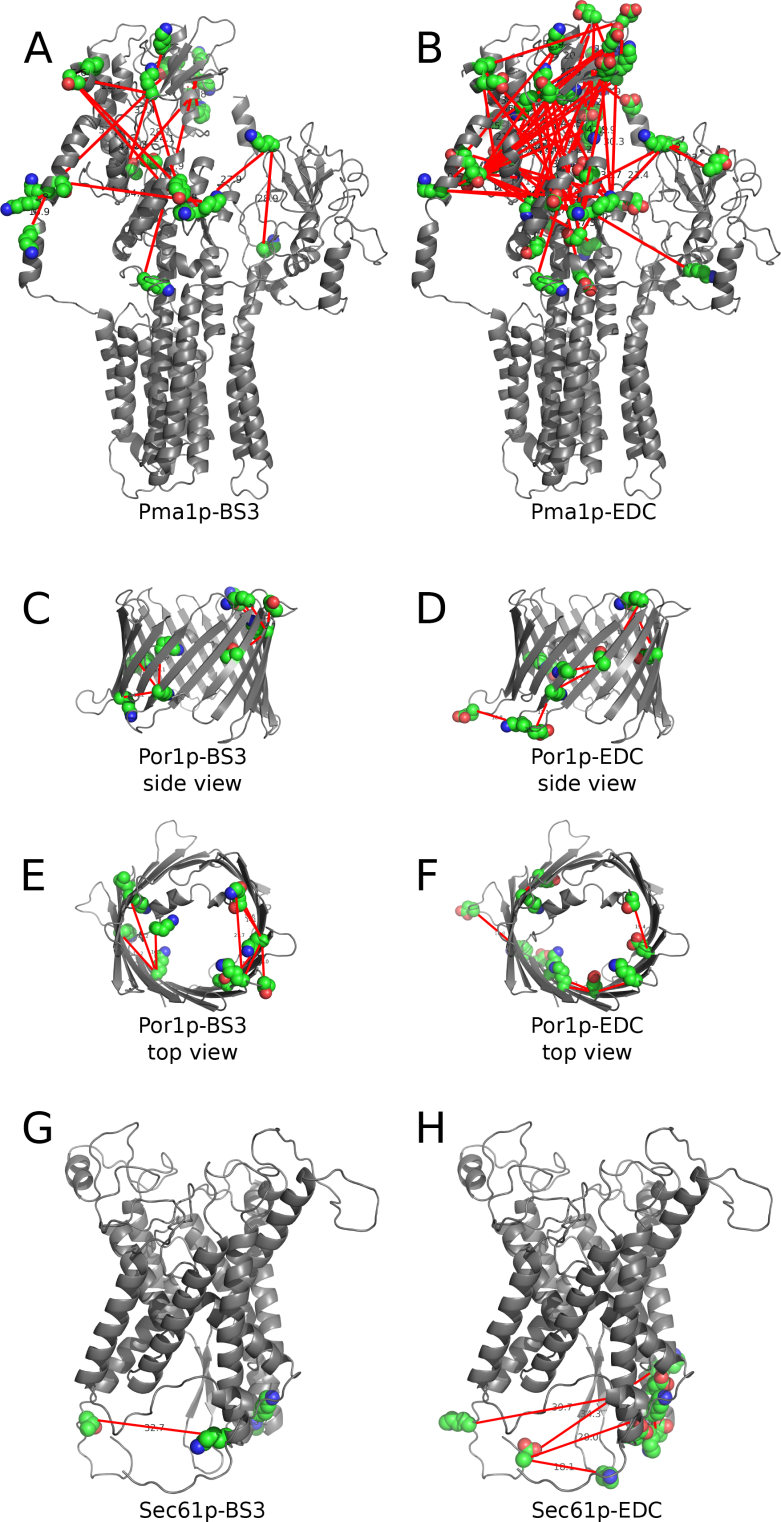
BS3 and EDC crosslinks mapped onto the structural models of Pma1p, Por1p and Sec61p. Structures of Pma1p (**A, B**), Por1p (**C-F**), and Sec61p (**G, H**) were homology modeled by HHPRED [15] using plasma membrane H^+^-ATPase from *Neurospora crassa* (1mhs_A) [16], the voltage-dependent anion channel VDAC1 from *Mus musculus* (4c69_X) [17], and Sec61 from *Canis lupus/Bos Taurus* (3jc2_1) [18] as template. Structural models were visualized by PyMOL and the position of crosslinks connecting the Cα atoms of amino acids were added manually based on the experimental data from pLink.

**Fig 5:**
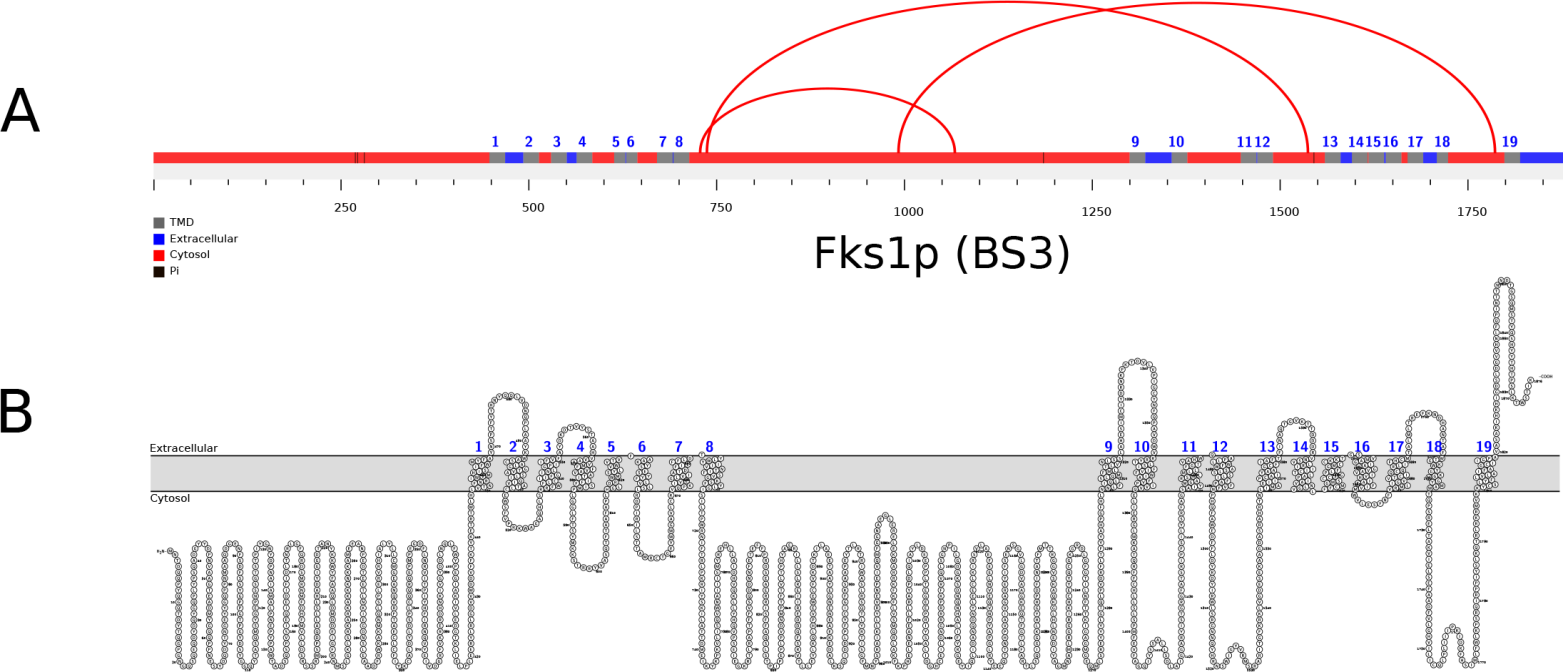
BS3 crosslinks mapped onto the topological model of Fks1p, the catalytic subunit of 1,3-beta-D-glucan synthase. **A,** Arch model of Fks1p indicating the positions of BS3 (red) XLs visualized using xVis. **B,** topology model of Fks1p proposed using the crosslinking data to validate a topology prediction visualized by Protter [19].

The position of XLs in Pma1p, Por1p, and Sec61p, abundant yeast MSPs for which a 3D structure can be modeled based on X-ray diffraction data published from orthologs of other species, are shown in Fig 4. For other proteins, for which no crystal structure is available, we drew linear “arch” models (Fig 5 and S2 Fig) combining a bioinformatically predicted topology (S3 Fig) using the xVis online server (https://xvis.genzentrum.lmu.de).

We also found XLs in several MSPs of the mitochondrial inner membrane and in Por1p, the porin of the mitochondrial outer membrane, all of which were reported to be present at high copy numbers even in cells grown on glucose [6, 7]. This was quite astonishing, since we had removed mitochondria by a preliminary centrifugation at 9’000 x g (see Materials and Methods).

### XLs are not frequently found on the luminal side of the secretory pathway

We did not encounter XLs involving luminal domains of the few MSPs of the secretory pathway we analyzed (S14 Table). However, in two entirely luminal membrane proteins, Gas1p and Zps1p, a total of 7 crosslinks were observed, demonstrating that such XLs do exist. To improve the yield of luminal crosslinks, we tried to permeabilize the microsomal membrane with mild detergents prior to crosslinking reactions. However, due to ion suppression resulting in a very low yield of detected crosslinked peptides, this approach was abandoned. We nevertheless could think of several potential reasons for the low frequency of XLs in luminal loops of MSPs. 1) The loops on the luminal side may be glycosylated and for this reason escape detection by pLink with the settings used here and be trypsin resistant. However, screening our mgf files for simple, non-substituted peptides from Wbp1p and Sec61p showed that overall trypsin had cleaved more potential sites on the luminal than the cytosolic side (74% vs. 62%, respectively). 2) It seemed conceivable that after being exposed to pH 11.5, membranes would form tight right-side-out vesicles that are impermeable for BS3 or Sulfo-NHS so that XLs in luminal loops cannot be formed. Indeed, it had been shown before that the translocation and GPI anchoring activity of a model protein in ER-derived microsomes resisted a 30 min incubation at pH 10.0 and that subsequent to high pH treatment the microsomal membranes formed sealed right-side-out vesicles so that translocated GPI proteins were resistant to proteinase K [20]. Indeed, in 3 proteins analyzed (Sec61p, Vph1p, and Wbp1p) 5 out of 31 possible sites were monosubstituted by BS3 on the luminal, 18 out of 42 on the cytosolic side. However, the number of XLs in our experiments are insufficient to determine whether crosslinking is significantly biased towards cytosolic loops.

Contrary to MSPs of the secretory pathway, some mitochondrial proteins do have XLs at both membrane surfaces. This is not only true for the outer membrane porin Por1p but equally for the inner membrane phosphate- and ADP/ATP exchangers Pet9p. This may be explained either by assuming that the inner mitochondrial membrane does not form closed vesicles after carbonate washing or that porin and maybe also Pet9p forms an open membrane channel, which may, even after carbonate washing, let the crosslinkers diffuse through the membrane (see models in S2 Fig and S14 Table). Interestingly, also in these cases, the XLs were rather luminal to luminal and cytosolic to cytosolic rather than luminal to cytosolic.

## Discussion and outlook

While protein crystallization is the by far best method to obtain high resolution structural information on membrane proteins, it is not always feasible and can be very time consuming. There are cases where proteins are found to change their membrane topology depending on the membrane’s lipid composition [21] or their subcellular localization [22, 23]. Amongst existing methods, such as dual topology reporters (DTR; [24]), substituted cysteine accessibility methods (SCAM; [25]), and epitope insertion techniques, all are quite laborious and not error-free (for review see [26]). Thus, to monitor dynamic changes in protein topology or topology in different environments, faster and less work-intensive methods would be certainly desirable. Our report establishes chemical crosslinking entire membranes as a novel methodological approach to obtain some low resolution structural information on the topology of MSPs, yielding novel XL-MS-based topological information for 17 integral membrane proteins (Akr1p, Fks1p, Gas1p, Ggc1p, Gpt2p, Ifa38p, Ist2p, Lag1p, Pet9p, Pma1p, Por1p, Sct1p, Sec61p, Slc1p, Spf1p, Vph1p, Ybt1p) (S14 Table). The method clearly only can work when the protein under investigation is very abundant, naturally or because of strong overexpression. Pma1p, the sole protein for which we obtained reasonable data has been found to be present at 1’260’000 and 113’952 copies per cell by two different reports [6, 7]. Thus, overexpression of a given protein under study should certainly be in the range of < 100’000 copies per cell.

Here we only tried moderate overexpression because 11 strains, each overexpressing one single protein under the *GAL1* promoter, were pooled before preparing membranes and therefore, each protein was only overexpressed at 1/11 of the level it could have reached if membranes were made from a single strain.

The use of carbonate along the lines described in our protocol cannot be recommended. The removal of ribosomes prior to the addition of crosslinkers was found to be necessary to see XLs in MSPs, but on the other hand the identification of non-crosslinked peptides (see Proteome Software Scaffold.sf3 files (available at the ProteomeXchange server, accession PXD006707) shows that large amounts of ribosomal proteins and of cytosolic enzymes (e.g., enzymes of glycolysis) were still present after carbonate washes. Thus, alternative methods of ribosome removal and washing of membranes ought to be investigated. However, the validation of the crosslinking data with the crystal structures of Pma1p, Sec61p, and Por1p indicates that the harsh carbonate treatment that we employed did not alter the membrane topology of these proteins.

Further improvements of this approach could certainly also be obtained through the purification of specific organelles such as mitochondria, peroxisomes, lipid droplets, autophagosomes or vacuoles prior to crosslinker treatments, first, because these organelles are not contaminated by ribosomes and specially because such organelles also can massively be induced under appropriate conditions. For example, in our experiments mitochondria were not induced and even removed by a low speed centrifugation step, and yet, numerous XLs could be identified in abundant mitochondrial proteins. Such potential improvements are currently under investigations.

## Supporting information

S1 Table Experimental variations. (XLSX)

S2 Table Statistics of crosslinks found by StavroX. (XLSX)

S3 Table Promiscuity amongst EDC crosslinks found by StavroX. (XLSX)

S4 Table List of proteins analyzed. (XLSX)

S5 Table Pma1p crosslinks found by StavroX in inappropriate mgf files. (XLSX)

S6 Table Pma1p crosslinks found by pLink in inappropriate mgf files. (XLSX)

S7 Table Filtering criteria for pLink. (XLSX)

S8 Table Pma1p: BS3-d0 and BS3-d4 crosslinks. (XLSX)

S9 Table Distances between crosslinked amino acids found by pLink. (XLSX)

S10 Table Crosslinks found by StavroX. (XLSX)

S11 Table Crosslinks found by StavroX in absent proteins. (XLSX)

S12 Table Crosslinks found by StavroX in distant proteins. (XLSX)

S13 Table Crosslinks found by StavroX in adjacent proteins. (XLSX)

S14 Table Crosslinks found by pLink. (XLSX)

S15 Table Crosslinks found by pLink in absent proteins. (XLSX)

S1 Fig Fragmentations of crosslinks found by StavroX. (PDF)

S2 Fig Crosslinks found by pLink. (PDF)

S3 Fig Putative topology of analyzed proteins. (PDF)
