# Supplementary material for: Chemical crosslinking and mass spectrometry to elucidate the topology of integral membrane proteins
Source: PLoS One. 2017 Oct 26;12(10):e0186840. doi: 10.1371/journal.pone.0186840 (PMC5658093; doi:10.1371/journal.pone.0186840)

S1 Fig. Fragmentation of crosslinks found by StavroX

S1A : Crosslinks obtained by EDC with at least 7 amino acids per peptide were inspected in the .zhrs files generated by StavroX. Screen shots were taken for individual links allowing to see their a-, b-, and y-type fragment ions. The intensity of fragment ions is reported by the color coding of the fragmentation site according to the scale shown below. Each fragmentation picture is headed by a series of numbers such as 7779, 570.869, 15-1, 124/81, 1.26, referring respectively to experiment number, m/z value, position of amino acid in peptide 1 - position of amino acid in peptide 2 forming the link, score/FDR (false discovery rate), deviation of m/z from theoretical (in parts per million, ppm). Occasionally the experimental number is preceded by the rank of the XL in the StavroX .zhrs file when XLs are order according to score (highest to lowest). The experiment number allows to find the XL in the corresponding mgf files at (ProteomeXchange accession number: PXD006707). for further analysis of MS data. Each page reports data for a single protein. In many pages the protein sequence is shown as a bar with the transmembrane domains (TMDs) in grey, the predicted luminal loops in blue, and the cytosolic ones in red. The XLs are indicated by arches above the bar. This bar represents a model that was created using predictions made by different prediction algorithms or by comparing the sequence of the yeast protein with known crystals of similar proteins in other species. In the top panel (BS3) the TOPCONs consensus model {Tsirigos et al., 2015, Nucleic Acids Res, 43, W401-7} is shown directly below the red-grey-blue bar unless indicated otherwise. Below the TOPCONS consensus there is a bar that shows the most conserved residues in red. These conservations were compiled by inspecting the data at NCBI (https://www.ncbi.nlm.nih.gov/Structure/cdd/). The phosphorylations reported in SGD (http://www.yeastgenome.org) are shown as vertical black lines in the red-grey-blue-(green) bar and highlighted by yellow balls below the bar. The scale below the bars indicates the length of the amino acid sequence in hundreds. Particular aspects of a model are discussed at the bottom of each page.
Only XLs that have scores higher than the FDR were considered and amongst those we chose for each protein shown those (few) that showed the best fragmentation. Some ions proposed by StavroX invoke impossible XLs, e.g. because the two peptides are overlapping or because the link involves a peptide's C-terminal lysine residue that could not have been cleaved by trypsin if it really were crosslinked. StavroX also frequently reports XLs between two adjacent peptides, but their fragmentation was only occasionally depicted as they usually do not contain topological information. These XLs are marked as adjacent (adj.) in the header. (When adjacent peptides are invoked for an EDC generated link, more in depth analysis of fragmentation patterns is required to decide if there is a XL between the two separated peptides or if the ion was generated by a single peptide, in which peptide 1 is linked via its natural peptide bond to peptide 2 as the mass of the XL is the same as the one of a single, continuous, non-crosslinked peptide.)
S1B (last page): As S1A but for BS3-generated XLs. Only Pma1p was found to have reasonably fragmented ions.

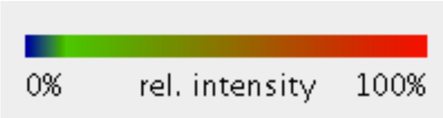

S1A Fig. Cds1p, 8865 copies per cell

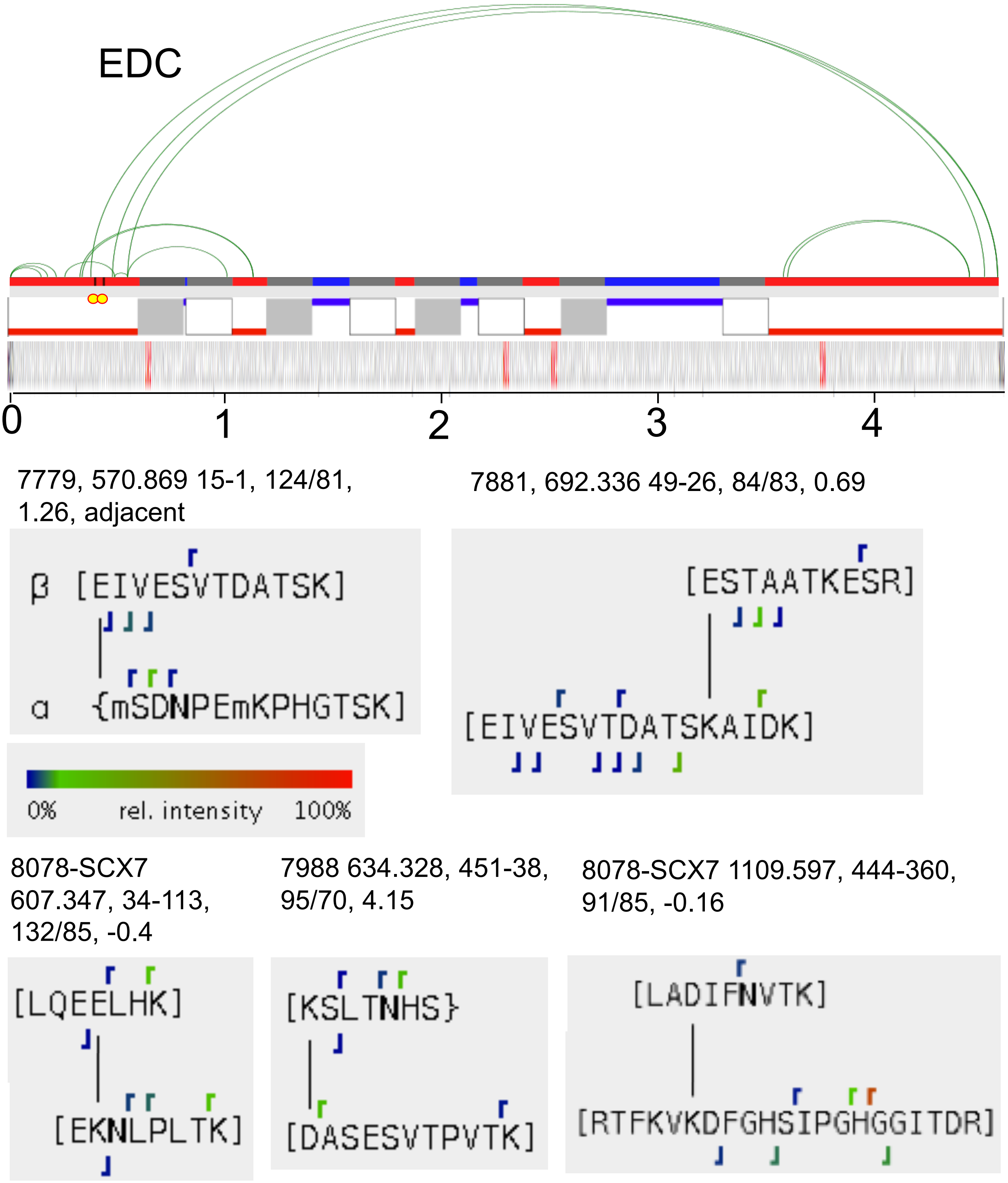

Cds1p synthesizes CDP-diacylglycerol, from which cells make phosphatidyl-serine and -inositol. The recent crystallization of a Cds1p homolog from *Thermotoga maritima* {Liu et al., 2014, Nat Commun, 5, 4244} confirms the membrane topology of the TOPCONS model, whereby a last 9th C-terminal TMD of that protein is not very homologous with yeast Cds1p and not followed by a large soluble domain that is present in yeast Cds1p. The yeast C-terminus is extensively crosslinked to the N-terminus and we therefore assume that this last, 9th TMD of the *T. maritima* protein does not exist in yeast. The EDC XLs depicted are not of high quality but do not contradict the structure model suggested by the crystal.

S1A Fig. Flc1p, no copy numbers reported

EDC

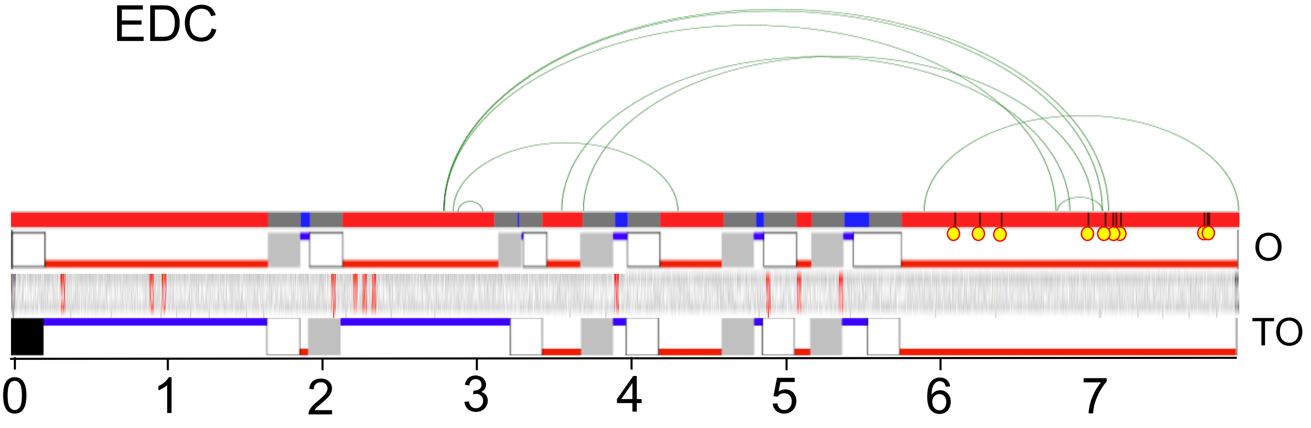

7881, 760.003, 305-291,
99/80, -1.11, adjacent

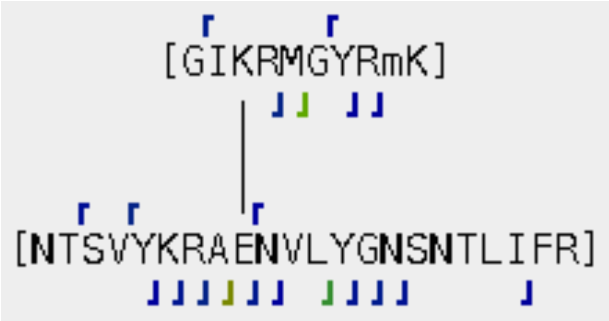

7881, 1027.56, 305-291,
370-705, 150/80, 2.54

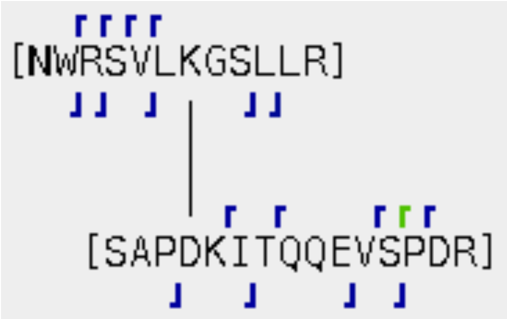

8078-SCX6, 904.141,
705-280, -2.25

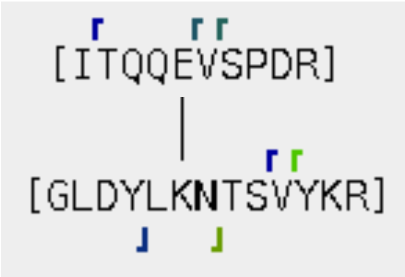

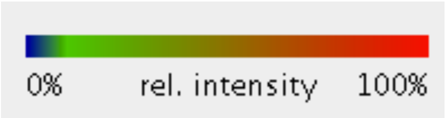

Flc proteins have been proposed to be FAD transporters, calcium channels and lipid biosynthesis regulators of the ER {Protchenko et al., 2006, J Biol Chem, 281, 21445-57; Vazquez et al., 2016, PLoS Genet, 12, e1006160}. EDC-XLs are of intermediate quality and the one of the top row are promiscuous. O and TO show OCTOPUS and TOPCONS consensus topologies.

S1A Fig. Flc2p, no copy numbers reported

EDC

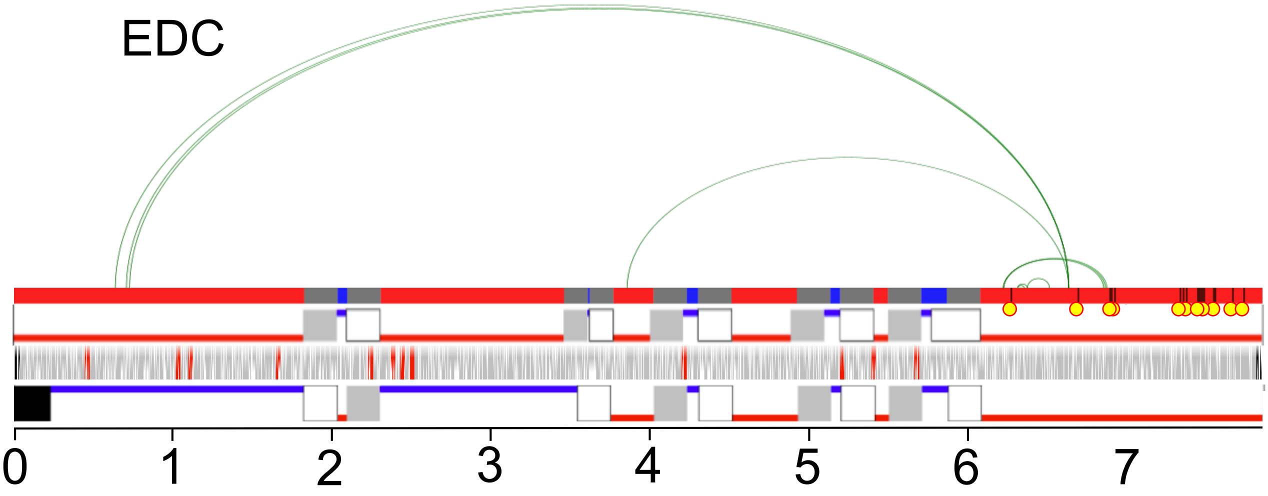

7824, 1189.816, 662-64, 82/70, 0.95

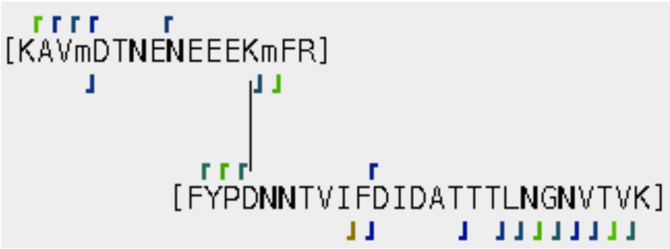

7824, 632.689, 385-662,
70/70, -0.14

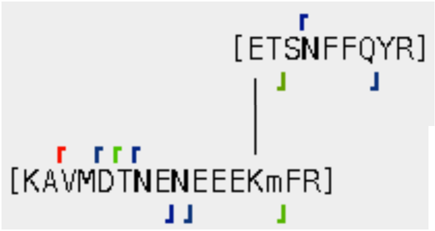

The XLs of Flc2p, as the ones of Flc1p, can only be fitted to the OCTOPUS prediction shown below the red-grey-blue bar in the BS3 panel, but they disagree with the TOPCONS consensus shown below the conserved residues' bar and with all other algorithms that lead to that consensus. However, the OCTOPUS model risks to be true since a global mass spectrometric analysis of all N-glycosylated residues of yeast reports 5 N-glycosylations between amino acids 65 and 156 {Protchenko et al., 2006, J Biol Chem, 281, 21445-57}, so this part must be luminal, as indicated by the TOPCONS consensus.

S1A Fig. Flc3p, no copy numbers reported

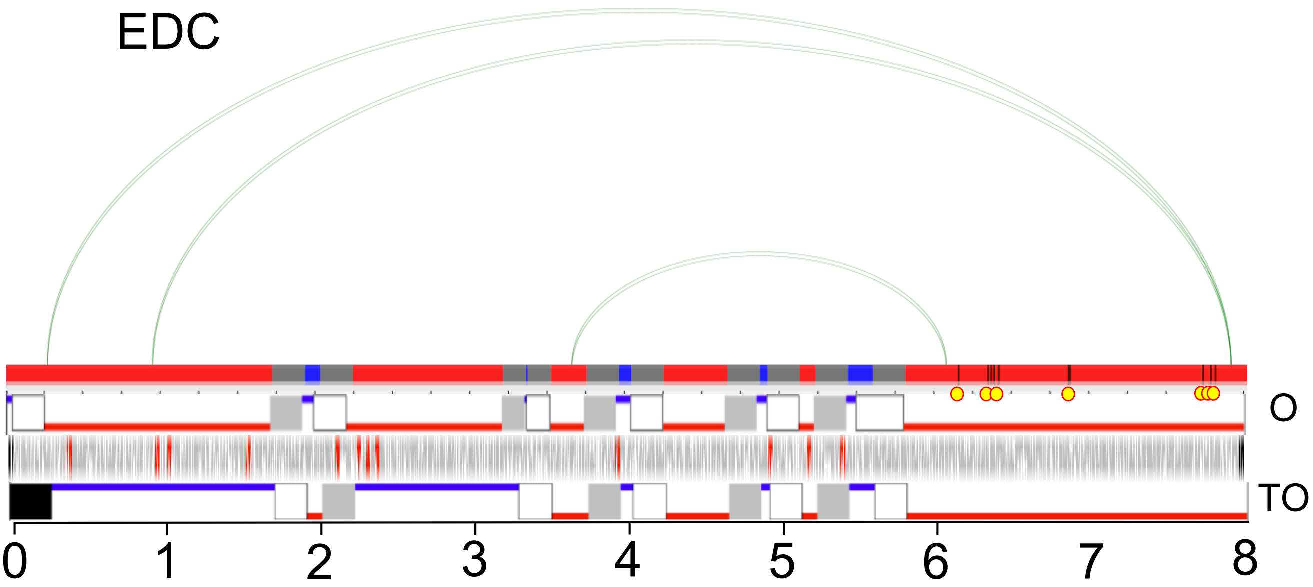

7824, 958.813, 365-607, 84/73, 1.34

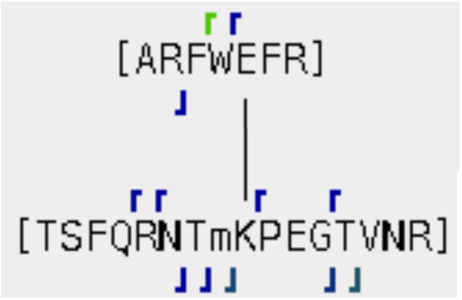

The XLs of Flc3p, as the ones of Flc1p, can only be fitted to the OCTOPUS (O) prediction, but they disagree with the TOPCONS (TO) consensus.

S1A Fig. Gpt2p, overexpressed

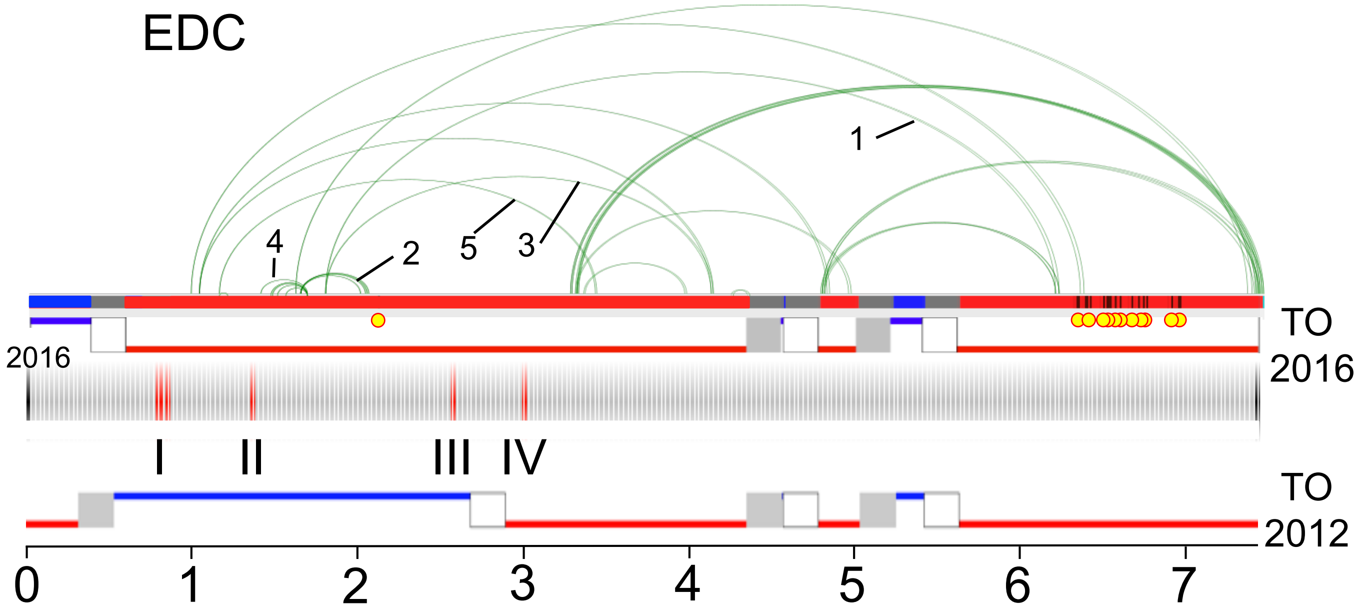

1=7779, 588.924, 620-179, 145/116, -1.3

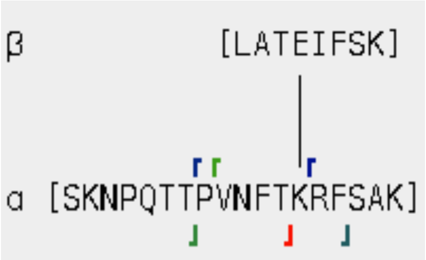

2=7881, 694.871 164-203, 181/116, -0.9

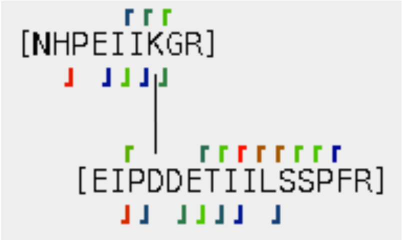

3=8078-SCX5 614.327 412-179,

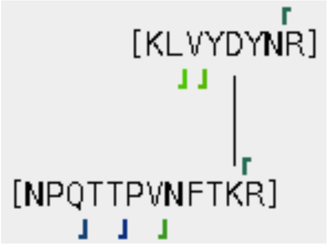

4=8078-SCX6, 754.799 168-146, 193/79, 0.95, adj.

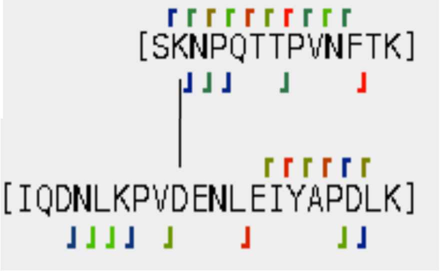

7881, 916.215, 115-120, 329/116, 4.54, adjacent, promiscuous

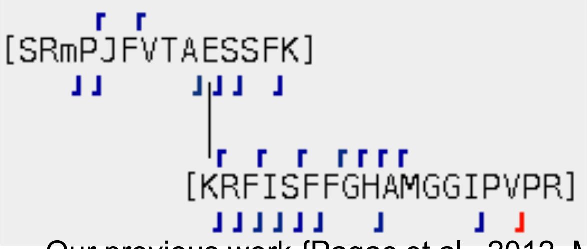

5=8078-EDC, 1121.255 342-115, 91/87, -3.4

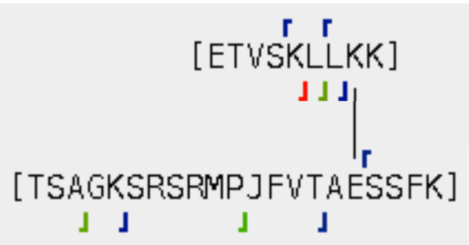

Our previous work {Pagac et al., 2012, Mol Microbiol, 86, 1156-66} had established tentative topologies for the conserved amino acids in Gpt2p confirming the global TOPCONS prediction (TO) of the year 2012 (shown at the bottom), which had the conserved motifs I – III in the ER lumen, the IVth, often lacking, in the cytosol. (TOPCONS algorithms are continuously modified and improved, so that predictions over the years are changing). Our "mass spectrometric detection of crosslinked peptides" approach now suggests a different topology for Gpt2p than what we had obtained through biochemical methods (DTR, identification of protease protected fragments and SCAM). The reported EDC XLs show reasonable fragmentation frequency and are only compatible with the TOPCONS consensus of 2016, which places the 4 conserved motifs into the cytosol.

S1A Fig. Ist2p, 4937 copies per cell

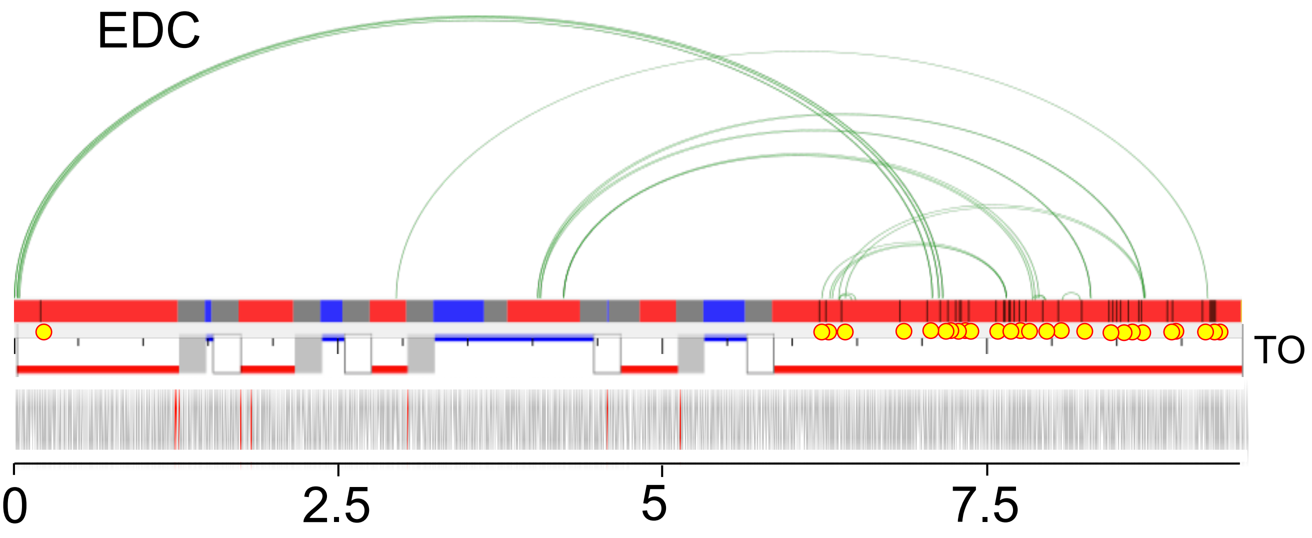

7779, 834.438 636-646, 144/106, -1.11, adj..

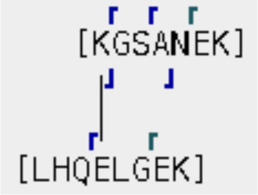

7881, 541.805 795-787, 145/130, 0.65, adj..

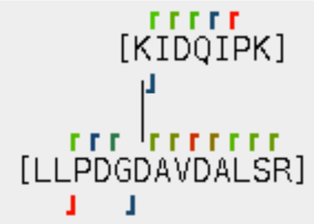

8078-SCX3, 703.347, 404-830, 151/108, -1.78

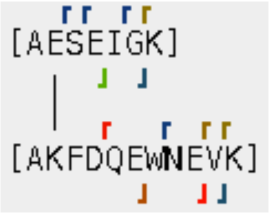

7988, 1027.56, 765-631, 333/214, 1.9

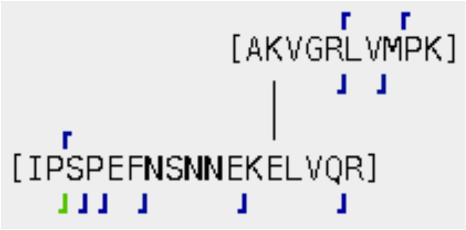

Or alternatively, 7988, 1027.56 765-623, 246/214, -1.68

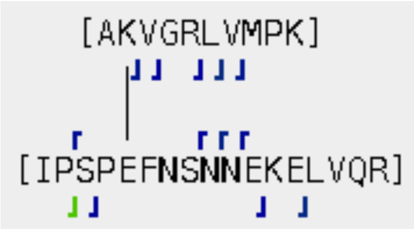

7780, 402.719, 636-872, 188/156, -3.13

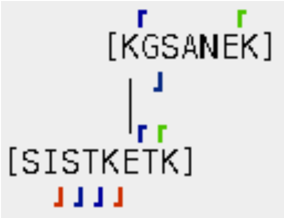

Ist2p is part of a membrane protein complex of the ER that tethers the cortical ER to the plasma membrane, wherein the C-terminal lipid binding domain of Ist2p plays a critical role {Manford et al., 2012, Dev Cell, 23, 1129-40}. The last XL in the top row is not compatible with the extended luminal domain between amino acids 325 and 450 of the TOPCONS consensus model (TO) but can be accommodated by our model having 10 TMDs, which is drawn based on the homology of Ist2p with the recently crystallized TMEM16 lipid scramblase of *Nectria Haematococca* {Brunner et al., 2014, Nature, 516, 207-12}.

S1A Fig. Pet9p, 58’239 copies per cell, EDC-XLs

7779, 767.423, 18-20, 158/93, 0.56, adj.

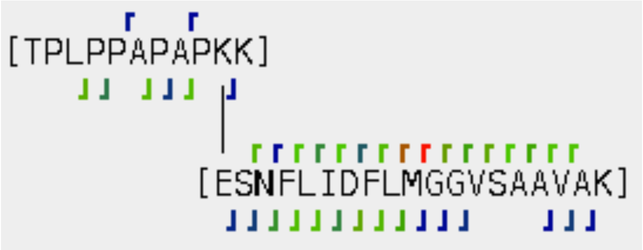

7881, 561.552, 184-178, 140/110, 2.35

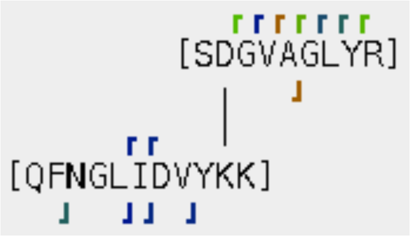

7881, 767.145, 81-108, 131/110, 1.41 1.41

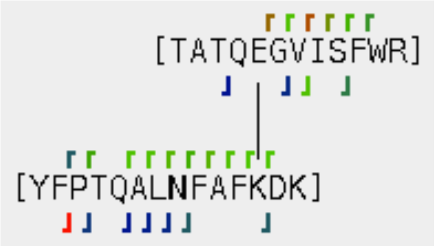

8078-SCX5, 558.804 160-56 132/61, 1.33

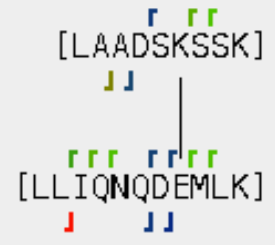

8078_SCX7, 824.436 120-178, 92/62, -4.54

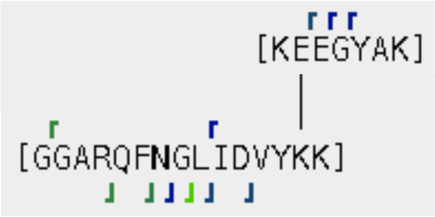

Pet9p is the major ADP/ATP carrier of the mitochondrial inner membrane. The XLs 767.423 and 824.436 connect residues at opposite sides of the membrane, possibly because of the hydrophilic channel passing through this pore.

S1A Fig. Pma1p, copy number 1’260’000/113’952 , EDC-XLs

Experiment 7881

6, 834.42 644-634, 234/122
0.28, adjacent peptides

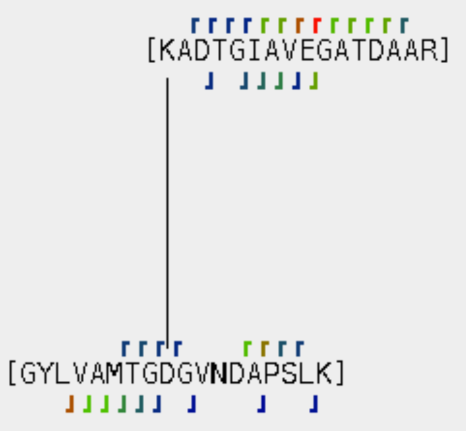

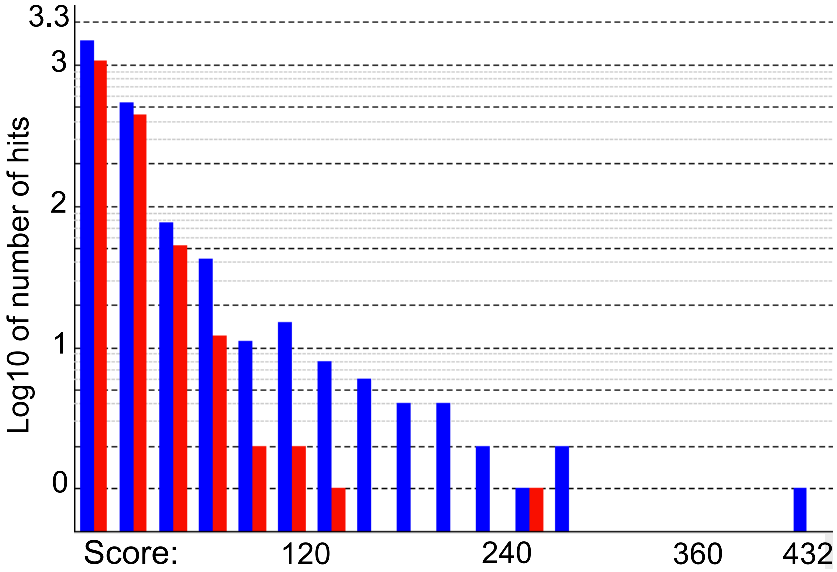

7, 916.215 420-518, 171/122, -
0.14, promiscuous

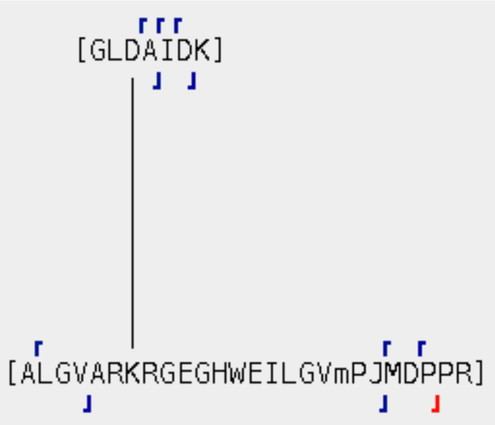

9, 732.631, 457-646,
222/122, -1.39

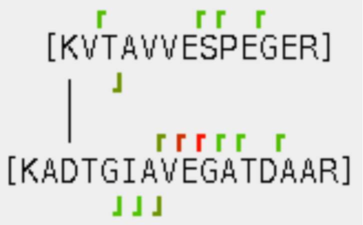

13, 833.118, 620-644, 192/122, 1.0

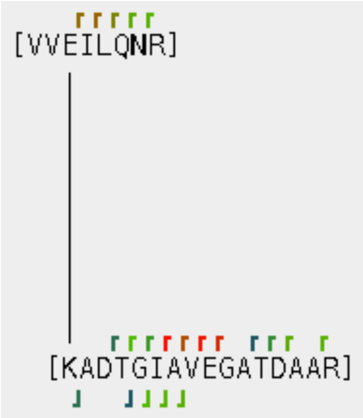

12, 744.636, 582-644, 196/122, 1.27

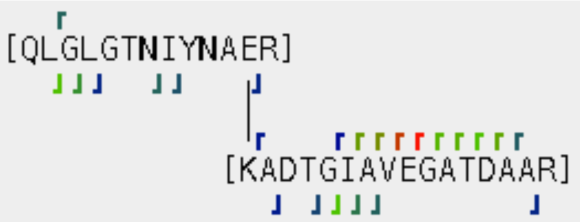

8078-SCX6, 3, 833.118, 620-644,
180/117, 1,31; same peptides as
in 13 from another experiment

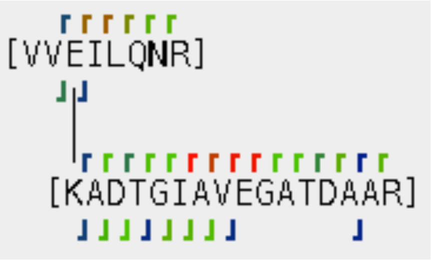

8078-SCX6, 1, 625.091, 620-644,
245/117, 2.73; same peptides as in 13 and
to the left with other charge (z)

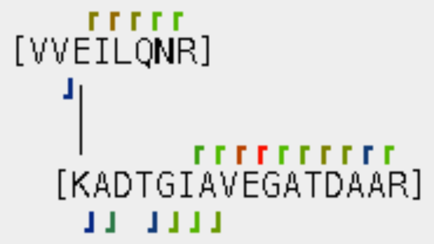

S1A Fig. Pma1p-EDC, cont. of experiment 7881, EDC-XLs

14, 700.609, 457-652
190/122, 0.47

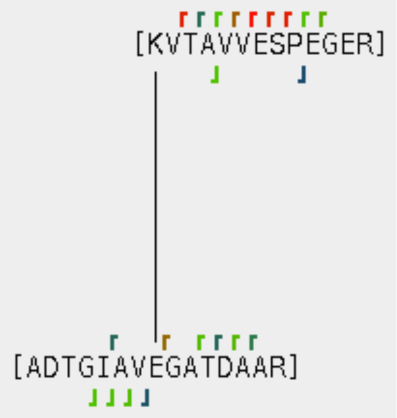

or 457-646

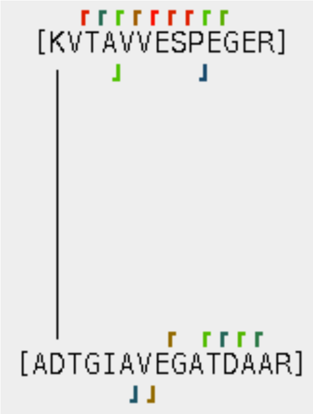

25, 700.609,
463-644, 154/122, 0.74

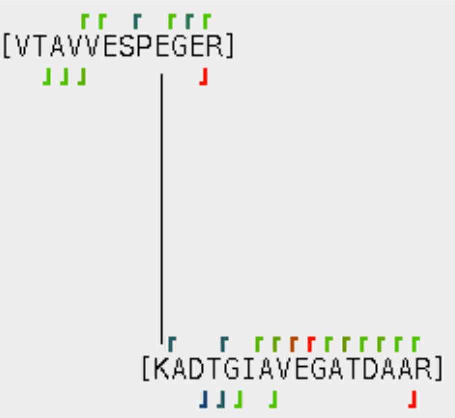

22 763.886, 582-566,
161/122, 0.69, adj.

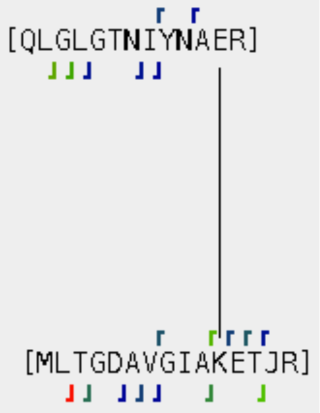

27, 543.571, 504-
474, 149/122, 0.92

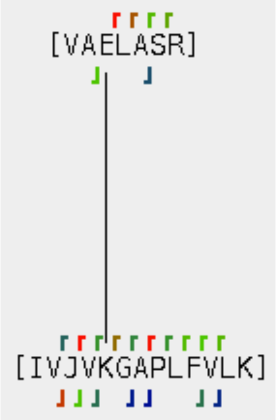

34, 515.789, 428-
463, 133/122, 0.32

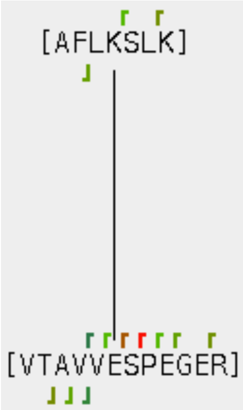

35, 500.772,
437-466, 132/122, -0.2

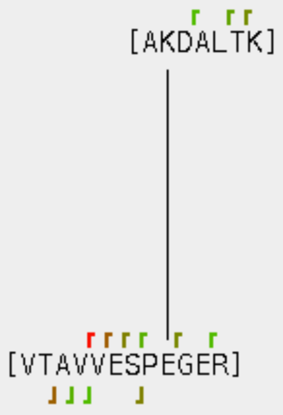

500.772 437-466,
117/64, 1.01; same
as 35 in another exp.
(6 in 8078-SCX4)

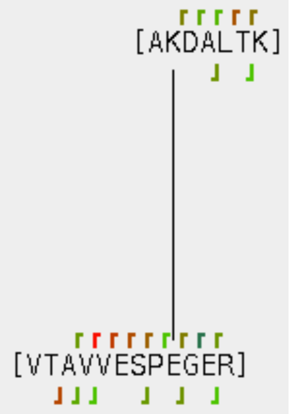

S1A Fig. Pma1p, experiment 8078-SCX4, EDC-XLs

1, 615.053, 917-652, 359/64, 2.29

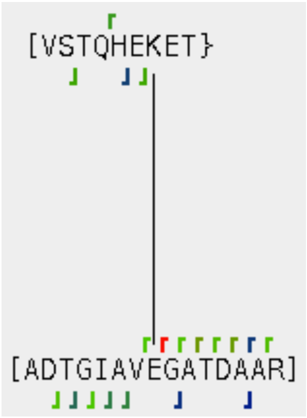

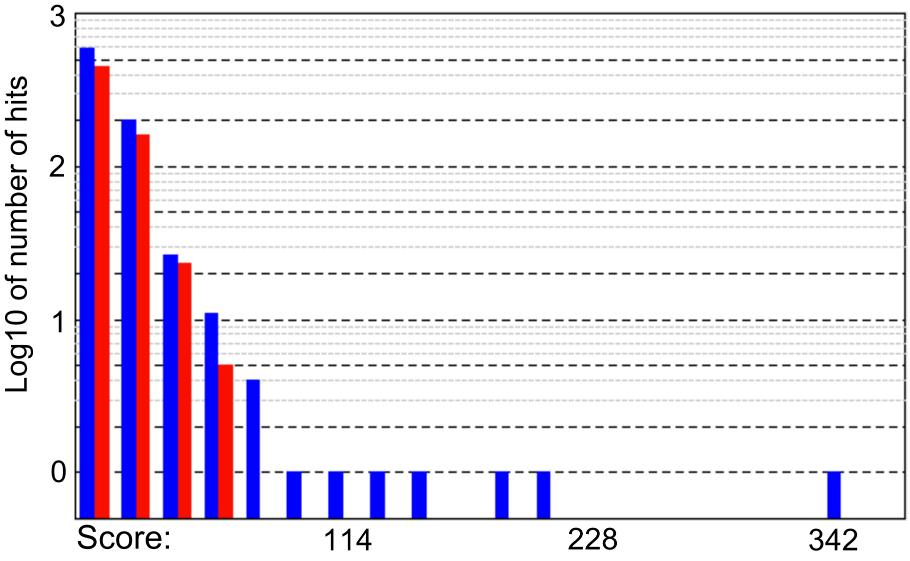

2, 566.049, 385-652, 222/64, 1.3

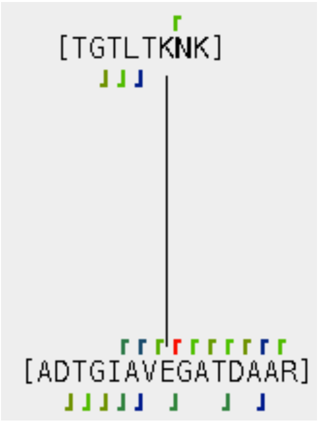

3, 480.748, 437-538, 191/64, 1.55

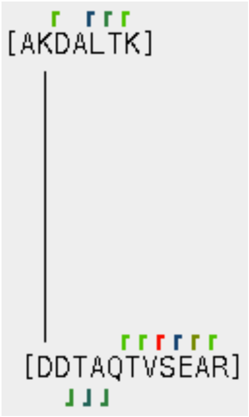

4, 679.347, 385-538, 162/64, 0.28

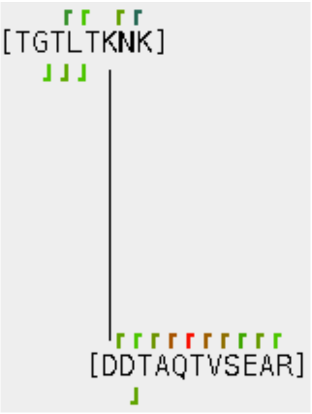

5, 509.762, 385-538, 149/64, 1.18

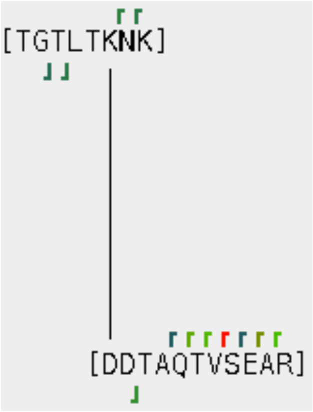

6, 500.772, 437-466, 117/64, 1.01

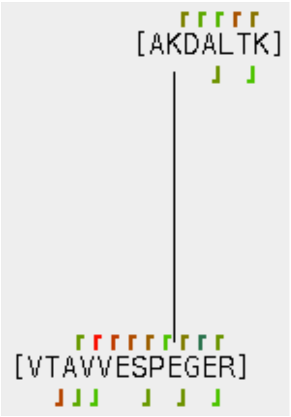

7, 754.397, 385-652, 97/64, 1.79

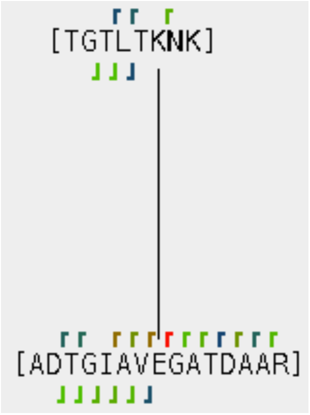

S1A Fig. Pma1p, experiments 7779, 7988 and 8078-SCX7, EDC-XLs

7779, 1, 821.45 892-616, 147/112, -0.49

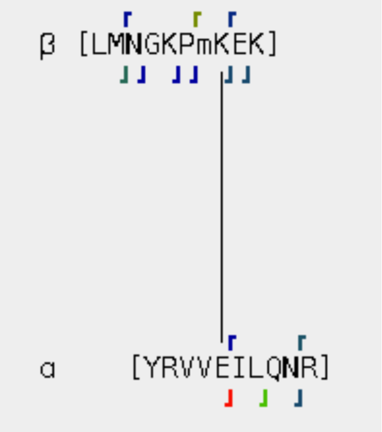

7988, 2, 619.823 539-555, 176/172, -0.05

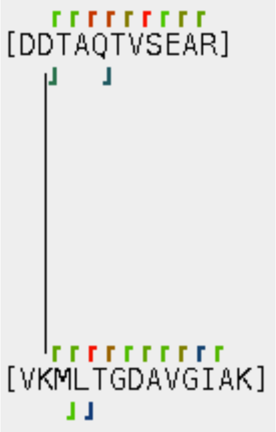

8078-SCX7, 1, 932,472, 893-566, 160/117, 2.77

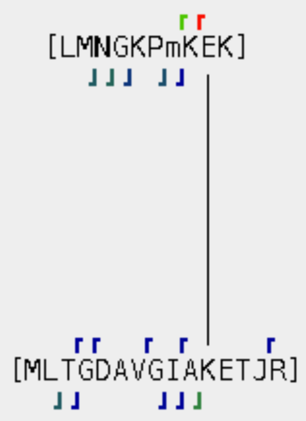

Pma1p represents the main plasma membrane proton-pumping ATPase in order to regulate the cytoplasmic pH and generate a plasma membrane potential. Many highly fragmented XLs are identified in this very abundant membrane protein. Many ions are found in numerous different experiments, the same XLs are often found with different charges z, resulting in different m/z values of the precursor ion.

S1A Fig. Por1p, 125’951 copies per cell, EDC-XLs

7881, 727.645, 236-220, 99/ 64, -0.15

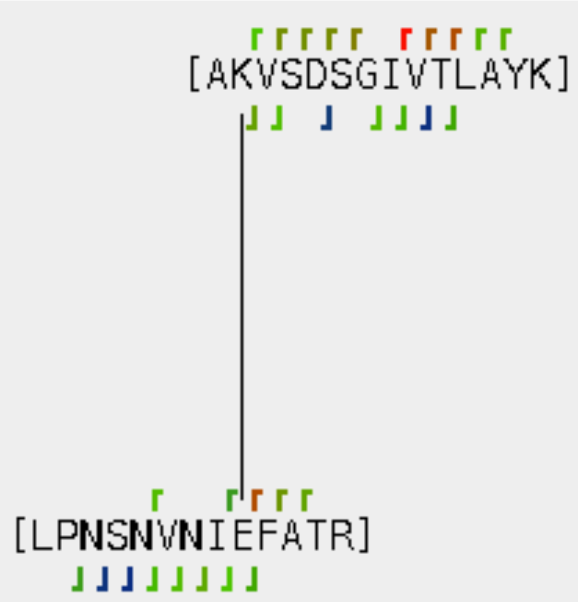

Por1p forms large pores in the mitochondrial outer membrane. In spite of its abundance, only a single well fragmented XL was observed, linking two residues which are not far apart, neither in the primary sequence nor the 3D structure.

S1A Fig. Sec61p, 24’800 copies per cell, EDC-XLs

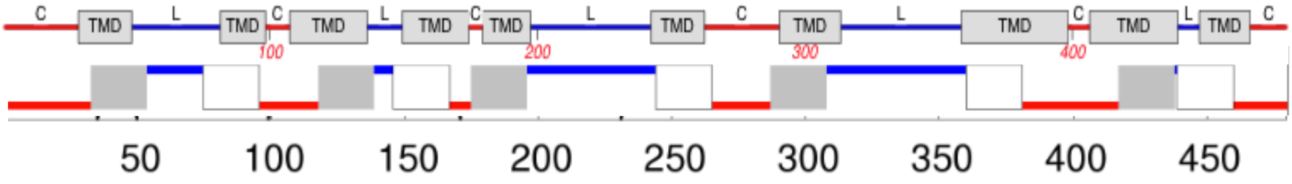

7779,1, 487.766, 393-397, 111/ 59, 0.4, adjacent

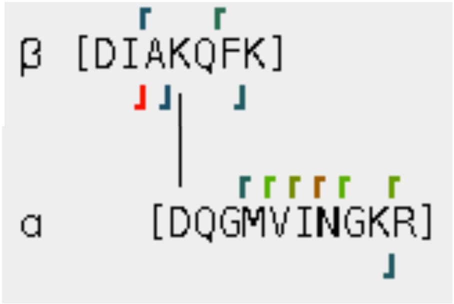

7780, 1, 487.766, 393-397, 254/64, 0.55, adj.

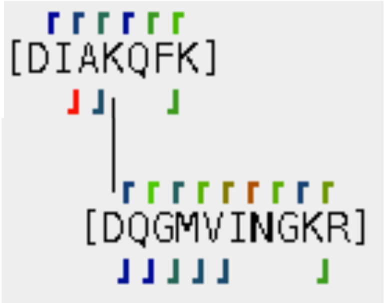

7780, 6, 641.673, 393-481 72/64, 0.26

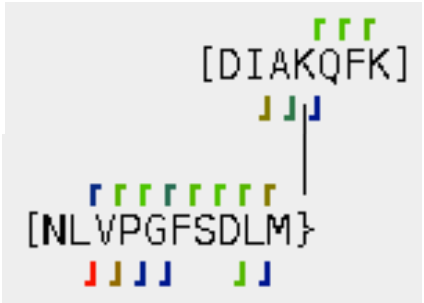

Sec61p forms the conserved ER protein translocation channel.

S1A Fig. Sur2p, 54’300 or 3003 copies per cell, EDC-XLs

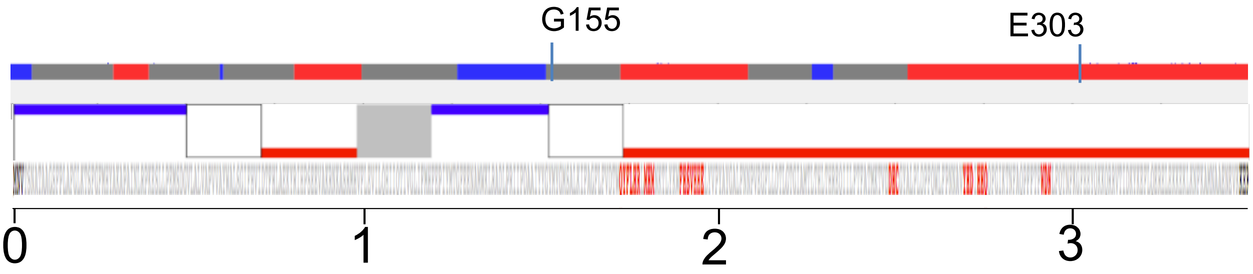

7780, 1, 602.516 323-333, 119/57, 0.71

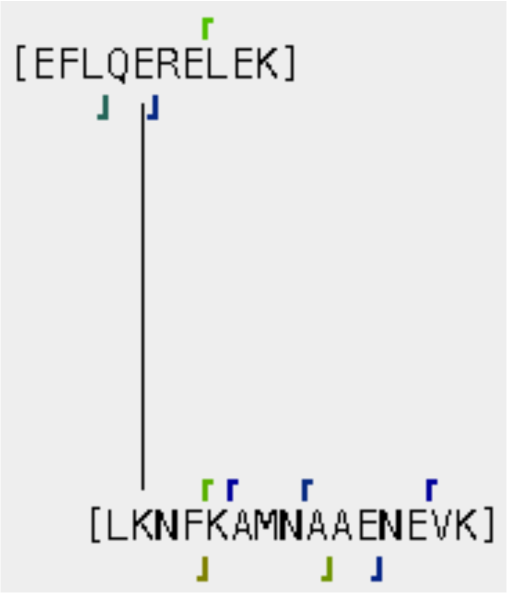

7780, 2, 703.721, 315-325, 117/57, -3.85

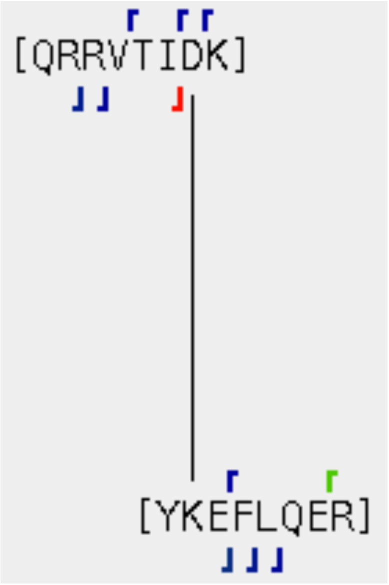

7824,1, 654.338, 329-344, 106/59, -0.5

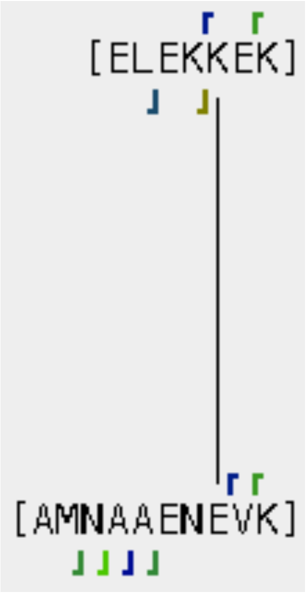

Sur2p hydroxylates dihydrosphingosine. EDC-XLs are not very well fragmented. Also, none of them contains topological information.

S1B Fig. Pma1p, BS3-XLs

Pma1p-BS3

Pma1p, n.a., 686.096, 917-644
score 208, FDR 120, dev 1.34

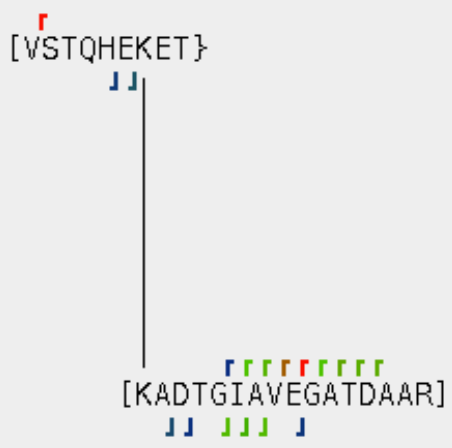

Pma1p, n.a., 756.084, 437-558
score 105, FDR 100, dev 0.31

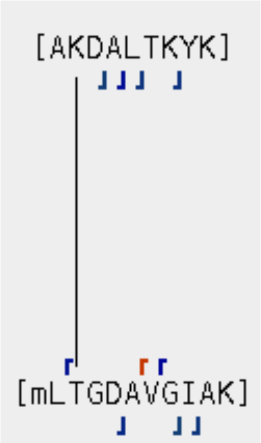

Pma1p, 811,143, 919-894
score 158, FDR 118, dev 1.2

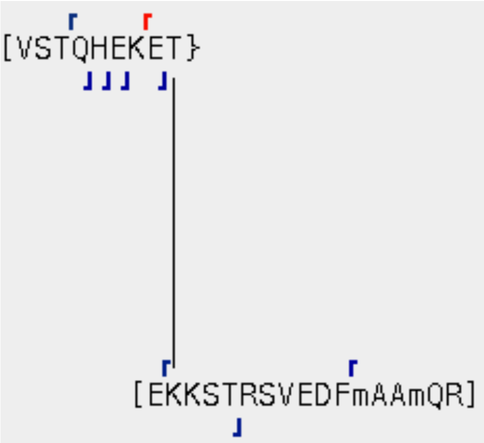

Pma1p, 650.841, 917-457
Score 122, FDR 97, dev 1.95

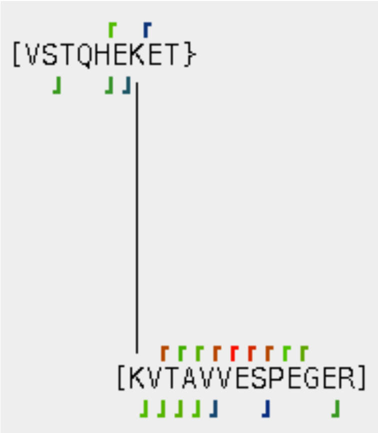

Supplement: S1 Fig — (PDF) [file pone.0186840.s016.pdf]
