# Supplementary material for: Chemical crosslinking and mass spectrometry to elucidate the topology of integral membrane proteins
Source: PLoS One. 2017 Oct 26;12(10):e0186840. doi: 10.1371/journal.pone.0186840 (PMC5658093; doi:10.1371/journal.pone.0186840)
Supplement: S2 Fig — (PDF) [file pone.0186840.s017.pdf]

S2 Fig. Crosslinks found by pLink

Crosslinks generated by BS3 or EDC as identified by pLink are shown as arches connecting the various loops. Each page reports data for a single protein. The XLs obtained with BS3 and EDC are in separate panels. In each panel the sequence is shown as a bar with the TMDs in grey, the predicted luminal loops in blue, and the cytosolic ones in red. The XLs are indicated by red arches above the bar. This bar represents a model that was created using predictions made by different prediction algorithms or by comparing the sequence of the yeast protein with known crystals of similar proteins. The TOPCONs consensus model is shown below the red-grey-blue bars unless indicated otherwise {Tsirigos et al., 2015, Nucleic Acids Res, 43, W401-7}. Above the TOPCONS consensus there is a bar that shows the most conserved residues in red. These conservations were compiled by inspecting the data at NCBI (https://www.ncbi.nlm.nih.gov/Structure/cdd/). The phosphorylations reported in SGD (http://www.yeastgenome.org) are shown as vertical black lines in the red-grey-blue bars. The scale below the TOPCONS panel indicates the length of the amino acid sequence in hundreds. Particular aspects of a model are discussed at the bottom of each page. The Protter topology models are shown at the bottom of each page (phosphorylated residues are highlighted in red).

Fks1p. Fks1p is a glycosyltransferase that synthesiszes b1,3-glucans from UDP-Glucose at the cytosolic surface of the plasma membrane and at the same time extrudes the polymer through the plasma membrane into the periplasmic space. The XLs fit the TOPCONS consensus. We however drew the model slightly differently in order to bring the phosphosite at S1822 to the cytosol. A previous study using dual topology reporter assays (DTR) resulted in a model that fits XLs quite well except that these authors found 1738 in the ER lumen, whereas our model predicts 1786 to be cytosolic {Johnson and Edlind, 2012, Eukaryot Cell, 11, 952-60}.

Gpt2p. Our previous work {Pagac et al., 2012, Mol Microbiol, 86, 1156-66} had established tentative topologies for the conserved amino acids in Gpt2p confirming the global TOPCONS consensus prediction of the year 2012 (shown at the bottom of the BS3 panel). These experiments placed the conserved motifs I – III in the ER lumen, the IVth, often lacking, in the cytosol. Yet, the TOPCONS consensus prediction of 2016 shown higher up places these conserved motifs into the cytosol. (TOPCONS algorithms are continuously modified and improved, so that predictions over the years are changing). Our "mass spectrometric detection of crosslinked peptides" approach has not produced any XLs between different loops that would allow to discriminate the two models. The phosphosite at S211, determined without blocking the proteasomal protein degradation, favors the cytosolic location of motifs I – III. This is also supported by a XL linking the C-terminus to amino acid 206 in the closely related Sct1p (see below). The C-terminus was determined to be cytosolic with 92% probability by {Kim et al., 2006, Proc Natl Acad Sci U S A, 103, 11142-7}.

Sct1p. Sct1p is a close homolog of Gpt2p, for which our previous work {Pagac et al., 2012, Mol Microbiol, 86, 1156-66} relying on DTR-methods, identification of protease protected fragments and SCAM proposed that the conserved motifs I – III reside in the ER lumen. The C-terminus was determined to be cytosolic with 90% probability {Kim et al., 2006, Proc Natl Acad Sci U S A, 103, 11142-7} and carries numerous phosphosites. Therefore, the XL

between K206 and K573, which resides in this C-terminus, now suggests that motifs I - III reside in the cytosol. The XLs cannot distinguish between the TOPCONS consensus and the OCTOPOS prediction of the same server.

Slc1p. Our previous work had established tentative topologies for the conserved amino acids in Slc1p confirming the TOPCONS consensus model shown below the top bar {Pagac et al., 2011, J Biol Chem, 286, 36438-47}. SCAM and DTR methods placed the active site motif I of Slc1p into the lumen of the ER but motifs II – IV into the cytosol. (Motifs motifs I – IV placed according to {Yamashita et al., 2007, Biochim Biophys Acta, 1771, 1202-15} are indicated below the conserved residues' bar.) Our former topology was non-satisfactory in as much as the crystal structure of the distantly related soluble chloroplast glycerol-3-phosphate acyltransferase of *Cucurbita moschata* had motifs I – III in close proximity forming a surface pocket thought to accommodate glycerol-3-phosphate {Slabas et al., 2002, J Biol Chem, 277, 43918-23; Tamada et al., 2004, Acta Crystallogr D Biol Crystallogr, 60, 13-21}. The single XL found in Slc1p does not disagree with the TOPCONS consensus, but contradicts the PolyPhobius model shown below the bar of conserved amino acids that would place all conserved residues into the cytosol. A second TMD immediately following the first TMD as proposed by Phyre2 algorithm was introduced in our model (top bar). This model would not be in contradiction with the single XL but have all conserved motifs in the cytosol.

Spf1p. Spf1p is a P-type ATPase involved in the transport of divalent cations through the ER membrane. TMDs 3 – 5 are also rendered likely by the homology with a crystal structure according to TOPCONS. The XLs obtained are not interesting topologically, since they only connect amino acids within predicted loops.

Vph1p. Vph1p is a subunit of the V0 complex of the vacuolar, ATP-dependent proton pump required for vacuolar acidification. Only the N-terminus got crosslinked, whereby all TOPCONS algorithms predict the N-terminus to be lumenal. The structure was however drawn with the N-terminus in the cytosol because of a previous study using conventional biochemistry and placing the N-terminus into the cytosol {Toei et al., 2011, J Biol Chem, 286, 35176-86}.

Ybt1p. Ybt1p is a vacuolar ATP binding cassette membrane transporter involved in detoxification. Only predicted cytosolic domains seem to be crosslinked.

Fks1p

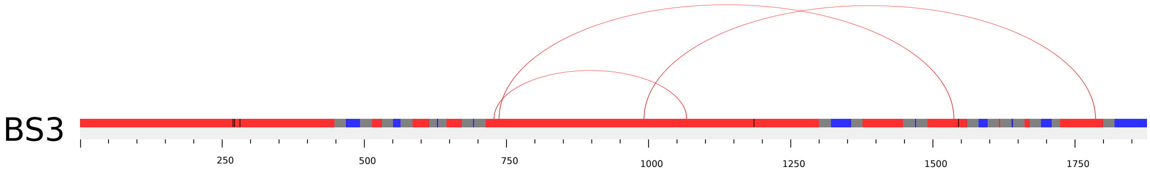
BS3
250
500
750
1000
1250
1500
1750

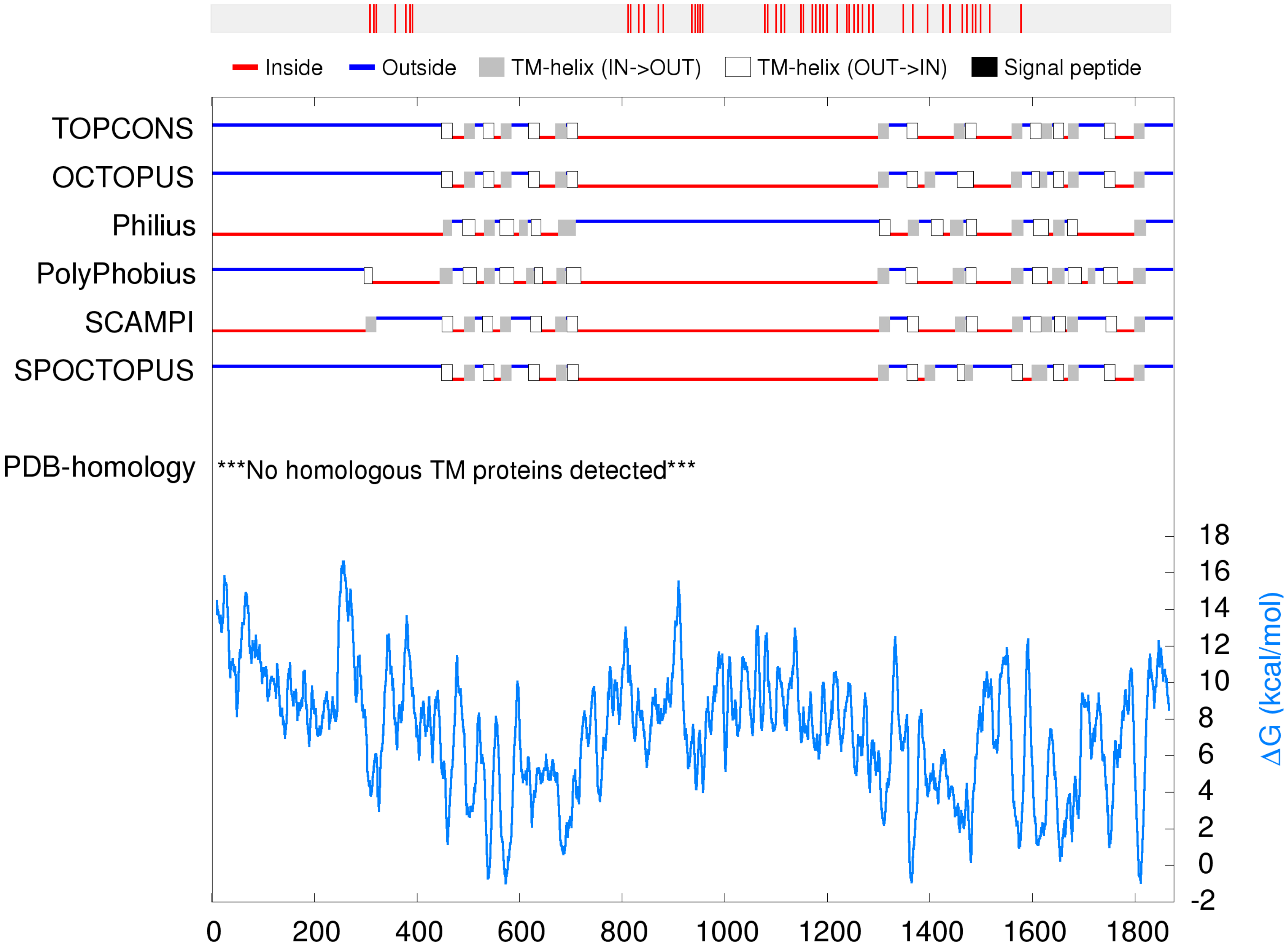
Inside
Outside
TM-helix (IN->OUT)
TM-helix (OUT->IN)
Signal peptide
TOPCONS
OCTOPUS
Philius
PolyPhobius
SCAMPI
SPOCTOPUS
PDB-homology
No homologous TM proteins detected
18
16
14
12
10
8
6
4
2
0
-2
ΔG (kcal/mol)
0
200
400
600
800
1000
1200
1400
1600
1800

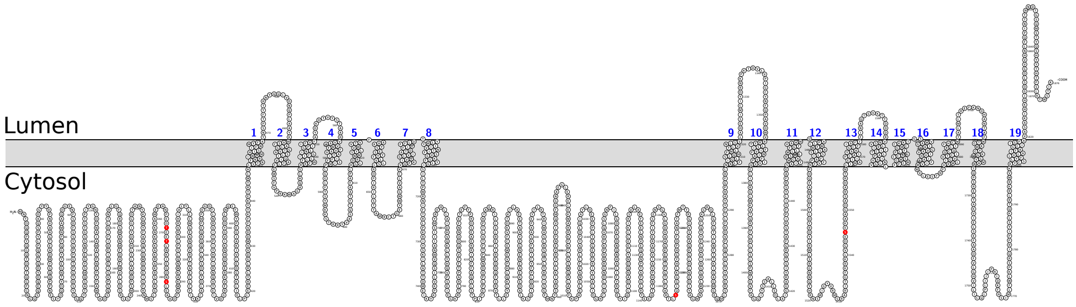
Lumen
Cytosol
1
2
3
4
5
6
7
8
9
10
11
12
13
14
15
16
17
18
19

Gpt2p

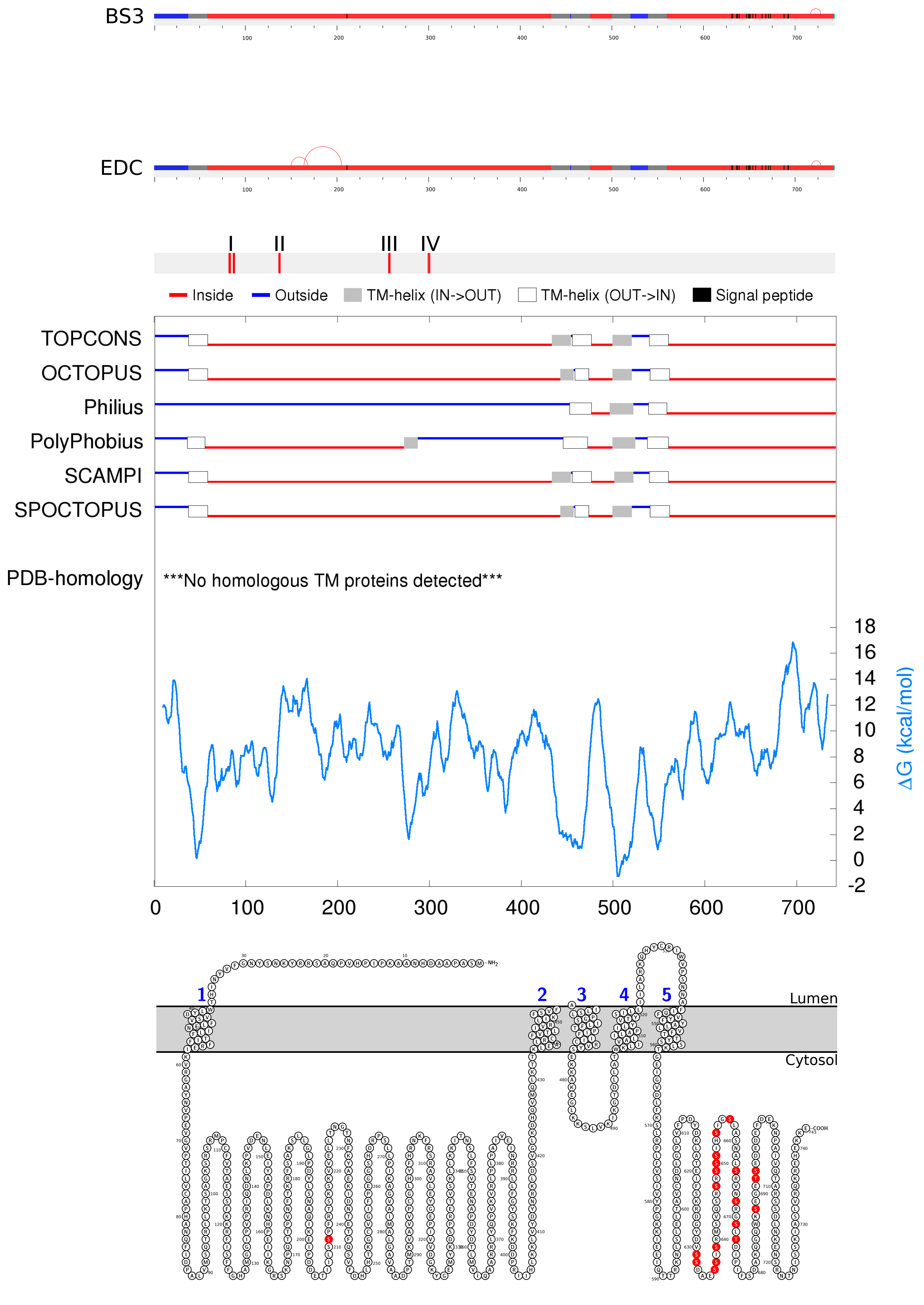
BS3
EDC
I
II
III
IV
Inside
Outside
TM-helix (IN->OUT)
TM-helix (OUT->IN)
Signal peptide
TOPCONS
OCTOPUS
Philius
PolyPhobius
SCAMPI
SPOCTOPUS
PDB-homology
No homologous TM proteins detected
ΔG (kcal/mol)
Lumen
Cytosol
NH2
COOH

Sct1p

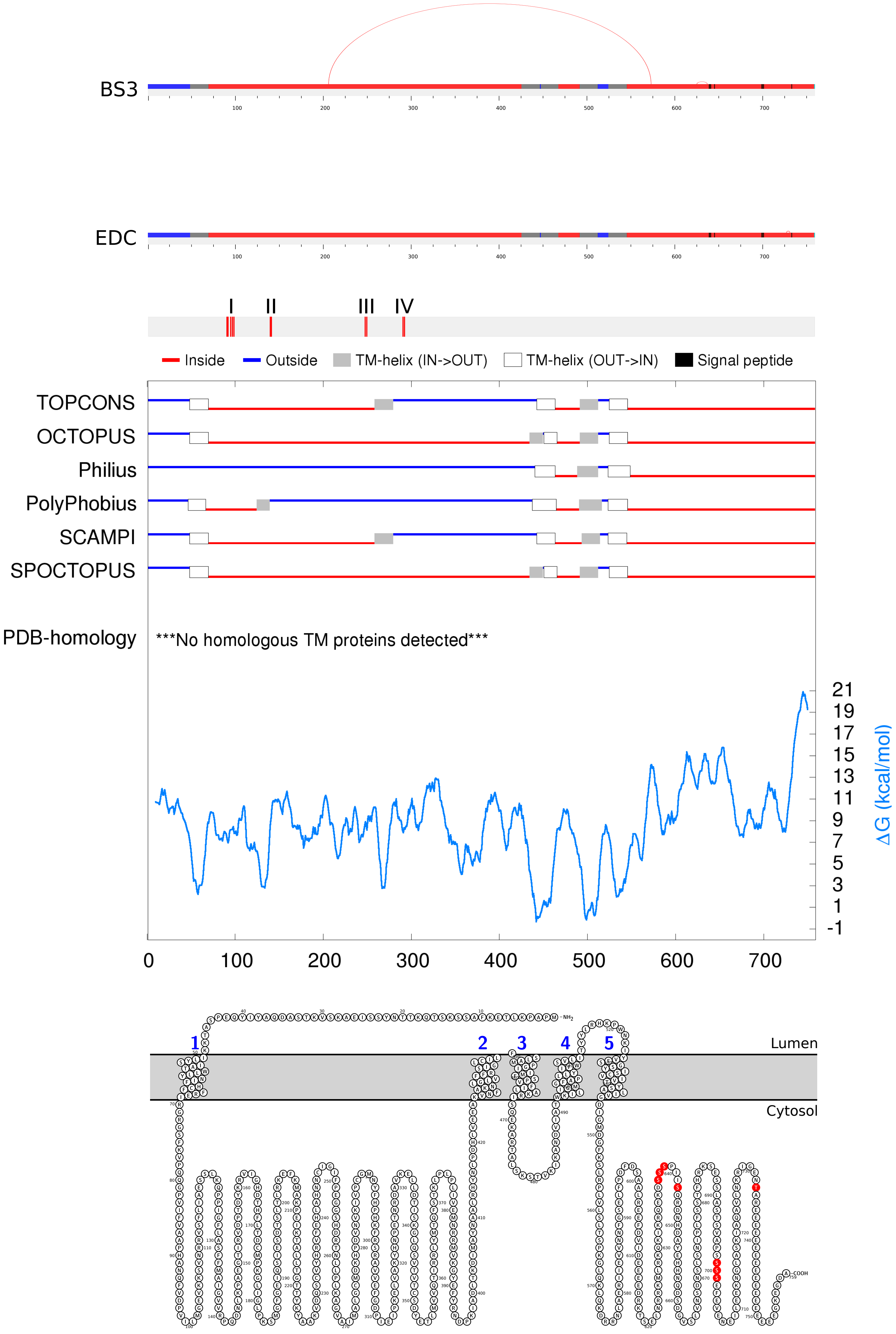

BS3
EDC
100 200 300 400 500 600 700
I II III IV
Inside
Outside
TM-helix (IN->OUT)
TM-helix (OUT->IN)
Signal peptide
TOPCONS
OCTOPUS
Philius
PolyPhobius
SCAMPI
SPOCTOPUS
PDB-homology
No homologous TM proteins detected
21 19 17 15 13 11 9 7 5 3 1 -1
ΔG (kcal/mol)
0 100 200 300 400 500 600 700
1 2 3 4 5
Lumen
Cytosol
-NH2
-COOH

Slc1p

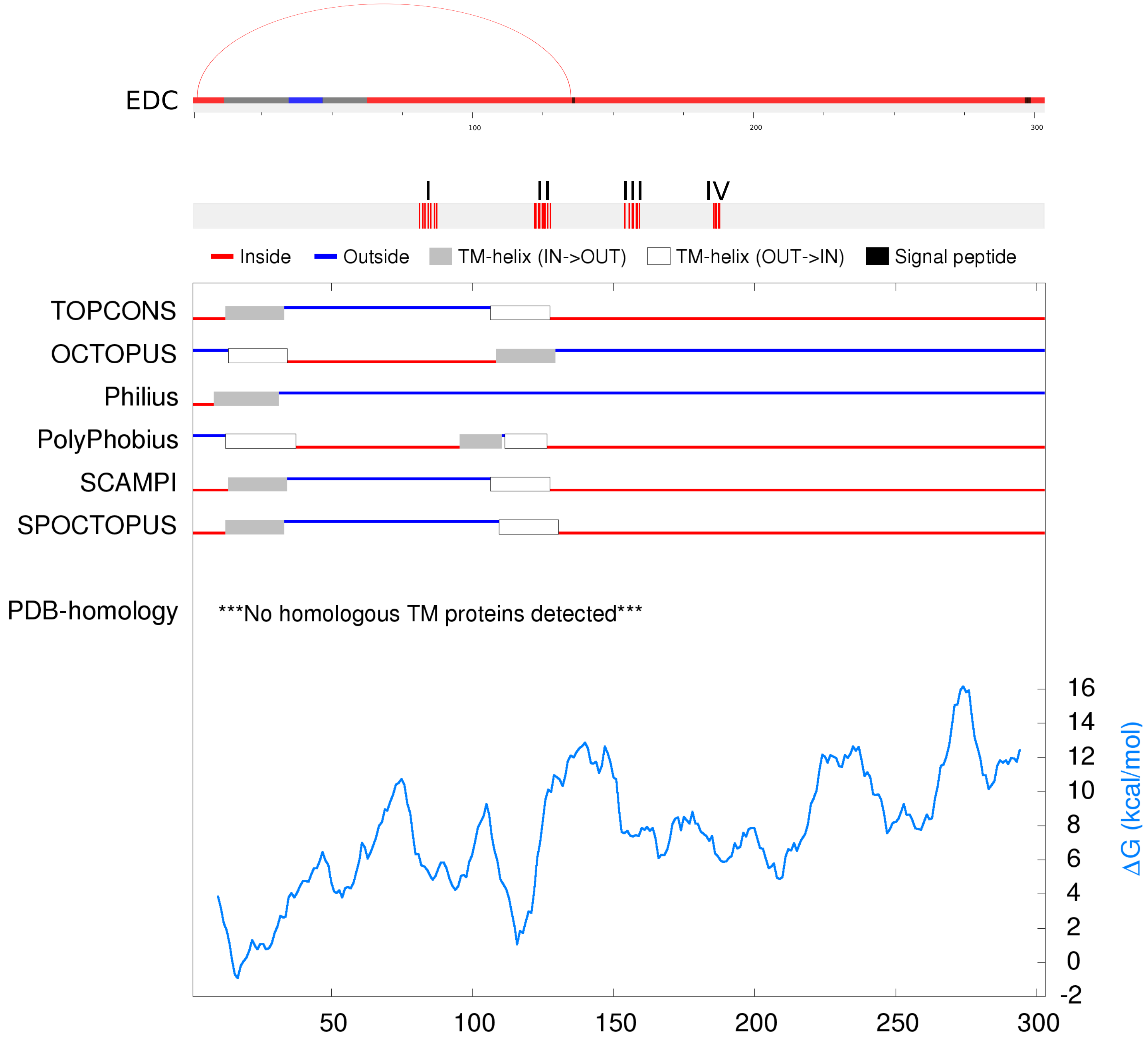
EDC
100
200
300
I
II
III
IV
Inside
Outside
TM-helix (IN->OUT)
TM-helix (OUT->IN)
Signal peptide
TOPCONS
OCTOPUS
Philius
PolyPhobius
SCAMPI
SPOCTOPUS
PDB-homology
No homologous TM proteins detected
16
14
12
10
8
6
4
2
0
-2
ΔG (kcal/mol)
50
100
150
200
250
300

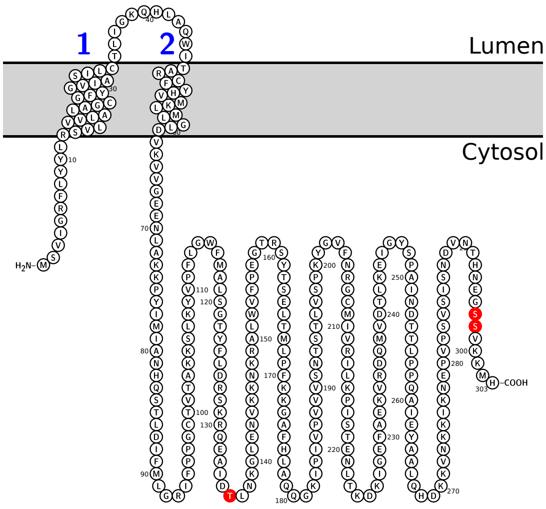
1
2
Lumen
Cytosol
H_2N-
-COOH

Spf1p

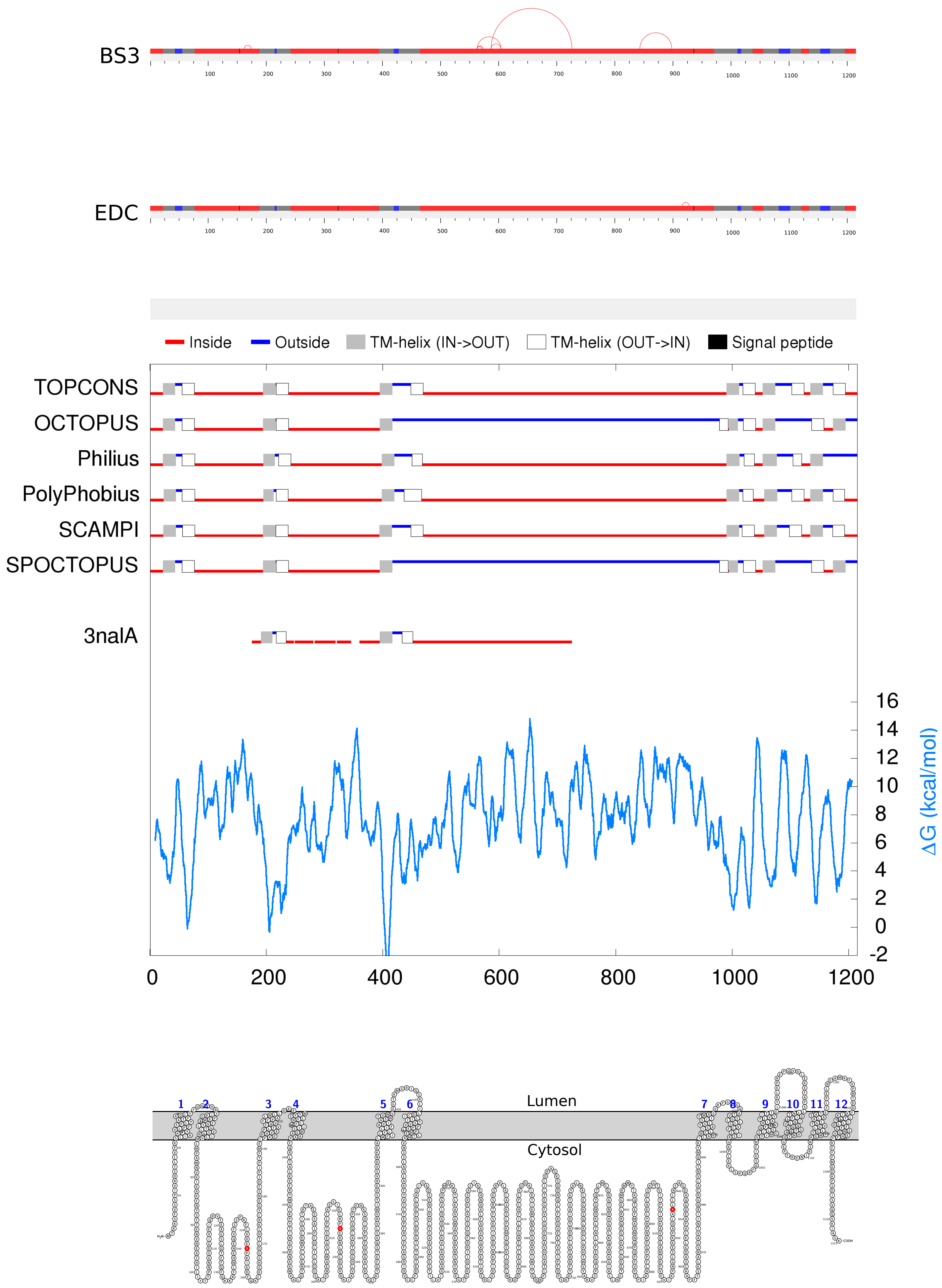

BS3
EDC
Inside
Outside
TM-helix (IN->OUT)
TM-helix (OUT->IN)
Signal peptide
TOPCONS
OCTOPUS
Philius
PolyPhobius
SCAMPI
SPOCTOPUS
3nalA
ΔG (kcal/mol)
16
14
12
10
8
6
4
2
0
-2
0
200
400
600
800
1000
1200
Lumen
Cytosol
1
2
3
4
5
6
7
8
9
10
11
12

Vph1p

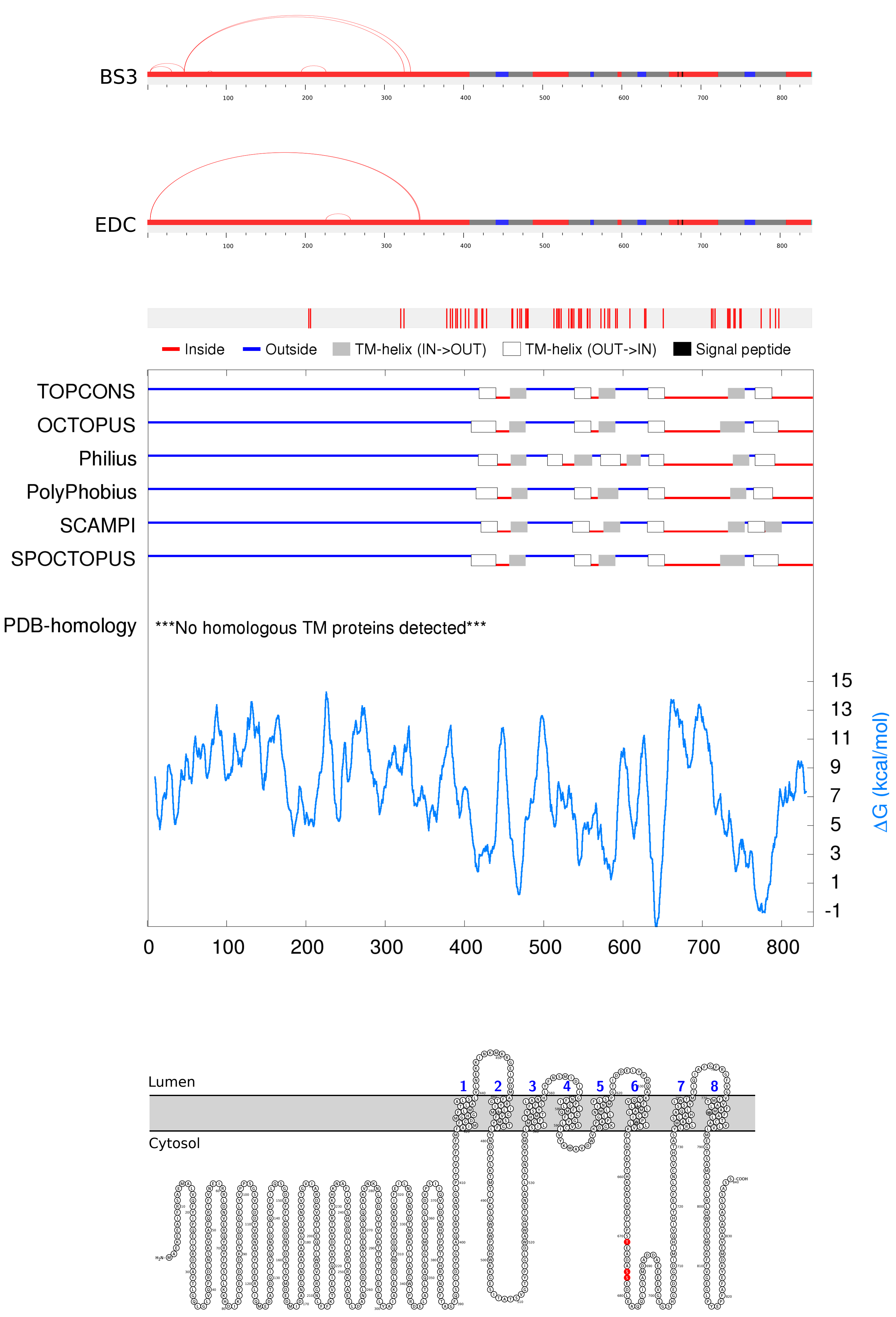
BS3
EDC
Inside
Outside
TM-helix (IN->OUT)
TM-helix (OUT->IN)
Signal peptide
TOPCONS
OCTOPUS
Philius
PolyPhobius
SCAMPI
SPOCTOPUS
PDB-homology
No homologous TM proteins detected
ΔG (kcal/mol)
15
13
11
9
7
5
3
1
-1
0
100
200
300
400
500
600
700
800
Lumen
Cytosol
1
2
3
4
5
6
7
8

Ybt1p

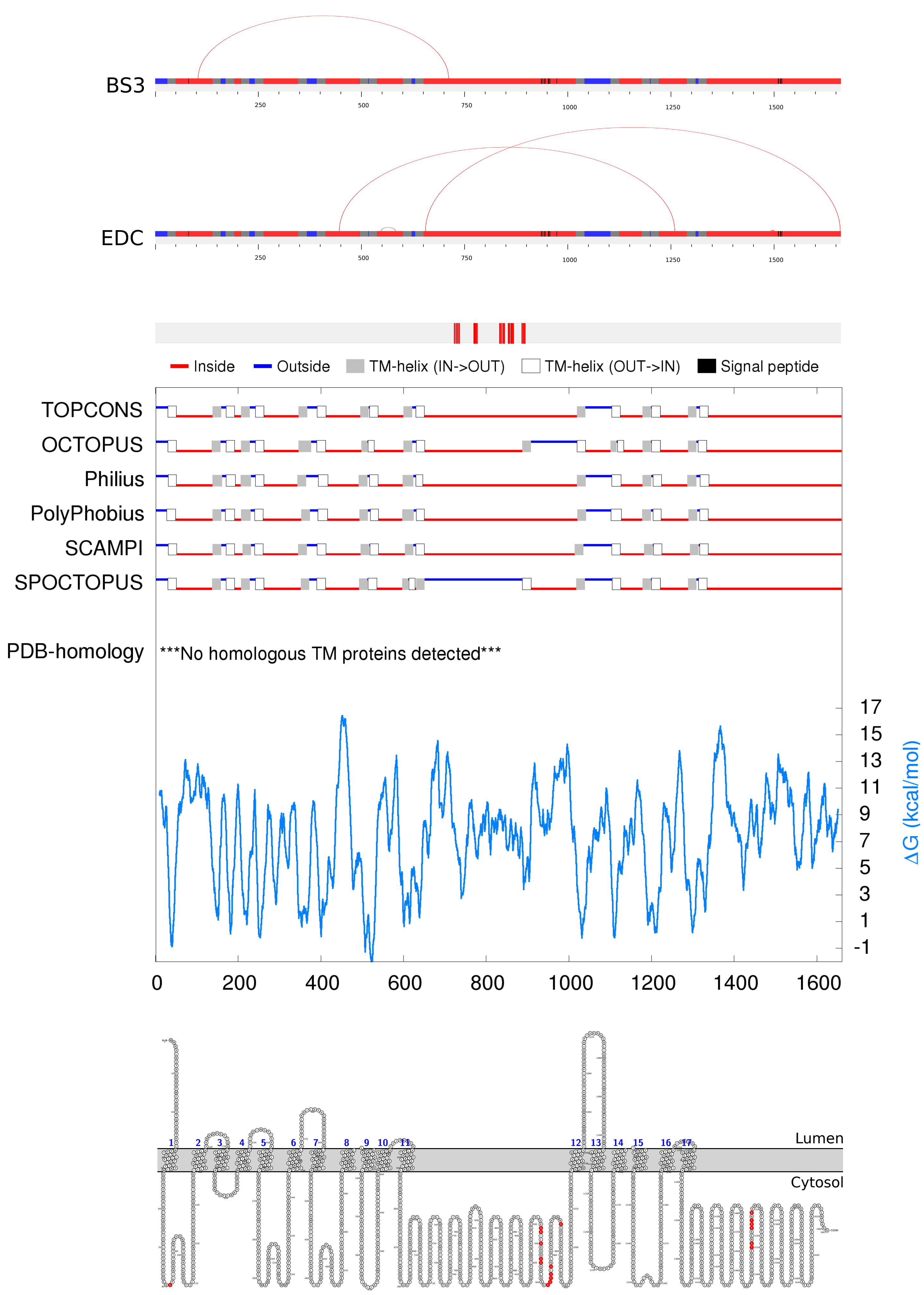

BS3
EDC
Inside
Outside
TM-helix (IN->OUT)
TM-helix (OUT->IN)
Signal peptide
TOPCONS
OCTOPUS
Philius
PolyPhobius
SCAMPI
SPOCTOPUS
PDB-homology
No homologous TM proteins detected
ΔG (kcal/mol)
Lumen
Cytosol
