# Supplementary material for: Chemical crosslinking and mass spectrometry to elucidate the topology of integral membrane proteins
Source: PLoS One. 2017 Oct 26;12(10):e0186840. doi: 10.1371/journal.pone.0186840 (PMC5658093; doi:10.1371/journal.pone.0186840)

S3 Fig. Putative topology of analyzed proteins

The central diagram (in each slide) shows transmembrane domains (TMDs) in grey, hairpin domains (HPD) in black, signal sequence (SS) in white, cytosolic and luminal loops of secretory MPS in red and blue, respectively, whereas mitochondrial intermembrane space and mitochondrial matrix loops are in green and pink, respectively. Phosphorylation sites are indicated by red balls connected by a line to the phosphosite and the orientation of the C termini as determined by {Kim et al., 2006, Proc Natl Acad Sci U S A, 103, 11142-7} are shown by a blue (cytosol) or green (ER lumen or extracellular) ball with the probability of the assignment given in %. TMD predictions shown with the following abbreviation: HMM (TMHMM; {Krogh et al., 2001, J Mol Biol, 305, 567-80}), MPEX (MPEx; {Snider et al., 2009, Protein Sci, 18, 2624-8}), OCTOPUS {Rath et al., 2013, BMC Bioinformatics, 14, 111}, Phyre2 {Kelley et al., 2015, Nat Protoc, 10, 845-58}, PP (PolyPhobius; {Käll et al., 2005, Bioinformatics, 21 Suppl 1, i251-7}), SCAMPI {Bernsel et al., 2008, Proc Natl Acad Sci U S A, 105, 7177-81}, Split {Juretić et al., 2002, J Chem Inf Comput Sci, 42, 620-32}, TC (TOPCONS; {Bernsel et al., 2009, Nucleic Acids Res, 37, W465-8}), UP (UniProt; {The UniProt Consortium, 2017, Nucleic Acids Res, 45, D158-D169}).

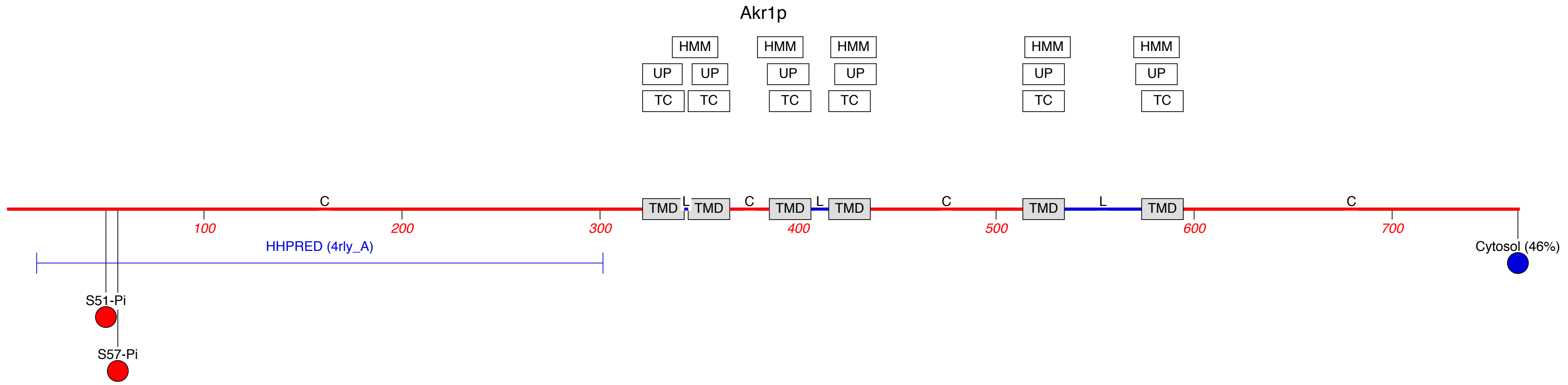

Akr1p
HMM
HMM
HMM
HMM
HMM
UP
UP
UP
UP
UP
UP
TC
TC
TC
TC
TC
TC
C
TMD
L
TMD
C
TMD
L
TMD
C
TMD
L
TMD
C
100
200
300
400
500
600
700
HHPRED (4rly_A)
Cytosol (46%)
S51-Pi
S57-Pi

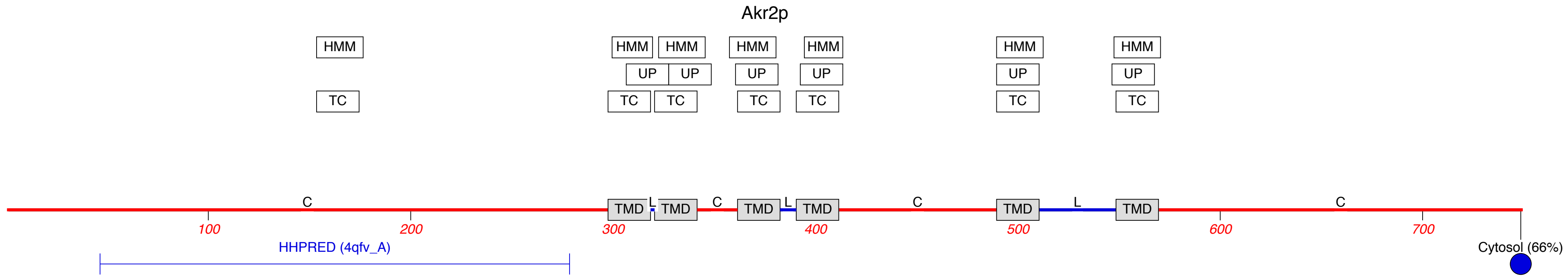

Akr2p
HMM
HMM
HMM
HMM
HMM
HMM
HMM
UP
UP
UP
UP
UP
UP
TC
TC
TC
TC
TC
TC
TC
C
TMD
L
TMD
C
TMD
L
TMD
C
TMD
L
TMD
C
100
200
300
400
500
600
700
HHPRED (4qfv_A)
Cytosol (66%)

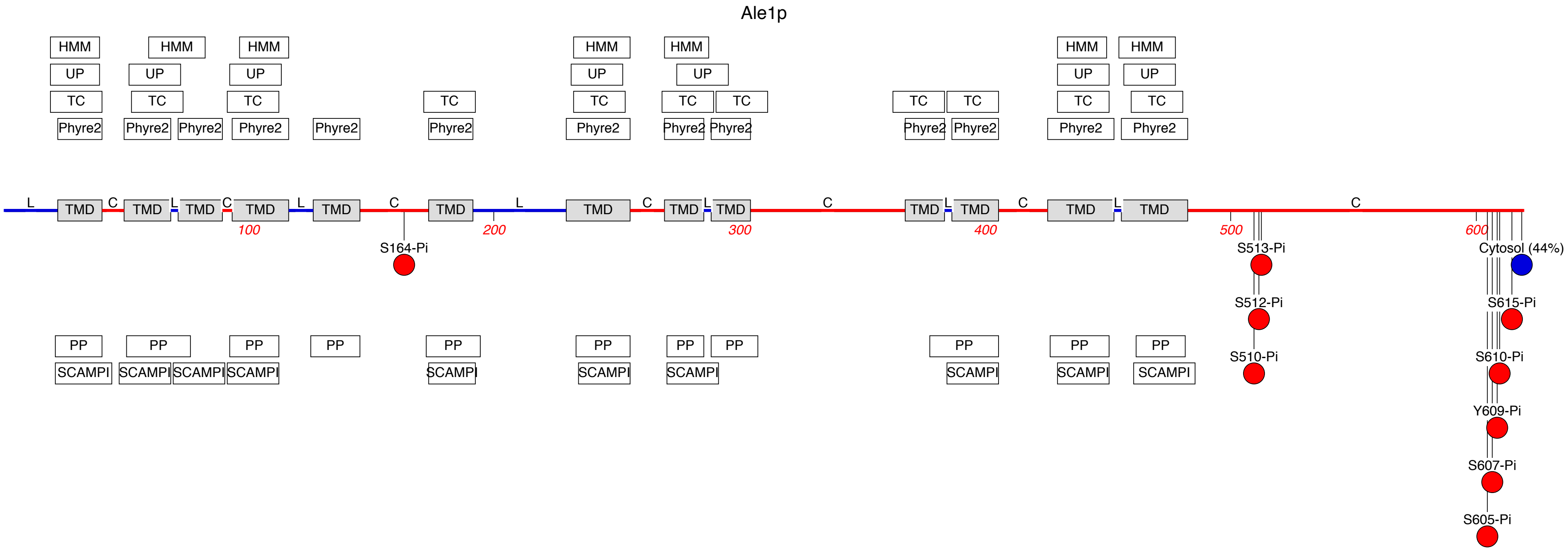
Ale1p
HMM
UP
TC
Phyre2
TMD
L
C
100
200
300
400
500
600
S164-Pi
S513-Pi
S512-Pi
S510-Pi
Cytosol (44%)
S615-Pi
S610-Pi
Y609-Pi
S607-Pi
S605-Pi
PP
SCAMPI

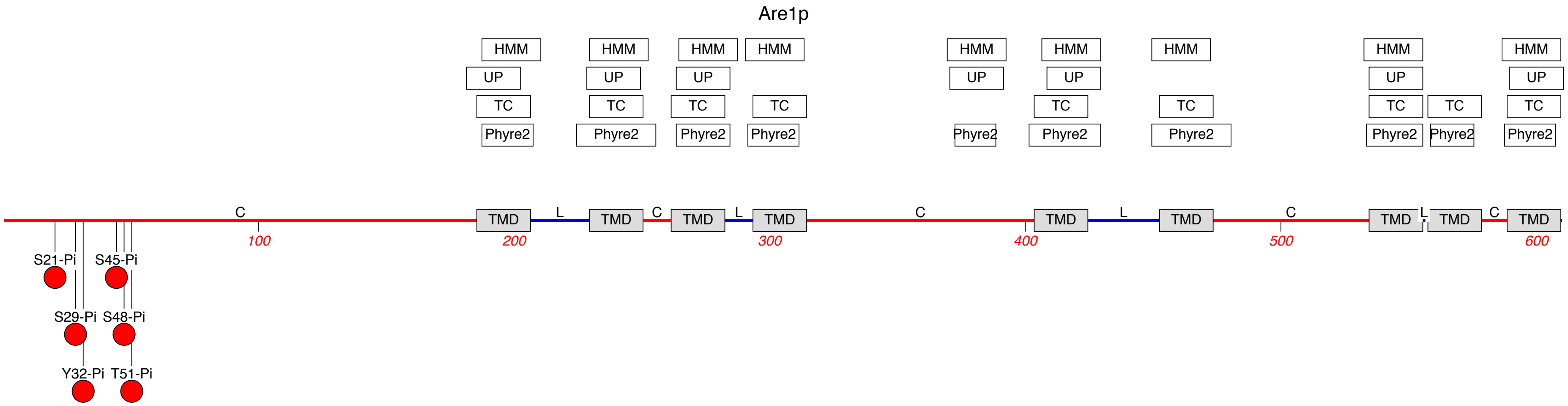
Are1p
HMM
UP
TC
Phyre2
TMD
L
C
S21-Pi
S29-Pi
Y32-Pi
S45-Pi
S48-Pi
T51-Pi
100
200
300
400
500
600

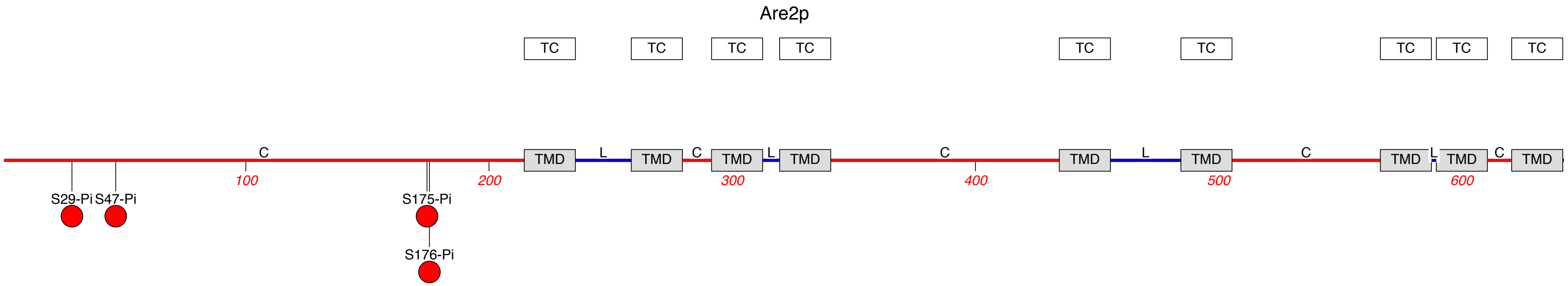
Are2p
TC
TC
TC
TC
TC
TC
TC
TC
TC
C
L
C
L
C
L
C
L
C
TMD
TMD
TMD
TMD
TMD
TMD
TMD
TMD
TMD
100
200
300
400
500
600
S29-Pi
S47-Pi
S175-Pi
S176-Pi

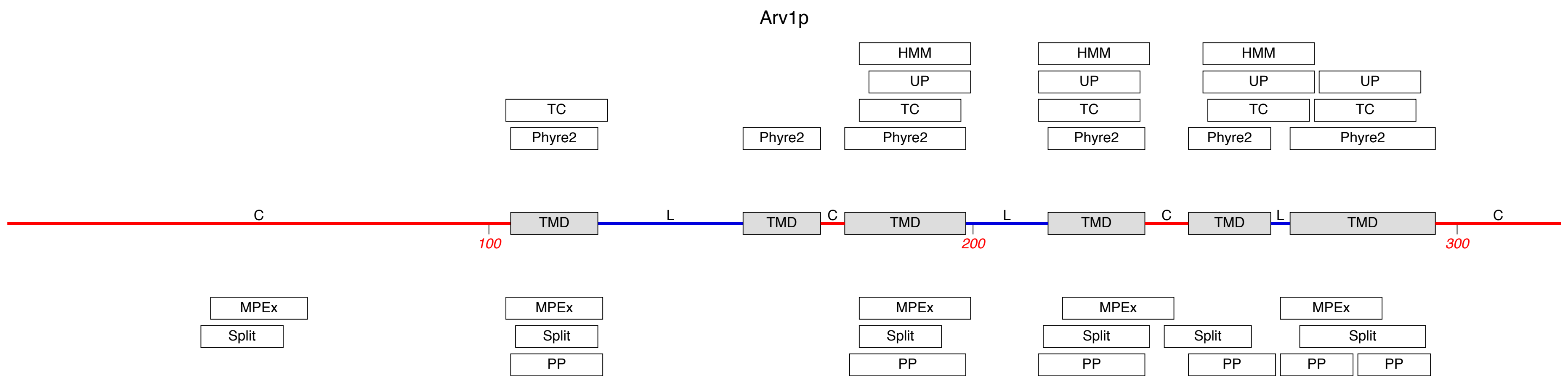

Arv1p
HMM
HMM
HMM
UP
UP
UP
UP
TC
TC
TC
TC
TC
Phyre2
Phyre2
Phyre2
Phyre2
Phyre2
Phyre2
C
TMD
L
TMD
C
TMD
L
TMD
C
TMD
L
TMD
C
100
200
300
MPEx
MPEx
MPEx
MPEx
MPEx
Split
Split
Split
Split
Split
Split
PP
PP
PP
PP
PP
PP

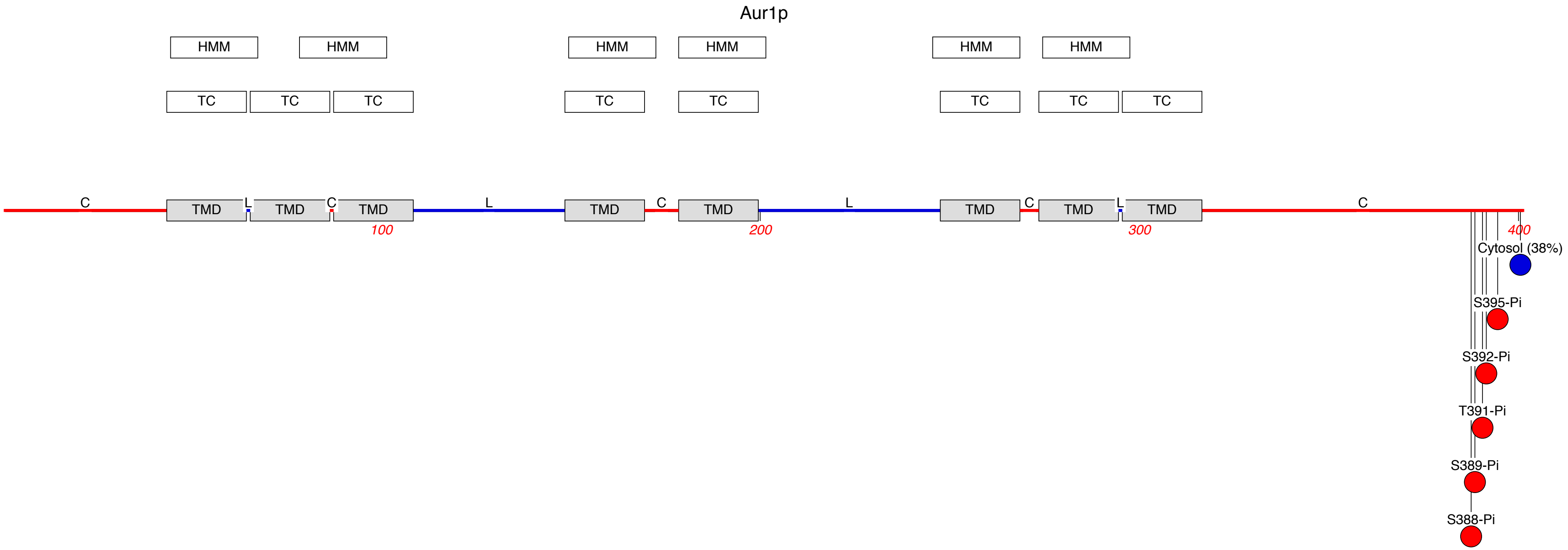
Aur1p
HMM
HMM
HMM
HMM
HMM
HMM
TC
TC
TC
TC
TC
TC
TC
TC
C
TMD
L
TMD
C
TMD
L
TMD
C
TMD
L
TMD
C
TMD
L
TMD
C
100
200
300
400
Cytosol (38%)
S395-Pi
S392-Pi
T391-Pi
S389-Pi
S388-Pi

Cds1p

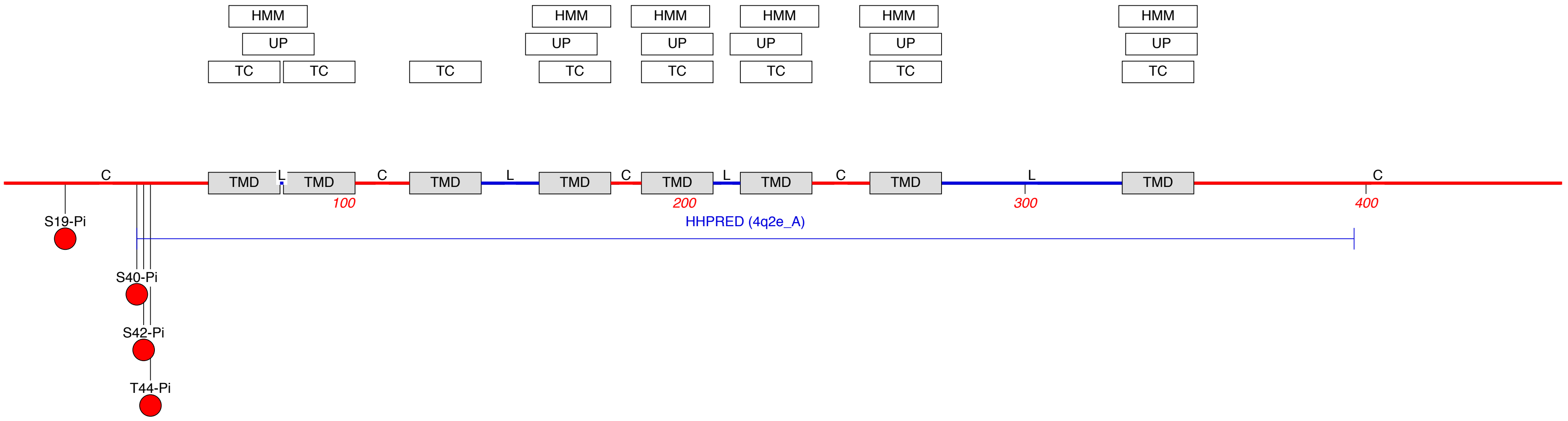

HMM
UP
TC
TMD
L
C
100
200
300
400
HHPRED (4q2e_A)
S19-Pi
S40-Pi
S42-Pi
T44-Pi

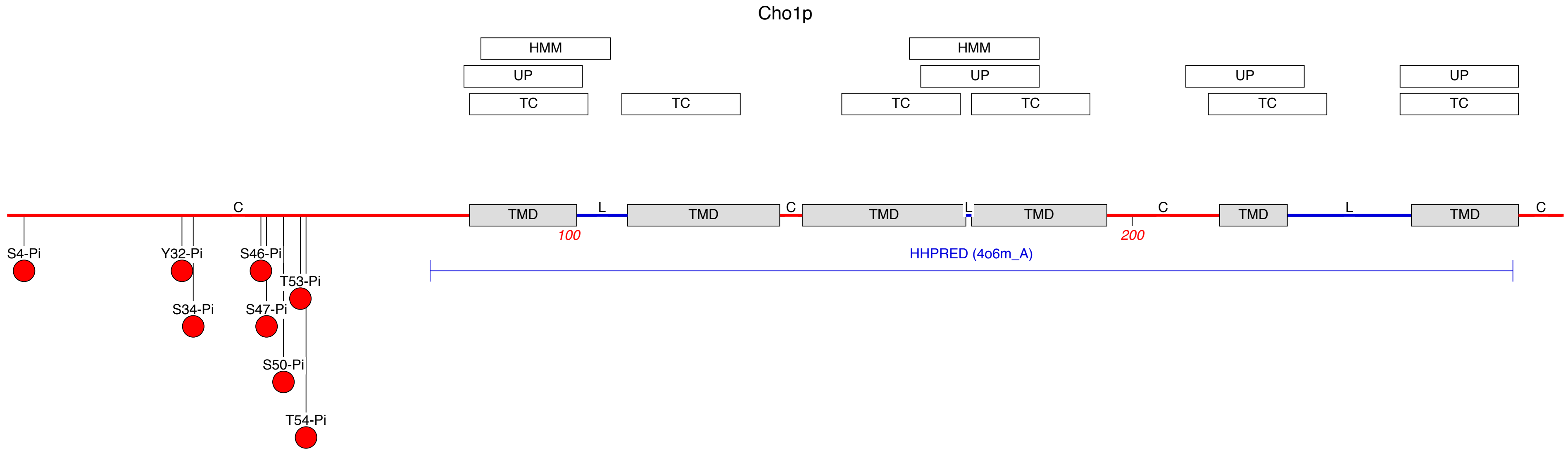
Cho1p
HMM
HMM
UP
UP
UP
UP
TC
TC
TC
TC
TC
TC
C
TMD
L
TMD
C
TMD
L
TMD
C
TMD
L
TMD
C
100
200
HHPRED (4o6m_A)
S4-Pi
Y32-Pi
S34-Pi
S46-Pi
S47-Pi
S50-Pi
T53-Pi
T54-Pi

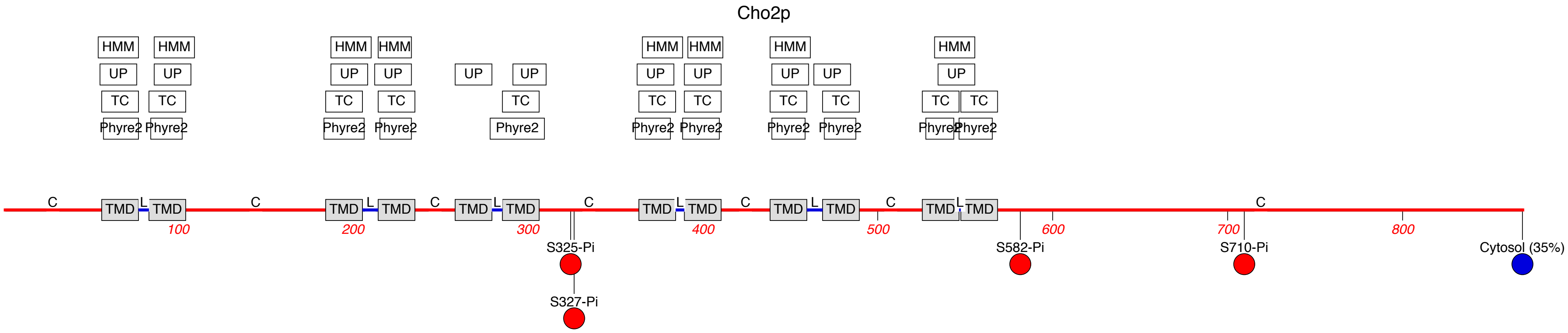
Cho2p
HMM
UP
TC
Phyre2
TMD
L
C
100
200
300
400
500
600
700
800
S325-Pi
S327-Pi
S582-Pi
S710-Pi
Cytosol (35%)

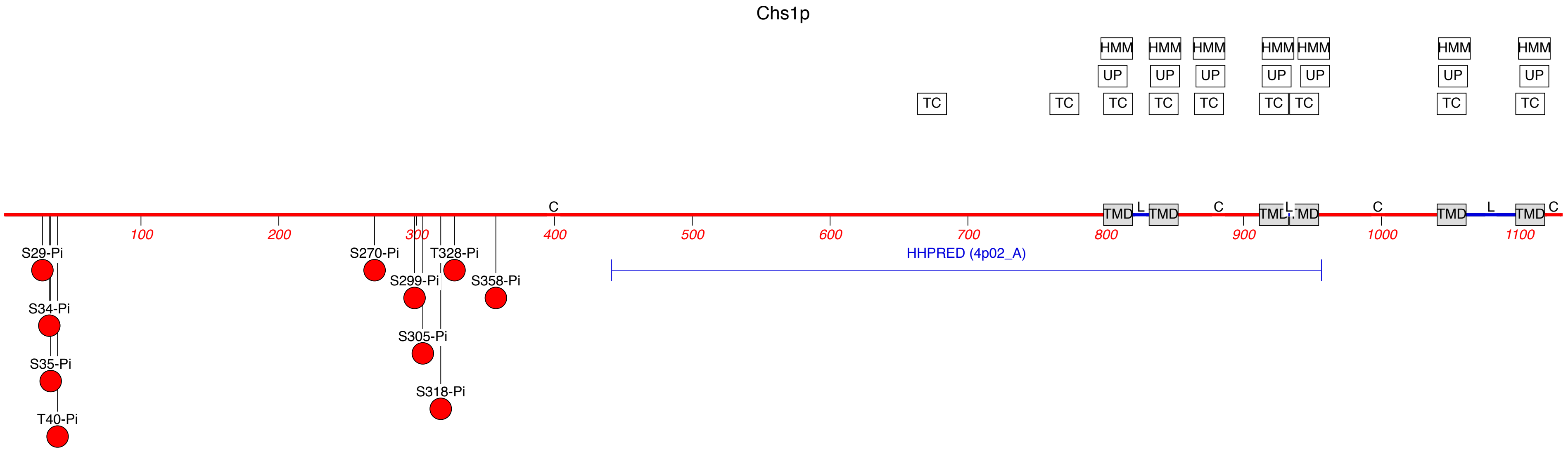

Chs1p
HMM
UP
TC
TMD
L
C
100
200
300
400
500
600
700
800
900
1000
1100
HHPRED (4p02_A)
S29-Pi
S34-Pi
S35-Pi
T40-Pi
S270-Pi
S299-Pi
S305-Pi
S318-Pi
T328-Pi
S358-Pi

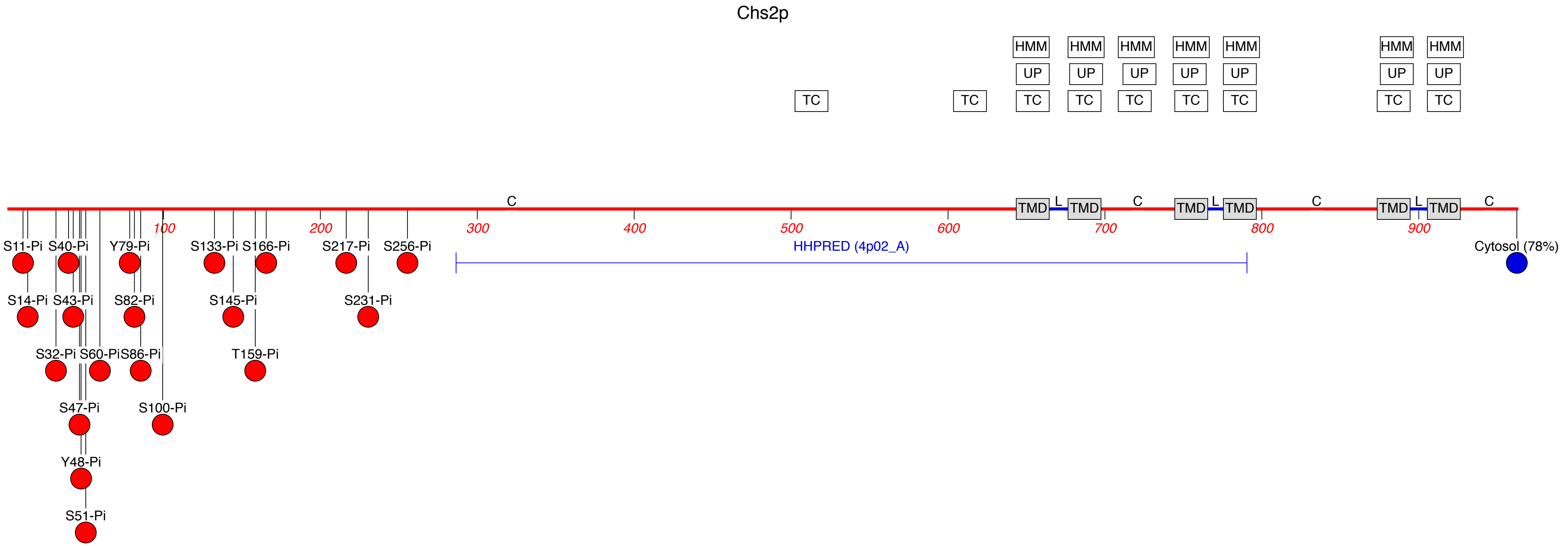

Chs2p
HMM HMM HMM HMM HMM HMM HMM
UP UP UP UP UP UP UP
TC TC TC TC TC TC TC TC TC
TMD L TMD C TMD L TMD C TMD L TMD C
C
100 200 300 400 500 600 700 800 900
S11-Pi S40-Pi Y79-Pi S133-Pi S166-Pi S217-Pi S256-Pi
S14-Pi S43-Pi S82-Pi S145-Pi S231-Pi
S32-Pi S60-Pi S86-Pi T159-Pi
S47-Pi S100-Pi
Y48-Pi
S51-Pi
HHPRED (4p02_A)
Cytosol (78%)

Chs3p

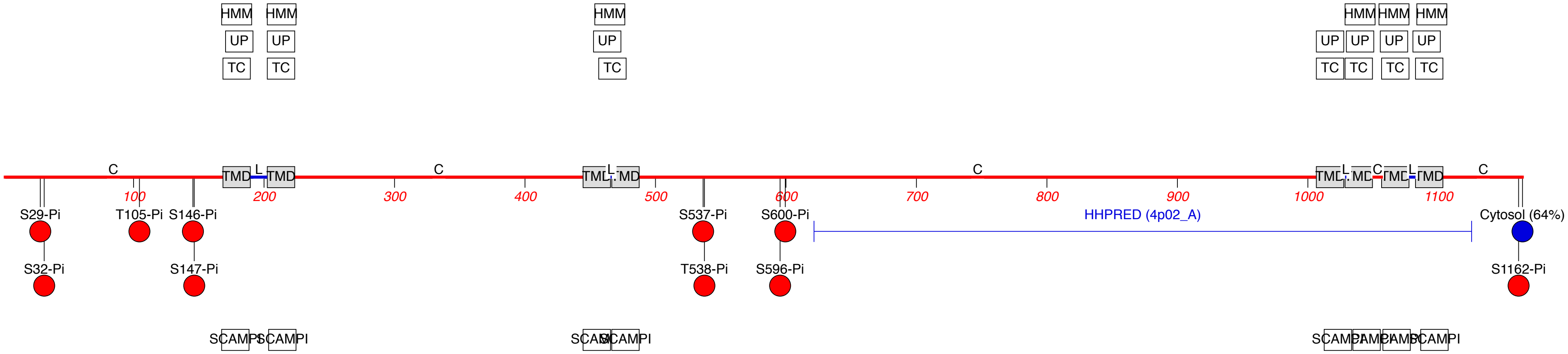
HMM
UP
TC
TMD
L
C
100
200
300
400
500
600
700
800
900
1000
1100
S29-Pi
S32-Pi
T105-Pi
S146-Pi
S147-Pi
S537-Pi
T538-Pi
S600-Pi
S596-Pi
HHPRED (4p02_A)
Cytosol (64%)
S1162-Pi
SCAMPI

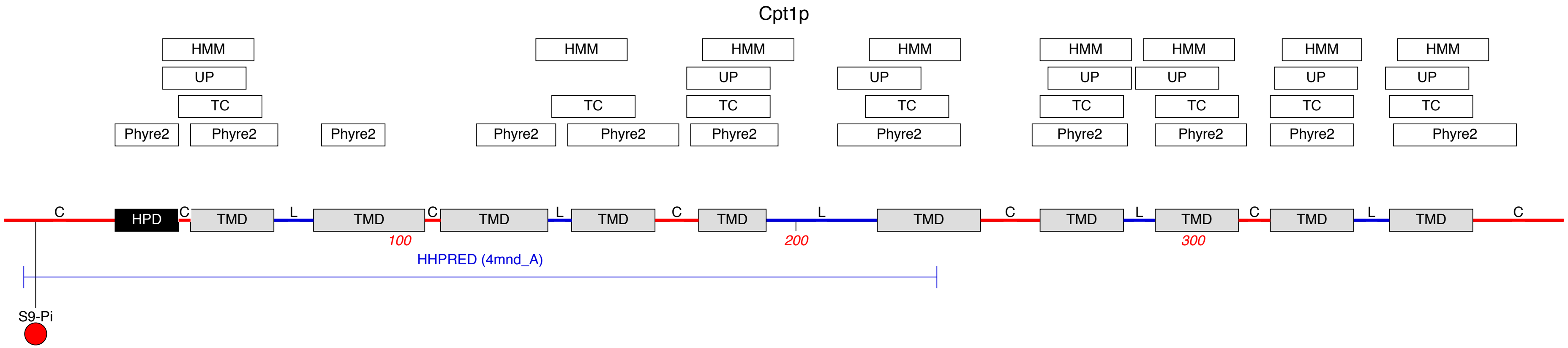

Cpt1p
HMM
HMM
HMM
HMM
HMM
HMM
HMM
HMM
UP
UP
UP
UP
UP
UP
UP
TC
TC
TC
TC
TC
TC
TC
TC
Phyre2
Phyre2
Phyre2
Phyre2
Phyre2
Phyre2
Phyre2
Phyre2
Phyre2
Phyre2
Phyre2
C
HPD
C
TMD
L
TMD
C
TMD
L
TMD
C
TMD
L
TMD
C
TMD
L
TMD
C
TMD
L
TMD
C
100
200
300
HHPRED (4mnd_A)
S9-Pi

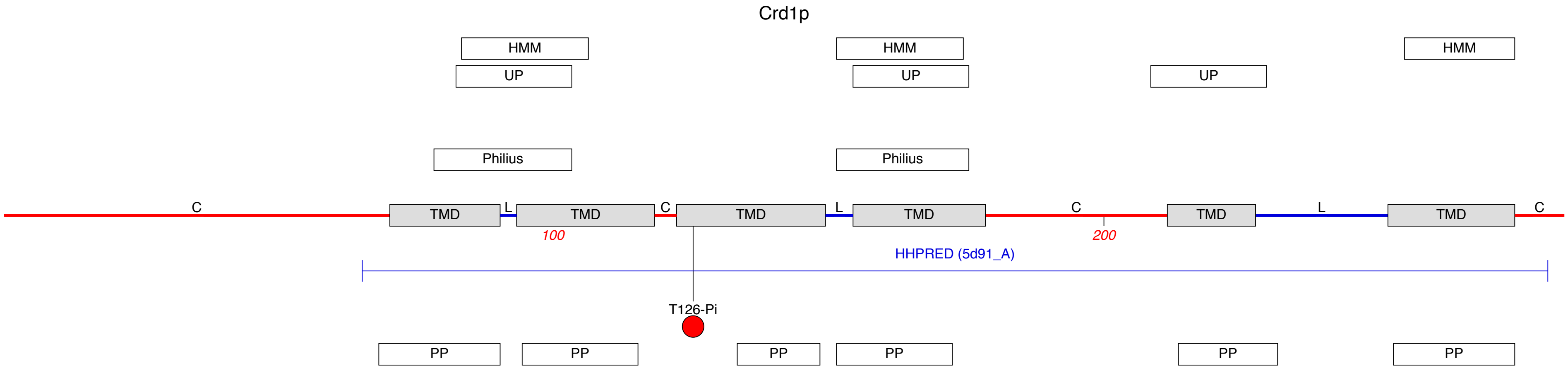

Crd1p
HMM
HMM
HMM
UP
UP
UP
Philius
Philius
C
TMD
L
TMD
C
TMD
L
TMD
C
TMD
L
TMD
C
100
200
HHPRED (5d91_A)
T126-Pi
PP
PP
PP
PP
PP
PP

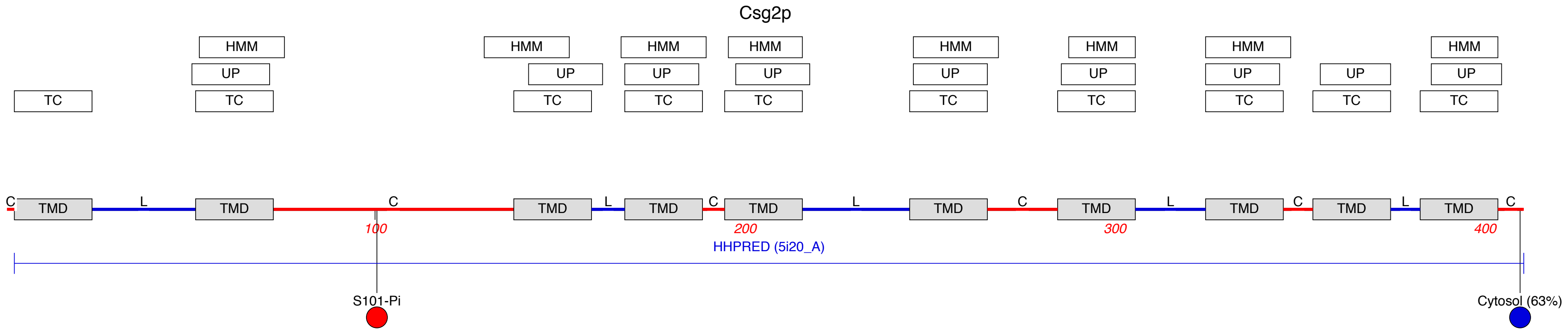
Csg2p
HMM
UP
TC
TMD
C
L
100
200
300
400
HHPRED (5i20_A)
S101-Pi
Cytosol (63%)

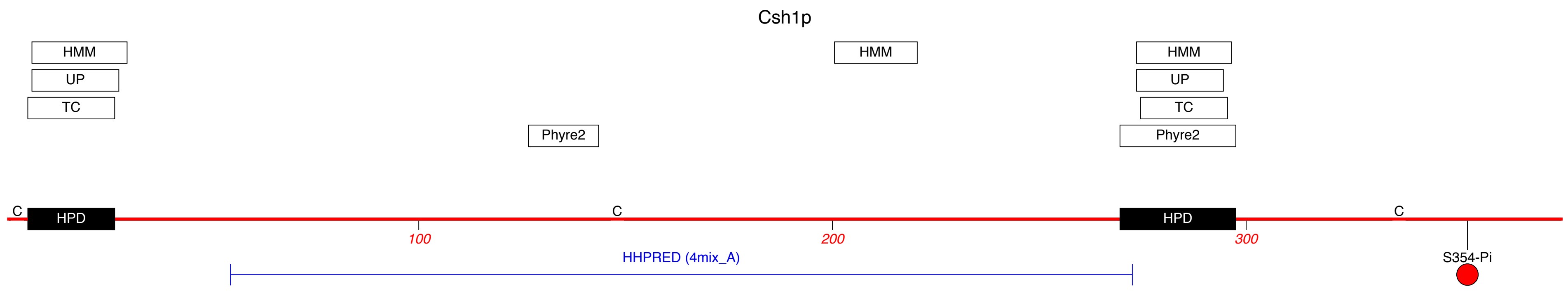
Csh1p
HMM
UP
TC
Phyre2
HMM
HMM
UP
TC
Phyre2
C
HPD
C
HPD
C
100
200
300
HHPRED (4mix_A)
S354-Pi

Cst26p

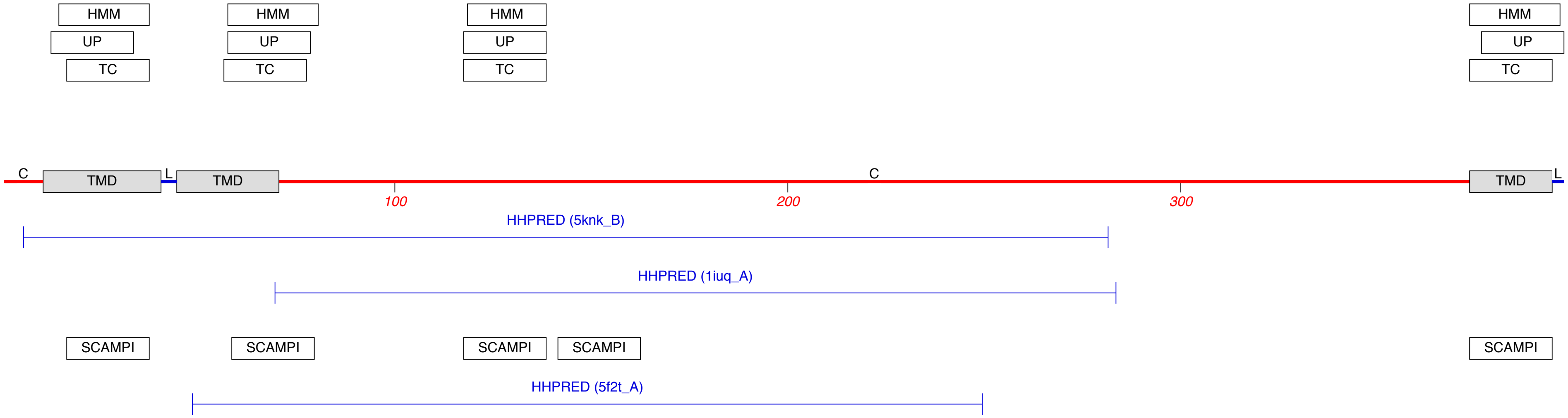
HMM
UP
TC
HMM
UP
TC
HMM
UP
TC
HMM
UP
TC
C
TMD
L
TMD
C
TMD
L
100
200
300
HHPRED (5knk_B)
HHPRED (1iuq_A)
SCAMPI
SCAMPI
SCAMPI
SCAMPI
SCAMPI
HHPRED (5f2t_A)

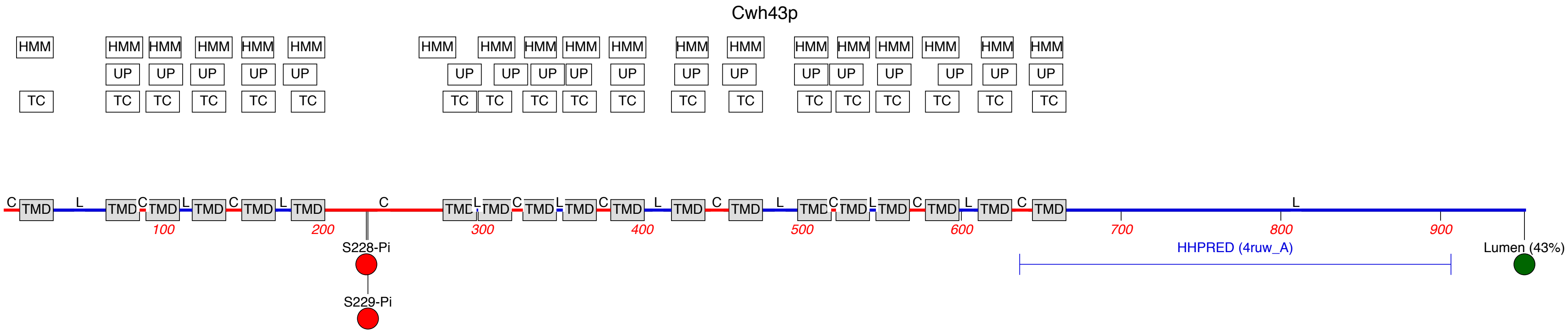
Cwh43p
HMM
UP
TC
TMD
C
L
100
200
300
400
500
600
700
800
900
S228-Pi
S229-Pi
HHPRED (4ruw_A)
Lumen (43%)

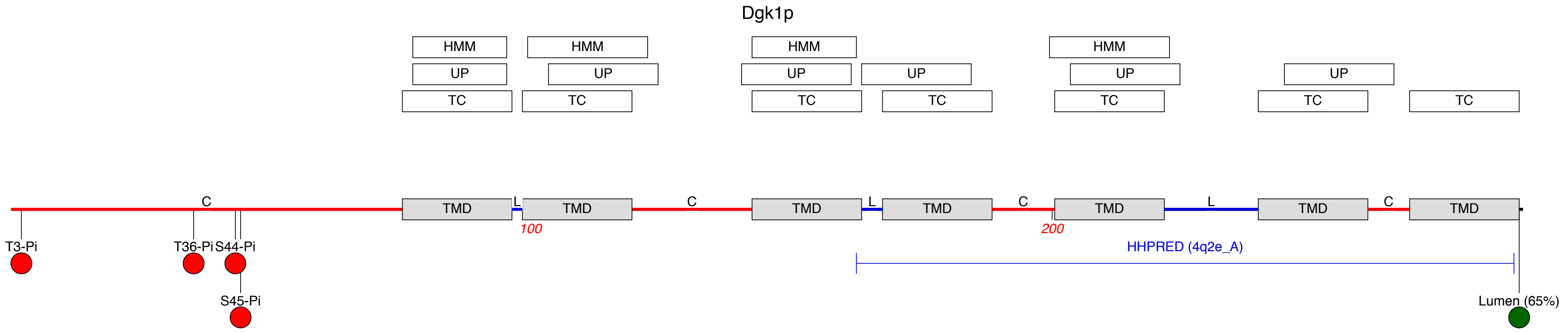

Dgk1p
HMM
HMM
HMM
HMM
UP
UP
UP
UP
UP
UP
TC
TC
TC
TC
TC
TC
TC
C
TMD
L
TMD
C
TMD
L
TMD
C
TMD
L
TMD
C
TMD
100
200
T3-Pi
T36-Pi
S44-Pi
S45-Pi
HHPRED (4q2e_A)
Lumen (65%)

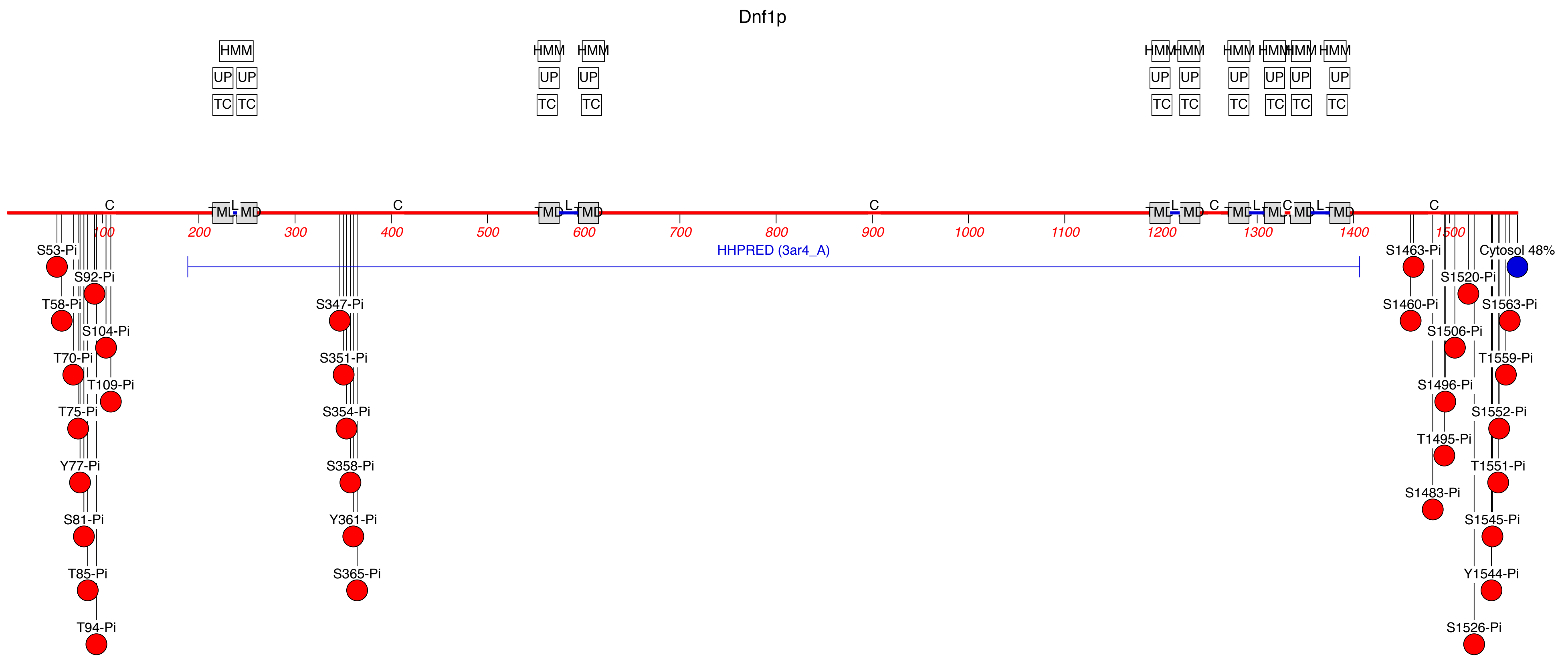
Dnf1p
HMM
UP
TC
TMD
L
C
100
200
300
400
500
600
700
800
900
1000
1100
1200
1300
1400
1500
HHPRED (3ar4_A)
Cytosol 48%
S53-Pi
S92-Pi
T58-Pi
S104-Pi
T70-Pi
T109-Pi
T75-Pi
Y77-Pi
S81-Pi
T85-Pi
T94-Pi
S347-Pi
S351-Pi
S354-Pi
S358-Pi
Y361-Pi
S365-Pi
S1463-Pi
S1520-Pi
S1460-Pi
S1563-Pi
S1506-Pi
T1559-Pi
S1496-Pi
S1552-Pi
T1495-Pi
T1551-Pi
S1483-Pi
S1545-Pi
Y1544-Pi
S1526-Pi

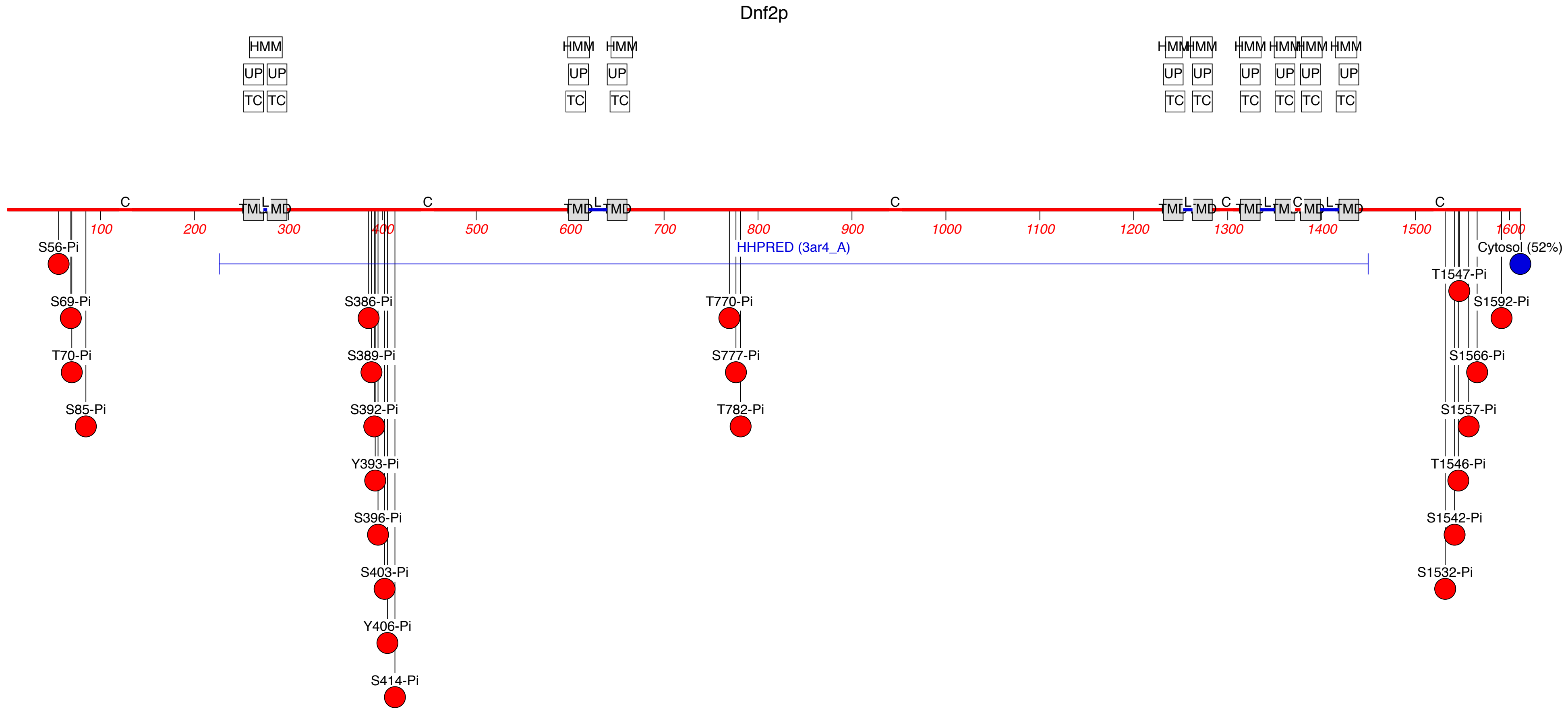

Dnf2p
HMM
UP
TC
C
L
TMD
100
200
300
400
500
600
700
800
900
1000
1100
1200
1300
1400
1500
1600
HHPRED (3ar4_A)
Cytosol (52%)
S56-Pi
S69-Pi
T70-Pi
S85-Pi
S386-Pi
S389-Pi
S392-Pi
Y393-Pi
S396-Pi
S403-Pi
Y406-Pi
S414-Pi
T770-Pi
S777-Pi
T782-Pi
T1547-Pi
S1592-Pi
S1566-Pi
S1557-Pi
T1546-Pi
S1542-Pi
S1532-Pi

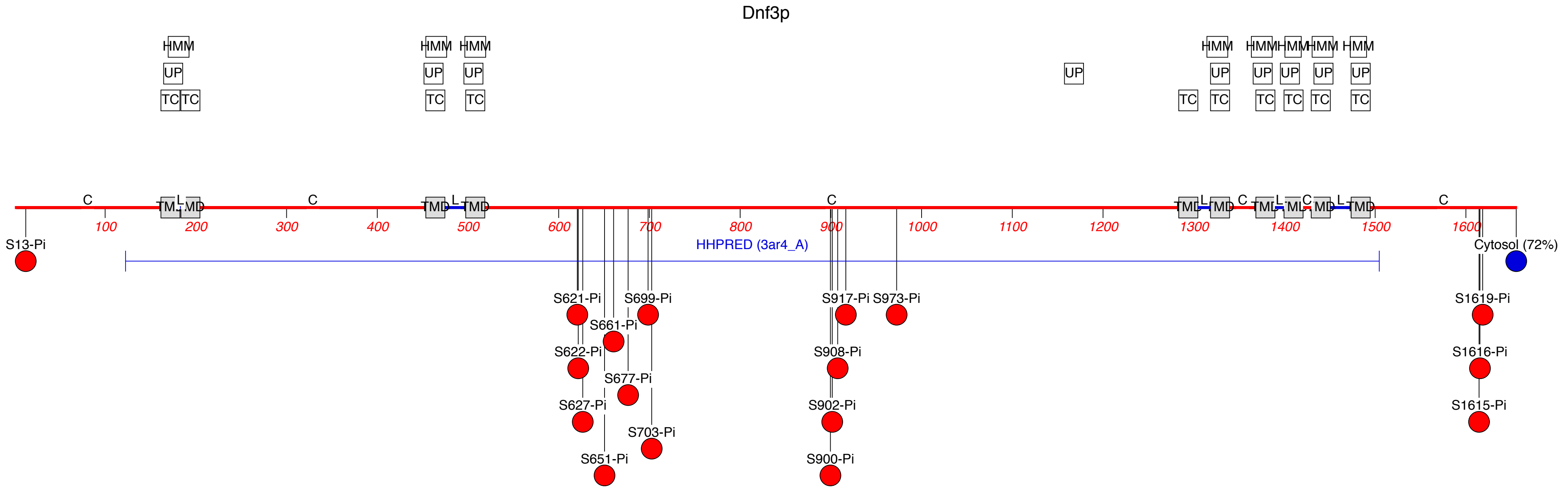
Dnf3p
HMM
UP
TC
TMD
L
C
100
200
300
400
500
600
700
800
900
1000
1100
1200
1300
1400
1500
1600
S13-Pi
HHPRED (3ar4_A)
Cytosol (72%)
S621-Pi
S699-Pi
S661-Pi
S622-Pi
S677-Pi
S627-Pi
S703-Pi
S651-Pi
S917-Pi
S973-Pi
S908-Pi
S902-Pi
S900-Pi
S1619-Pi
S1616-Pi
S1615-Pi

Dpp1p

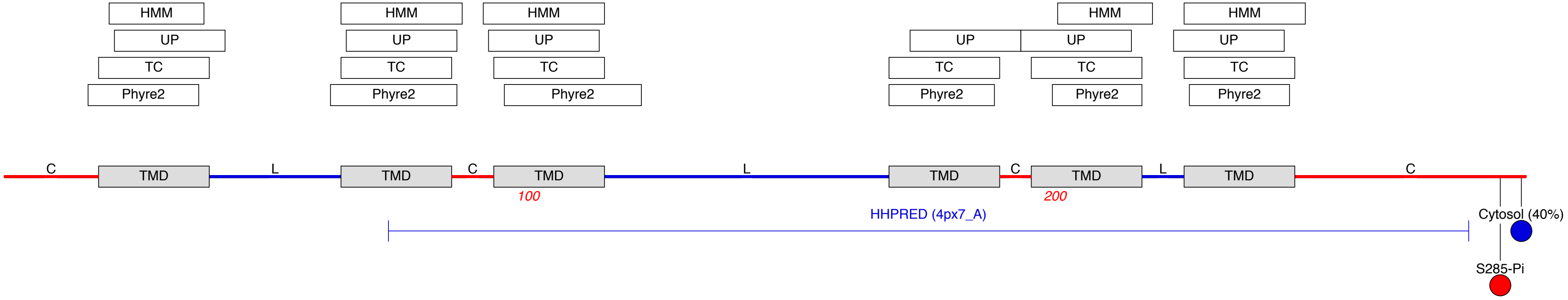
HMM
UP
TC
Phyre2
C
TMD
L
100
200
HHPRED (4px7_A)
Cytosol (40%)
S285-Pi

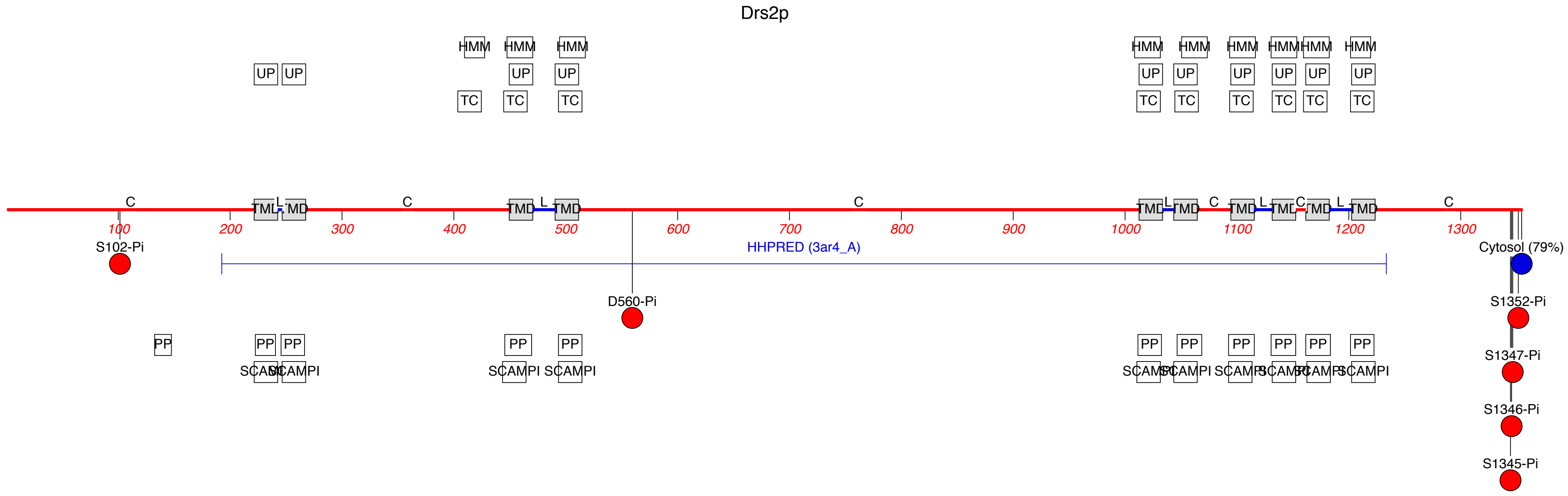
Drs2p
HMM
UP
TC
C
TMD
L
100
200
300
400
500
600
700
800
900
1000
1100
1200
1300
S102-Pi
HHPRED (3ar4_A)
Cytosol (79%)
D560-Pi
S1352-Pi
S1347-Pi
S1346-Pi
S1345-Pi
PP
SCAMPI

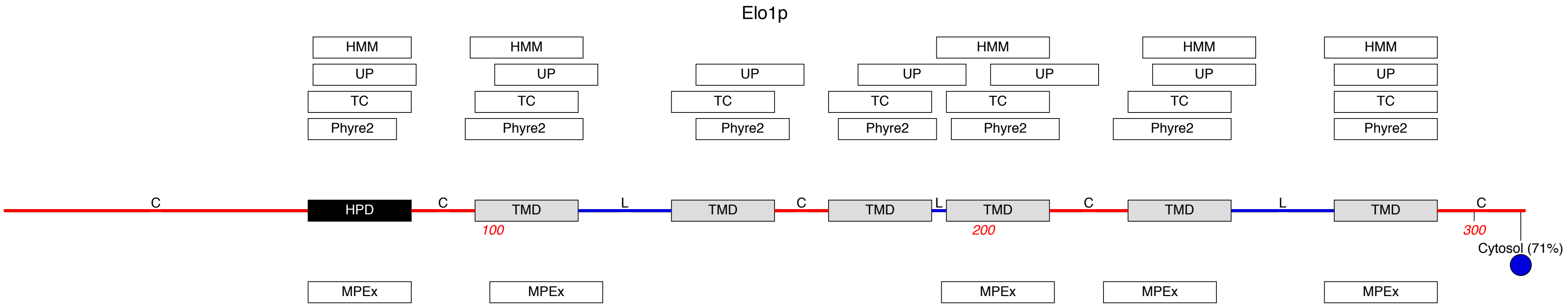
Elo1p
HMM
HMM
HMM
HMM
HMM
UP
UP
UP
UP
UP
UP
UP
TC
TC
TC
TC
TC
TC
TC
Phyre2
Phyre2
Phyre2
Phyre2
Phyre2
Phyre2
Phyre2
C
HPD
C
TMD
L
TMD
C
TMD
L
TMD
C
TMD
L
TMD
C
100
200
300
Cytosol (71%)
MPEx
MPEx
MPEx
MPEx
MPEx

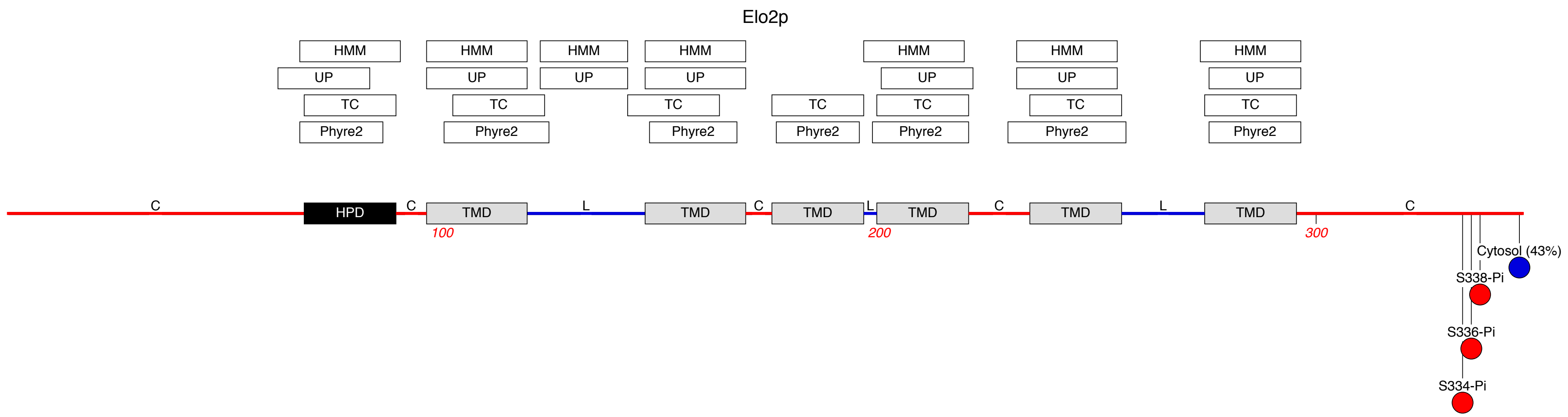

Elo2p
HMM
UP
TC
Phyre2
C
HPD
C
TMD
L
TMD
C
TMD
L
TMD
C
TMD
L
TMD
C
100
200
300
Cytosol (43%)
S338-Pi
S336-Pi
S334-Pi

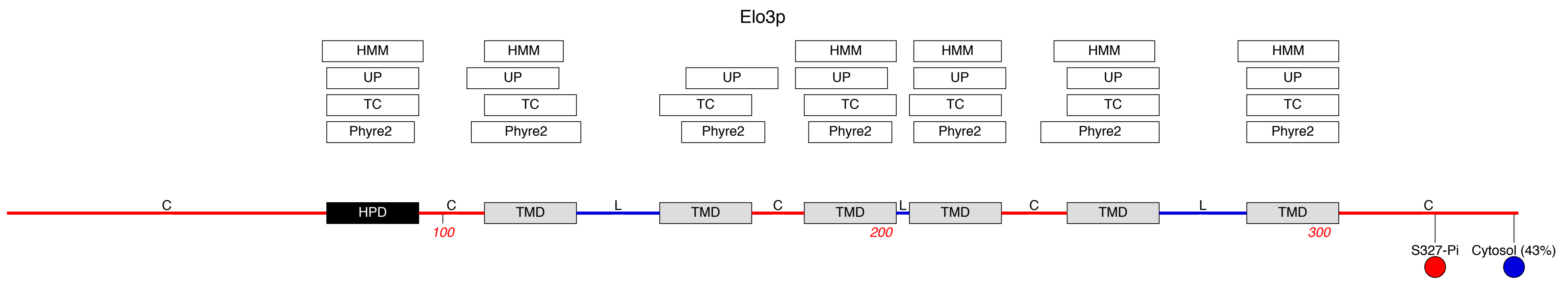

Elo3p
HMM
UP
TC
Phyre2
C
HPD
C
TMD
L
TMD
C
TMD
L
TMD
C
TMD
L
TMD
C
100
200
300
S327-Pi
Cytosol (43%)

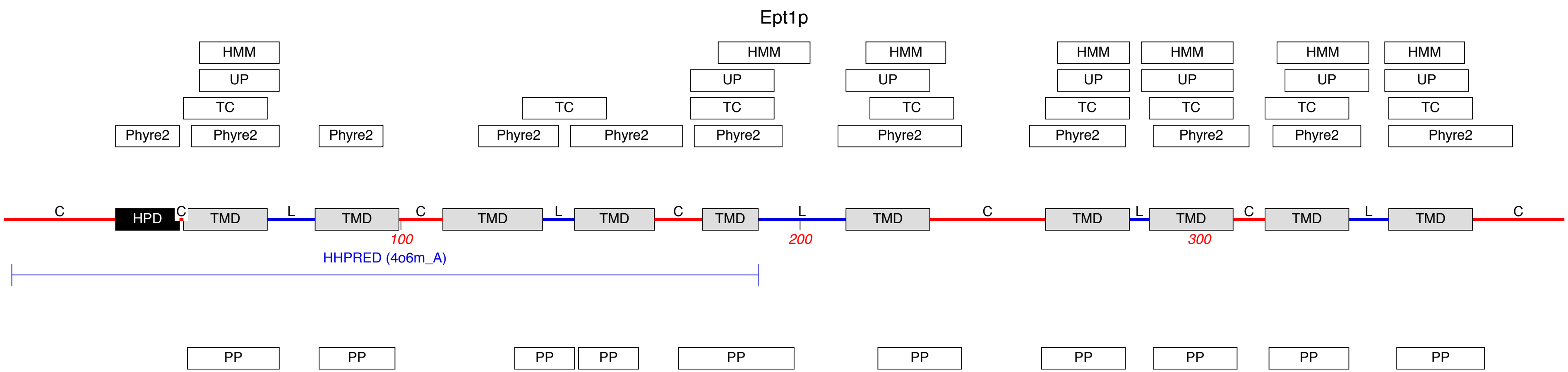
Ept1p
HMM
UP
TC
Phyre2
C
HPD
TMD
L
100
200
300
HHPRED (4o6m_A)
PP

Erf2p

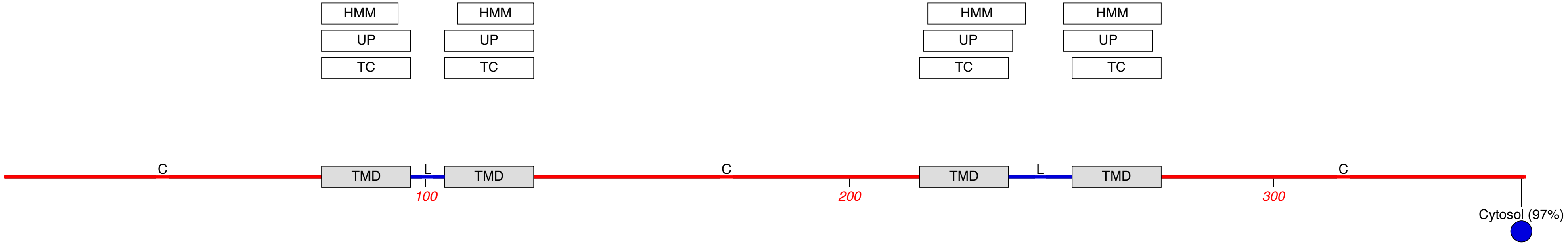
HMM
UP
TC
HMM
UP
TC
HMM
UP
TC
HMM
UP
TC
C
TMD
L
TMD
C
TMD
L
TMD
C
100
200
300
Cytosol (97%)

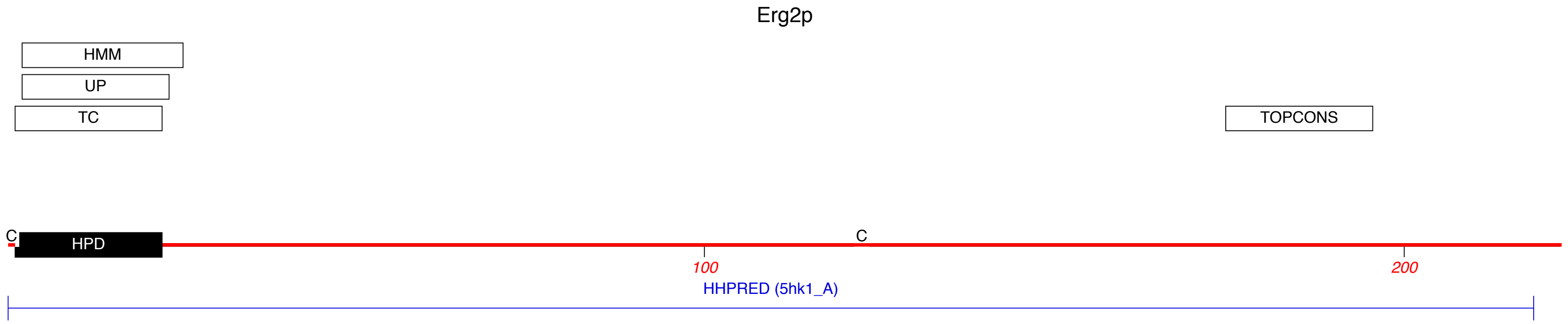

Erg2p
HMM
UP
TC
TOPCONS
C
HPD
C
100
200
HHPRED (5hk1_A)

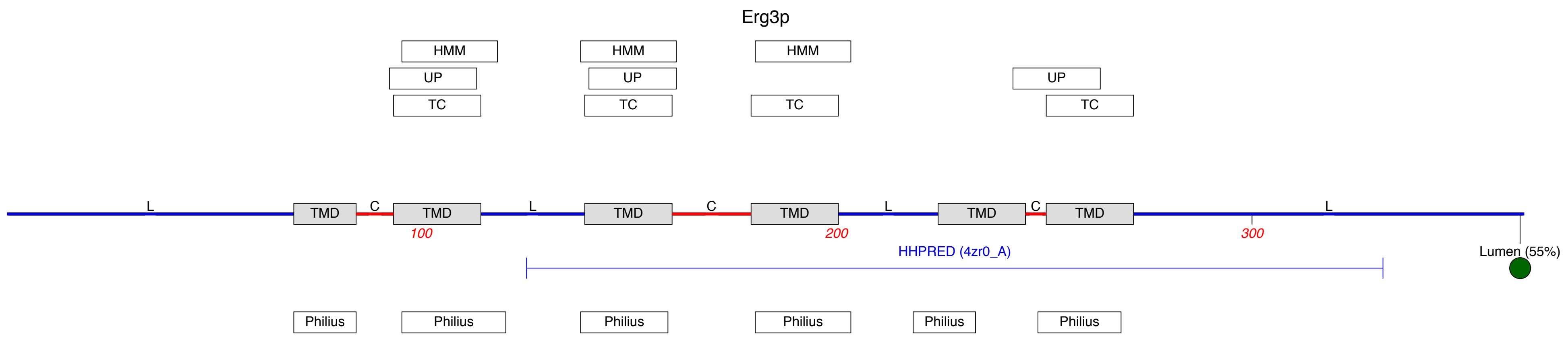

Erg3p
HMM
HMM
HMM
UP
UP
UP
TC
TC
TC
TC
L
TMD
C
TMD
L
TMD
C
TMD
L
TMD
C
TMD
L
100
200
300
HHPRED (4zr0_A)
Lumen (55%)
Philius
Philius
Philius
Philius
Philius
Philius

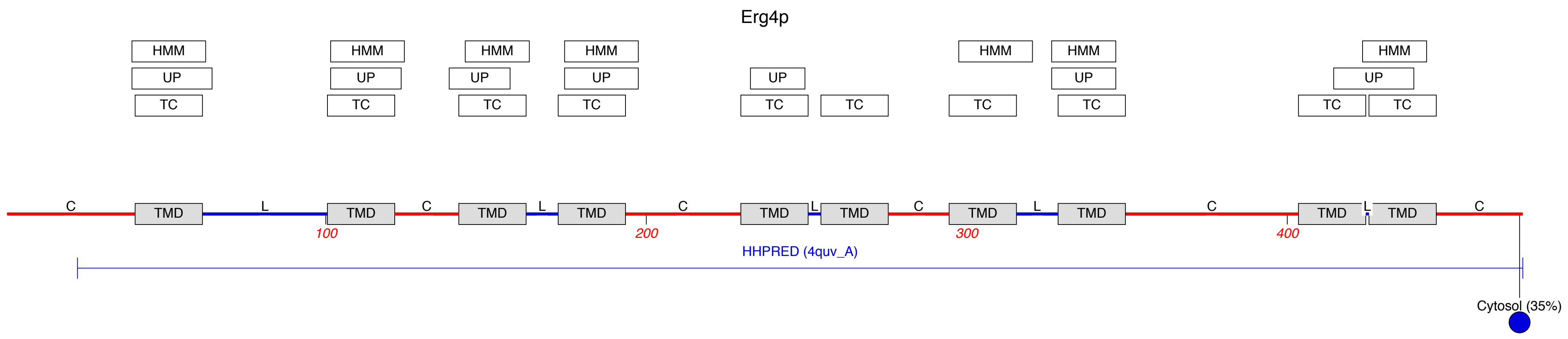
Erg4p
HMM
UP
TC
TMD
C
L
100
200
300
400
HHPRED (4quv_A)
Cytosol (35%)

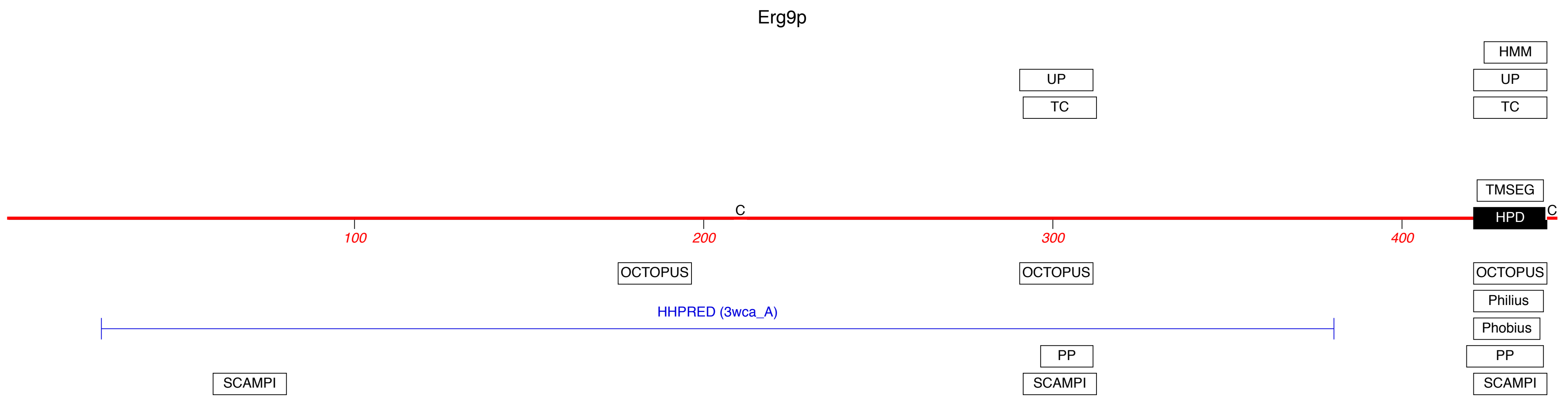
Erg9p
HMM
UP
TC
UP
TC
TMSEG
C
C
HPD
100
200
300
400
OCTOPUS
OCTOPUS
OCTOPUS
Philius
HHPRED (3wca_A)
Phobius
PP
PP
SCAMPI
SCAMPI
SCAMPI

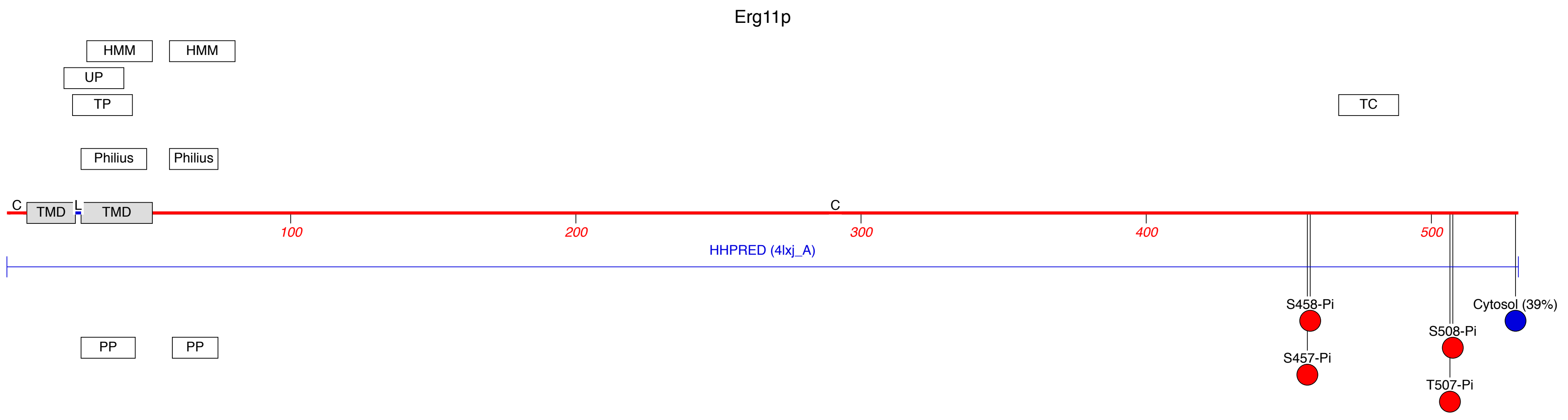
Erg11p
HMM
HMM
UP
TP
TC
Philius
Philius
C
TMD
L
TMD
C
100
200
300
400
500
HHPRED (4lxj_A)
S458-Pi
Cytosol (39%)
S508-Pi
PP
PP
S457-Pi
T507-Pi

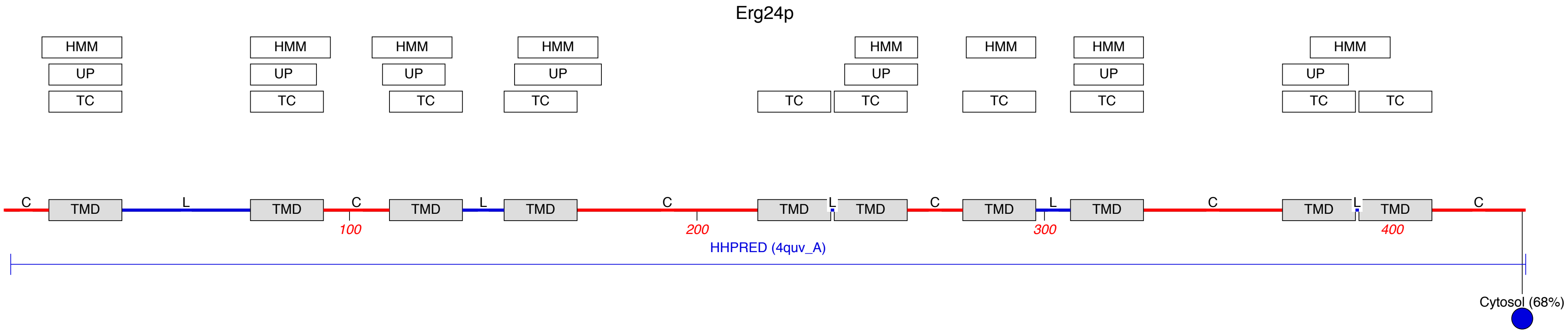
Erg24p
HMM
UP
TC
TMD
C
L
100
200
300
400
HHPRED (4quv_A)
Cytosol (68%)

Erg25p

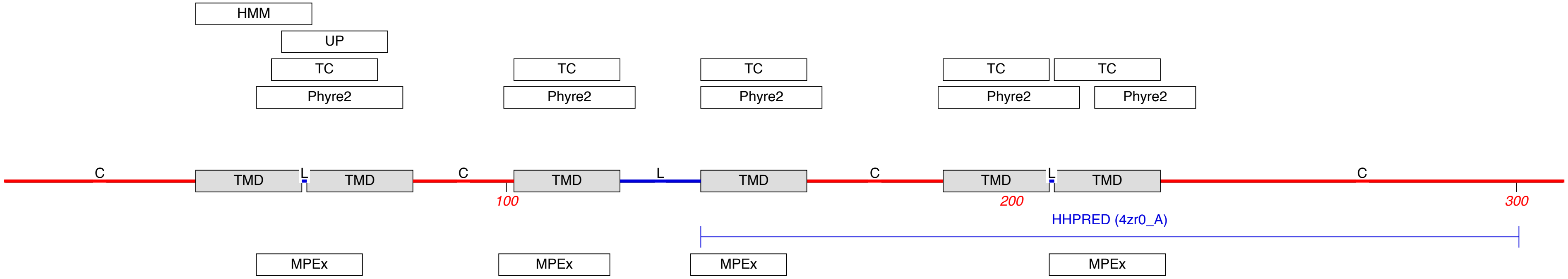

HMM
UP
TC
TC
TC
TC
TC
Phyre2
Phyre2
Phyre2
Phyre2
Phyre2
C
TMD
L
TMD
C
TMD
L
TMD
C
TMD
L
TMD
C
100
200
300
HHPRED (4zr0_A)
MPEx
MPEx
MPEx
MPEx

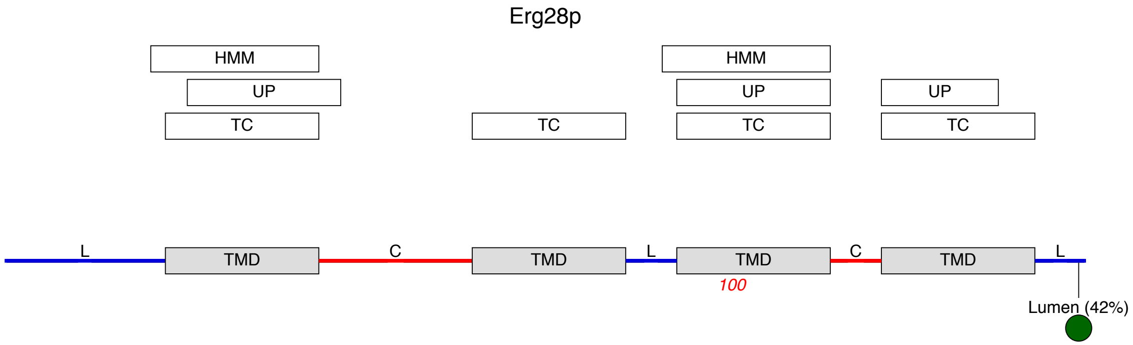
Erg28p
HMM
UP
TC
TC
HMM
UP
TC
UP
TC
L
TMD
C
TMD
L
TMD
100
C
TMD
L
Lumen (42%)

Fks1p

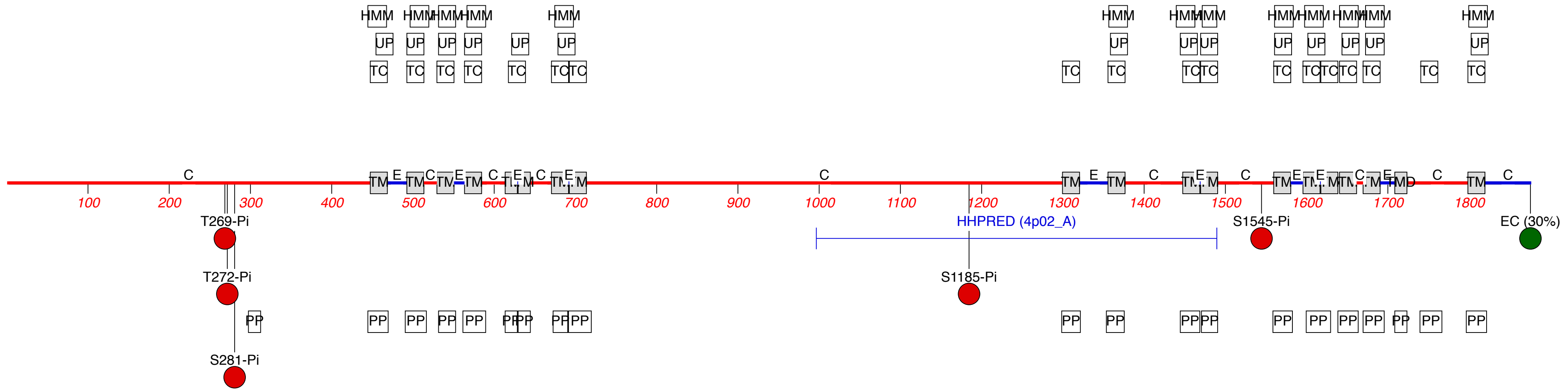

HMM
UP
TC
C
E
TM
TMD
100
200
300
400
500
600
700
800
900
1000
1100
1200
1300
1400
1500
1600
1700
1800
T269-Pi
T272-Pi
S281-Pi
HHPRED (4p02_A)
S1185-Pi
S1545-Pi
EC (30%)
PP

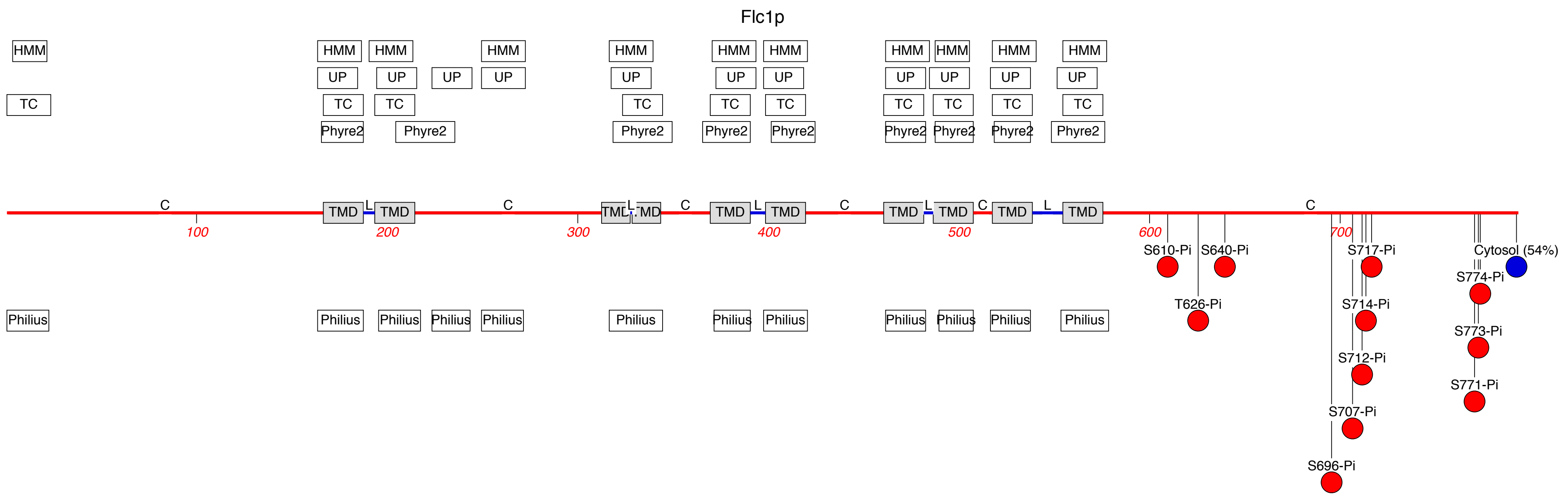

Flc1p
HMM
UP
TC
Phyre2
C
L
TMD
100
200
300
400
500
600
700
S610-Pi
S640-Pi
T626-Pi
S717-Pi
S714-Pi
S712-Pi
S707-Pi
S696-Pi
Cytosol (54%)
S774-Pi
S773-Pi
S771-Pi
Philius

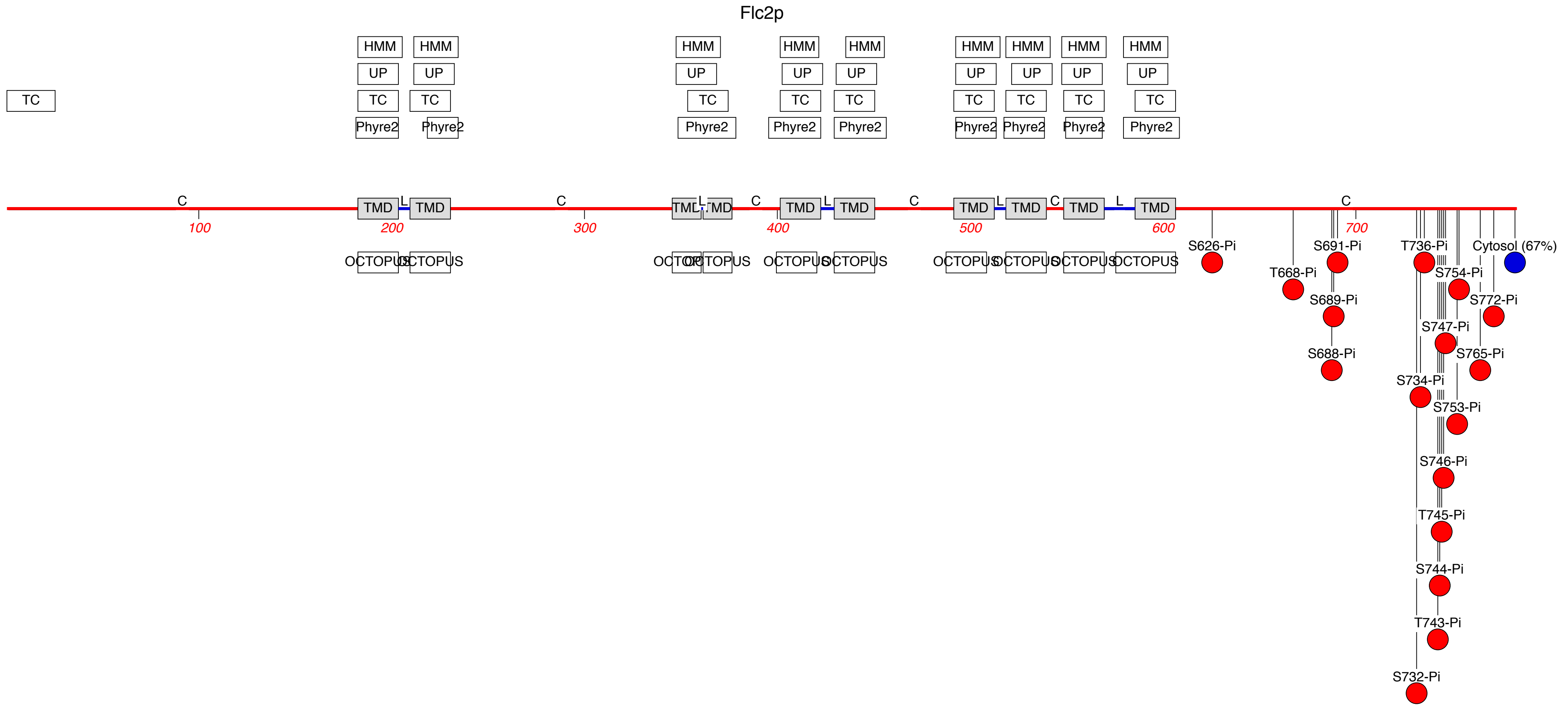

Flc2p
HMM
UP
TC
Phyre2
TMD
L
C
OCTOPUS
100
200
300
400
500
600
700
S626-Pi
T668-Pi
S691-Pi
S689-Pi
S688-Pi
T736-Pi
S754-Pi
S772-Pi
S747-Pi
S765-Pi
S734-Pi
S753-Pi
S746-Pi
T745-Pi
S744-Pi
T743-Pi
S732-Pi
Cytosol (67%)

Flc3p

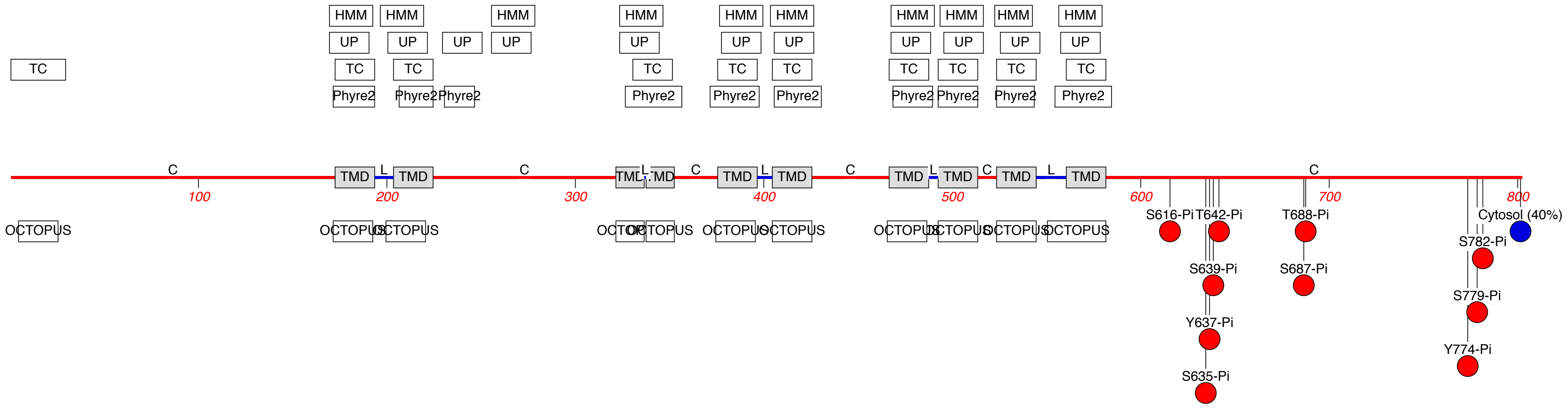

HMM
UP
TC
Phyre2
C
L
TMD
100
200
300
400
500
600
700
800
OCTOPUS
S616-Pi
T642-Pi
S639-Pi
Y637-Pi
S635-Pi
T688-Pi
S687-Pi
Cytosol (40%)
S782-Pi
S779-Pi
Y774-Pi

Gpi8p

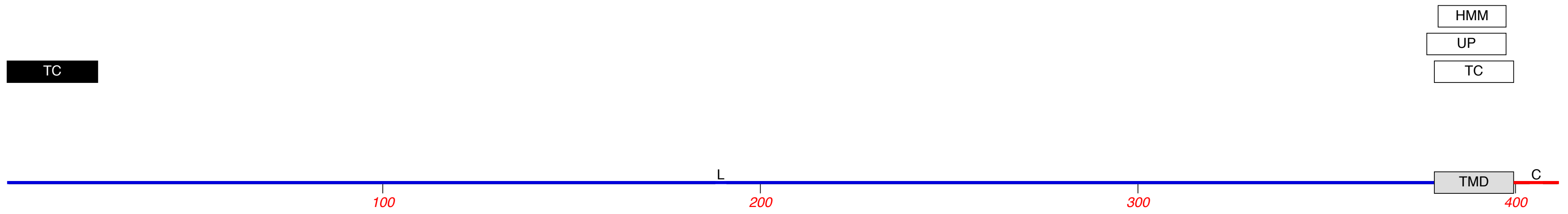

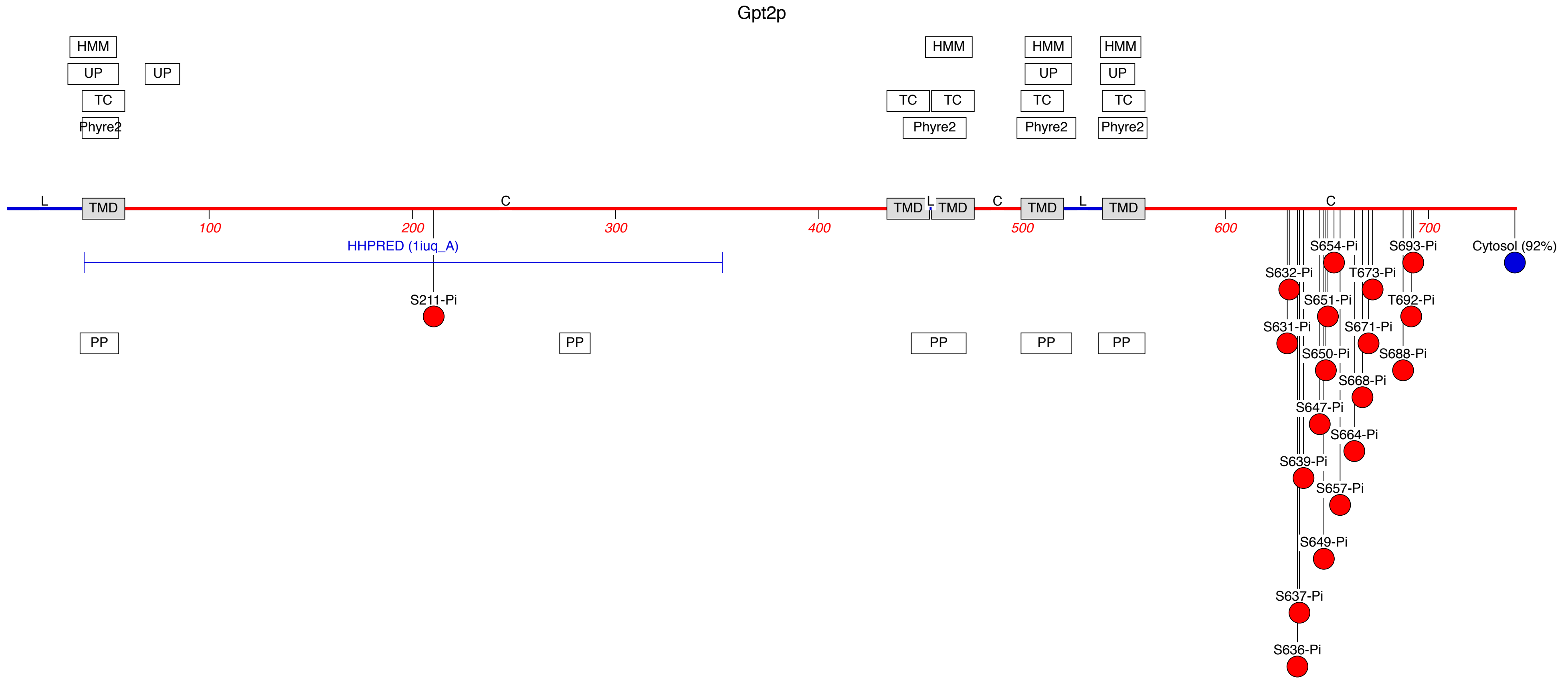
Gpt2p
HMM
UP
UP
TC
Phyre2
HMM
TC
TC
Phyre2
HMM
UP
TC
Phyre2
HMM
UP
TC
Phyre2
L
TMD
C
TMD
L
TMD
C
TMD
L
TMD
C
100
200
300
400
500
600
700
HHPRED (1iuq_A)
S211-Pi
PP
PP
PP
PP
PP
S654-Pi
S693-Pi
Cytosol (92%)
S632-Pi
T673-Pi
S651-Pi
T692-Pi
S631-Pi
S671-Pi
S650-Pi
S688-Pi
S668-Pi
S647-Pi
S664-Pi
S639-Pi
S657-Pi
S649-Pi
S637-Pi
S636-Pi

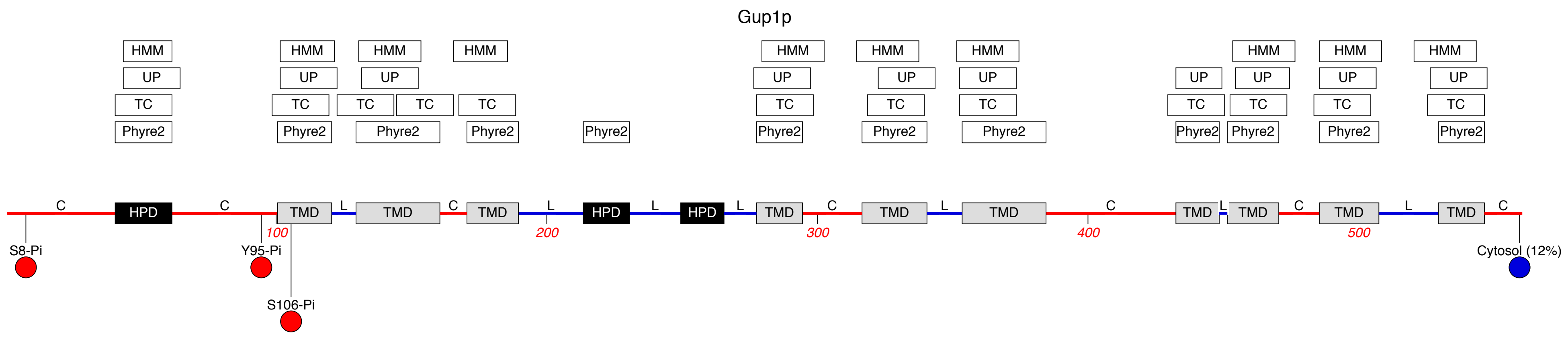
Gup1p
HMM
UP
TC
Phyre2
C
HPD
L
TMD
100
200
300
400
500
S8-Pi
Y95-Pi
S106-Pi
Cytosol (12%)

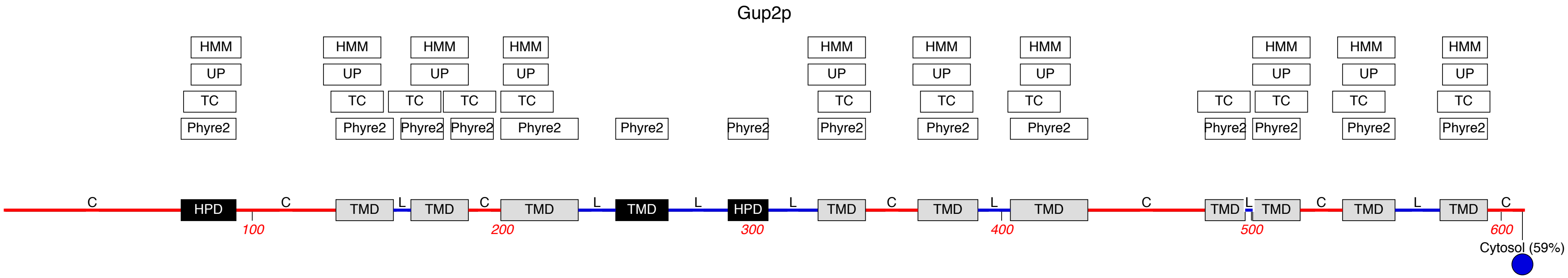
Gup2p
HMM
UP
TC
Phyre2
HPD
TMD
C
L
100
200
300
400
500
600
Cytosol (59%)

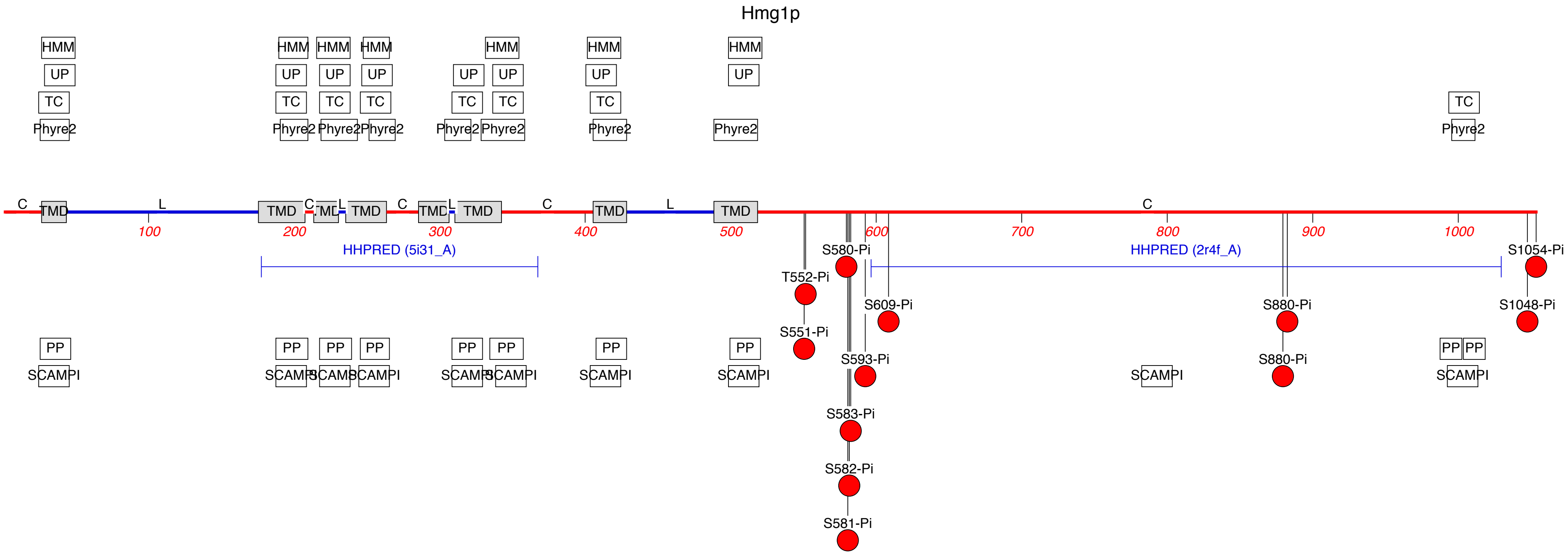
Hmg1p
HMM
UP
TC
Phyre2
TMD
PP
SCAMPI
C
L
100
200
300
400
500
600
700
800
900
1000
HHPRED (5i31_A)
HHPRED (2r4f_A)
T552-Pi
S551-Pi
S580-Pi
S593-Pi
S583-Pi
S582-Pi
S581-Pi
S609-Pi
S880-Pi
S880-Pi
S1054-Pi
S1048-Pi

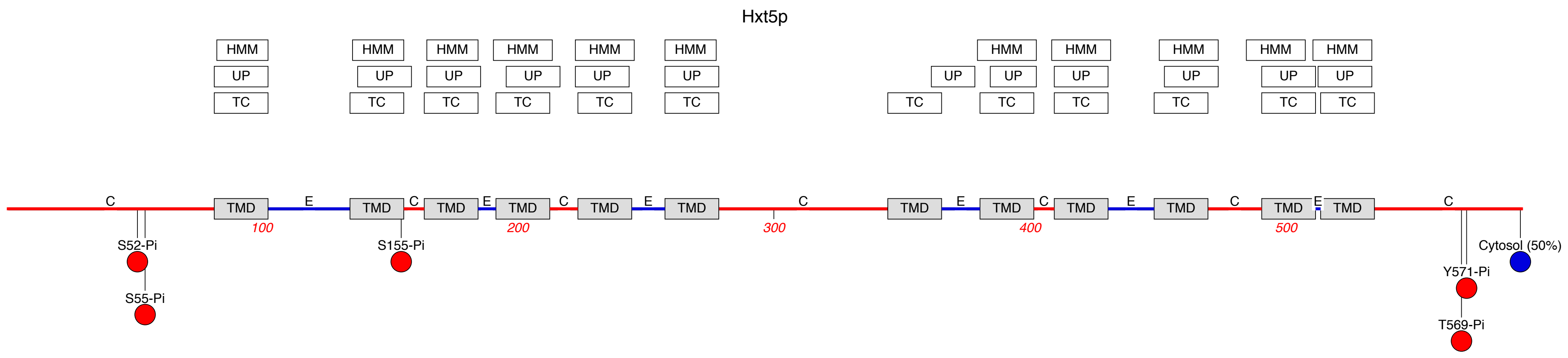

Hxt5p
HMM
UP
TC
TMD
C
E
100
200
300
400
500
S52-Pi
S55-Pi
S155-Pi
Cytosol (50%)
Y571-Pi
T569-Pi

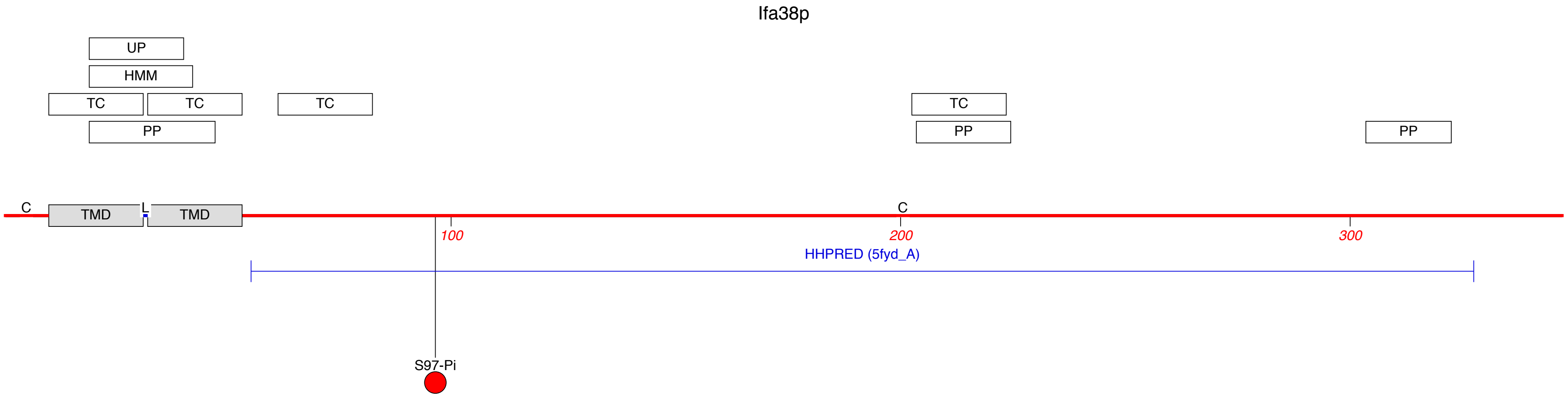
Ifa38p
UP
HMM
TC
TC
TC
TC
PP
PP
PP
C
TMD
L
TMD
C
100
200
300
HHPRED (5fyd_A)
S97-Pi

Ipt1p

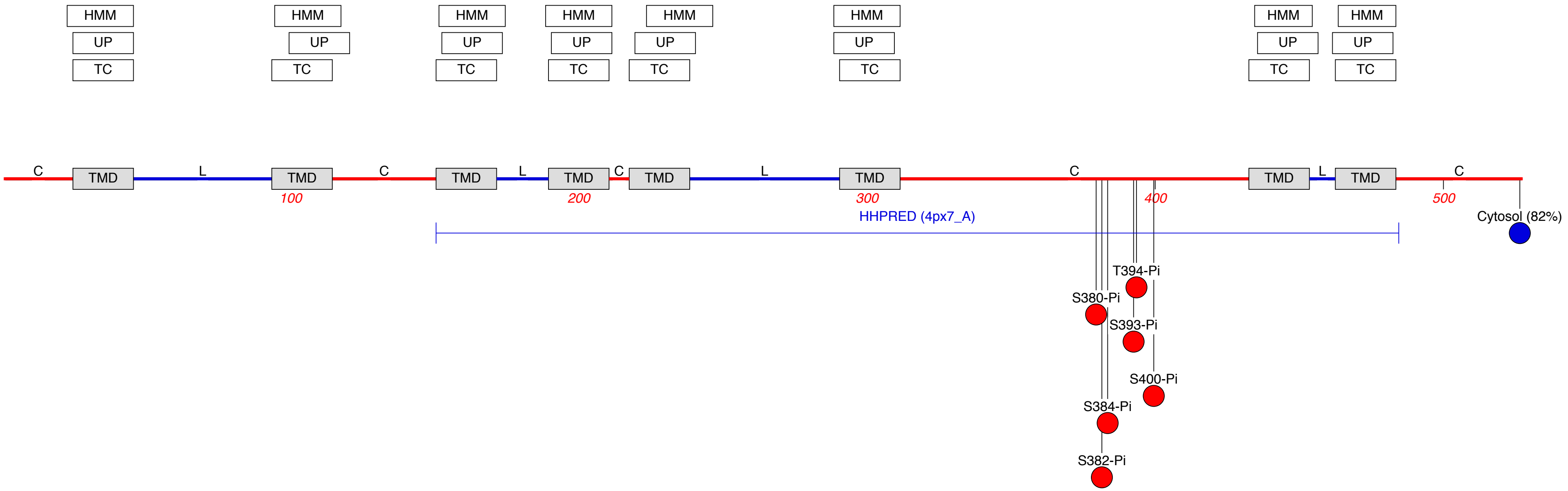

HMM
UP
TC
TMD
C
L
100
200
300
400
500
HHPRED (4px7_A)
Cytosol (82%)
T394-Pi
S380-Pi
S393-Pi
S400-Pi
S384-Pi
S382-Pi

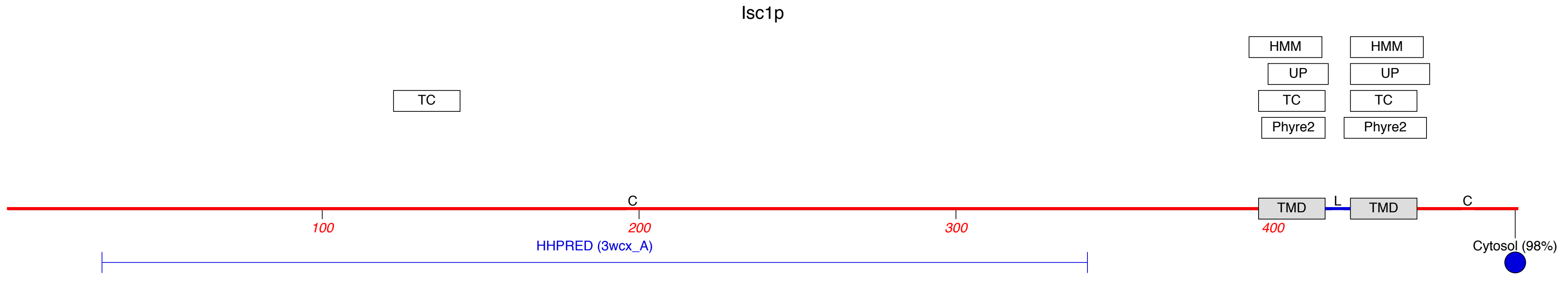

Isc1p
HMM
HMM
UP
UP
TC
TC
TC
Phyre2
Phyre2
C
TMD
L
TMD
C
100
200
300
400
HHPRED (3wcx_A)
Cytosol (98%)

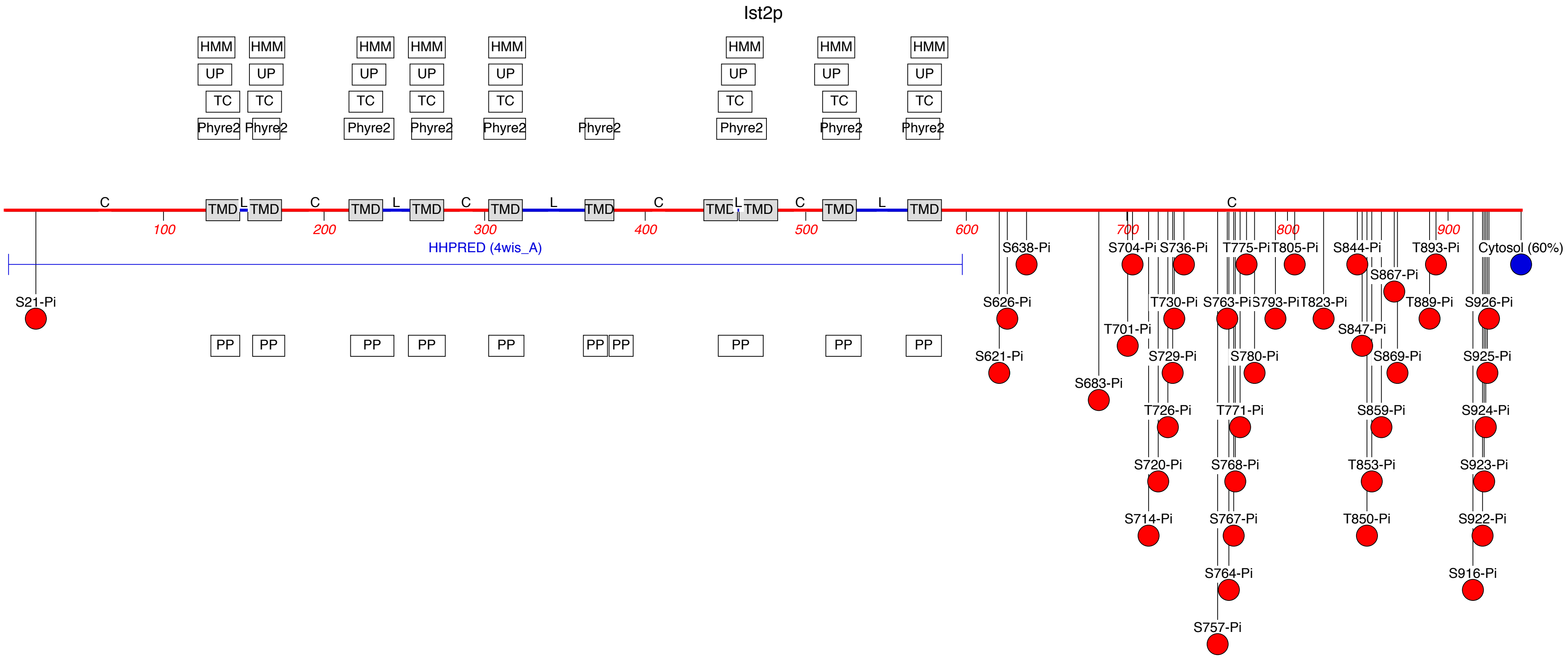

Ist2p
HMM
UP
TC
Phyre2
TMD
L
C
100
200
300
400
500
600
700
800
900
HHPRED (4wis_A)
PP
S21-Pi
S621-Pi
S626-Pi
S638-Pi
S683-Pi
T701-Pi
S704-Pi
S714-Pi
S720-Pi
T726-Pi
S729-Pi
T730-Pi
S736-Pi
S757-Pi
S763-Pi
S764-Pi
S767-Pi
S768-Pi
T771-Pi
T775-Pi
S780-Pi
S793-Pi
T805-Pi
T823-Pi
S844-Pi
S847-Pi
T850-Pi
T853-Pi
S859-Pi
S867-Pi
S869-Pi
T889-Pi
T893-Pi
S916-Pi
S922-Pi
S923-Pi
S924-Pi
S925-Pi
S926-Pi
Cytosol (60%)

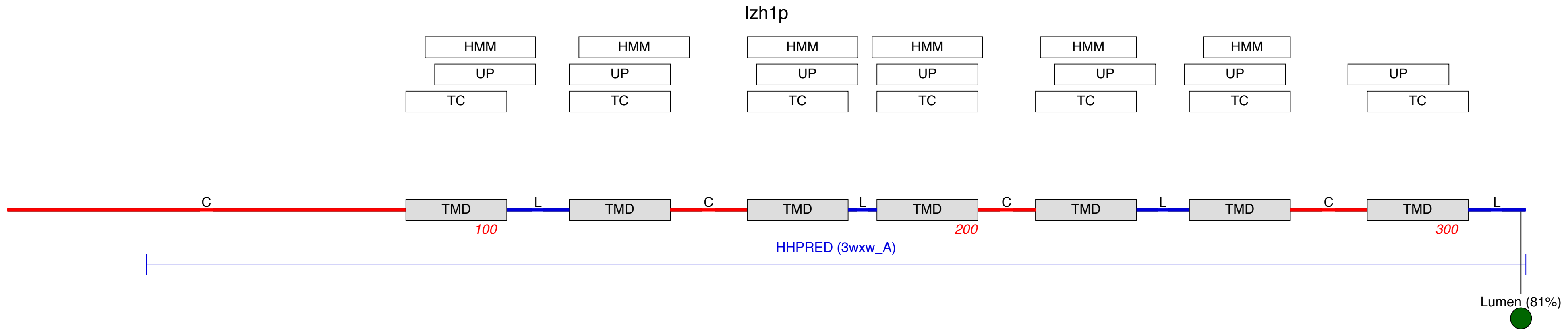
Izh1p
HMM
HMM
HMM
HMM
HMM
HMM
UP
UP
UP
UP
UP
UP
UP
TC
TC
TC
TC
TC
TC
TC
C
TMD
L
TMD
C
TMD
L
TMD
C
TMD
L
TMD
C
TMD
L
100
200
300
HHPRED (3wxw_A)
Lumen (81%)

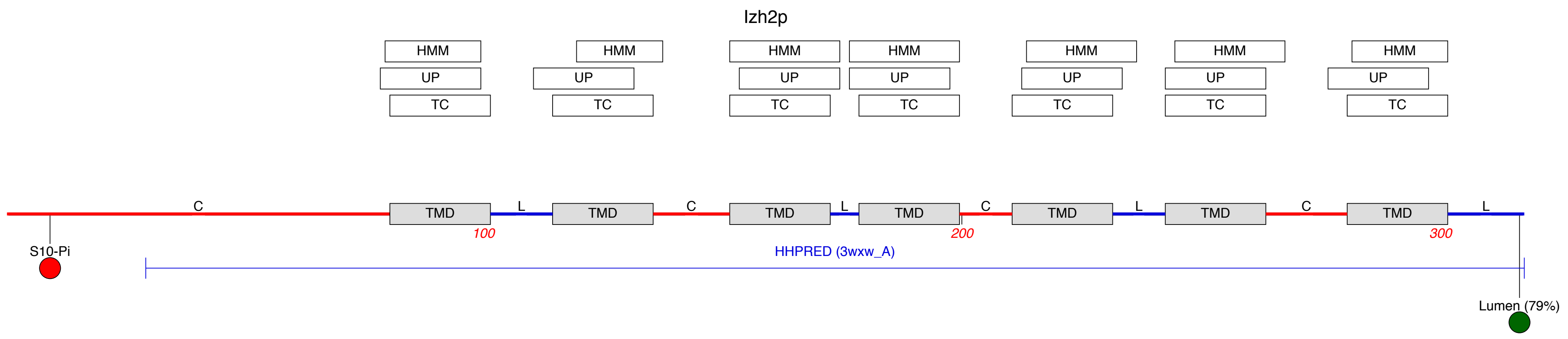
Izh2p
HMM
UP
TC
TMD
C
L
100
200
300
S10-Pi
HHPRED (3wxw_A)
Lumen (79%)

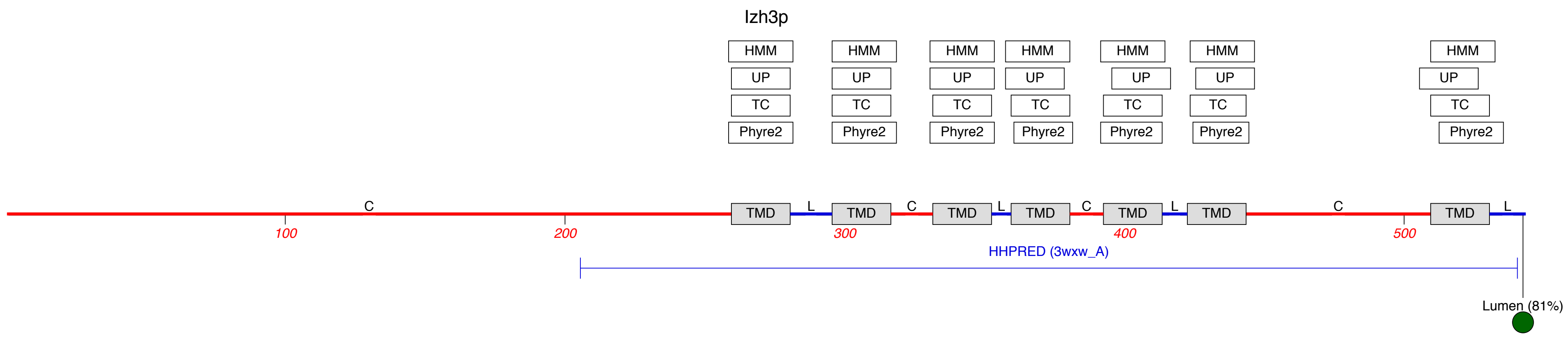
Izh3p
HMM
UP
TC
Phyre2
TMD
C
L
100
200
300
400
500
HHPRED (3wxw_A)
Lumen (81%)

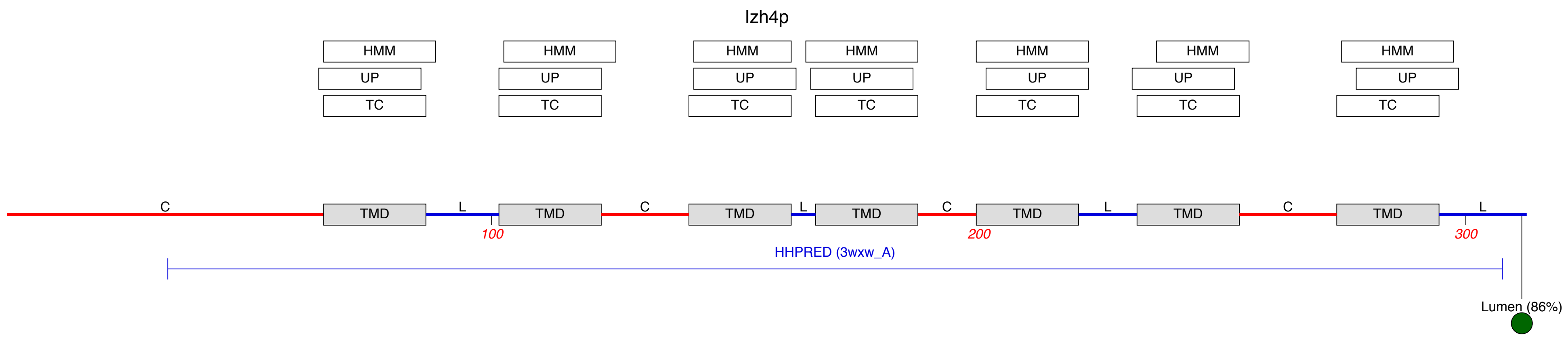
Izh4p
HMM
UP
TC
TMD
C
L
100
200
300
HHPRED (3wxw_A)
Lumen (86%)

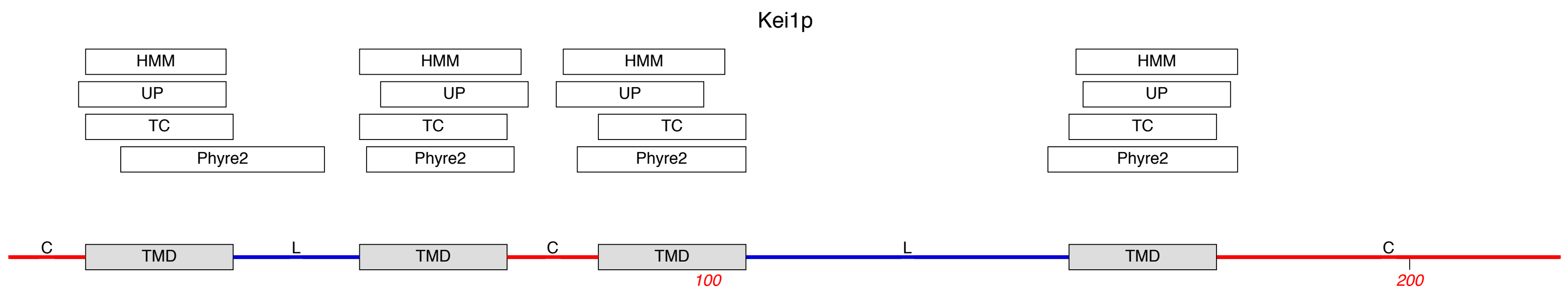

Kei1p
HMM
UP
TC
Phyre2
HMM
UP
TC
Phyre2
HMM
UP
TC
Phyre2
HMM
UP
TC
Phyre2
C
TMD
L
TMD
C
TMD
100
L
TMD
C
200

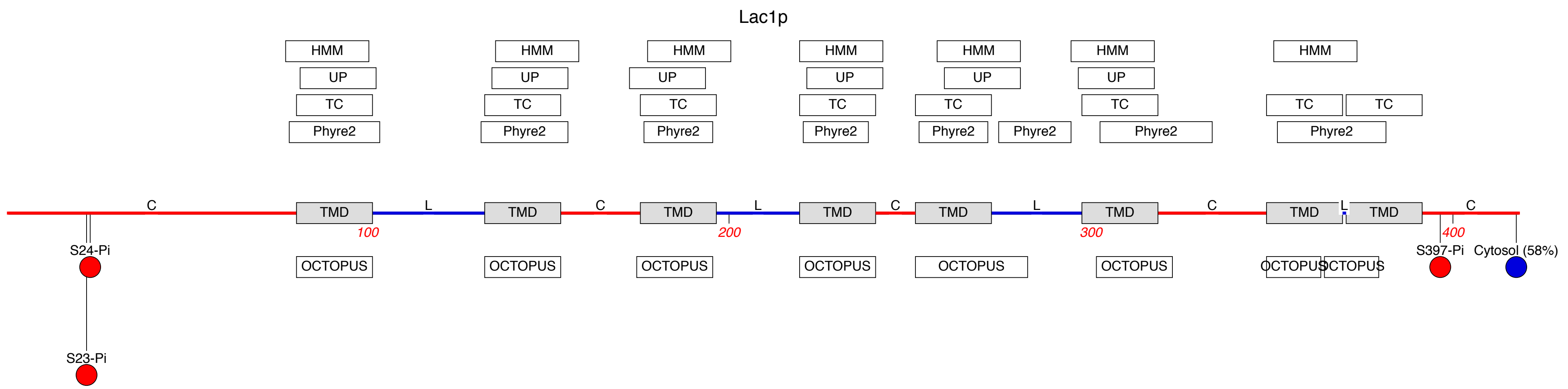
Lac1p
HMM
UP
TC
Phyre2
TMD
C
L
100
200
300
400
OCTOPUS
S24-Pi
S23-Pi
S397-Pi
Cytosol (58%)

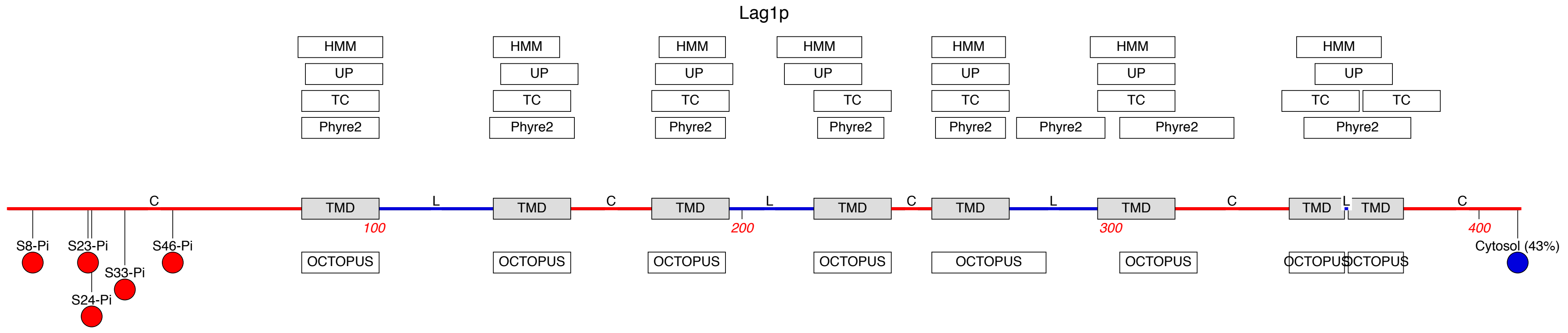
Lag1p
HMM
UP
TC
Phyre2
TMD
OCTOPUS
L
C
100
200
300
400
S8-Pi
S23-Pi
S24-Pi
S33-Pi
S46-Pi
Cytosol (43%)

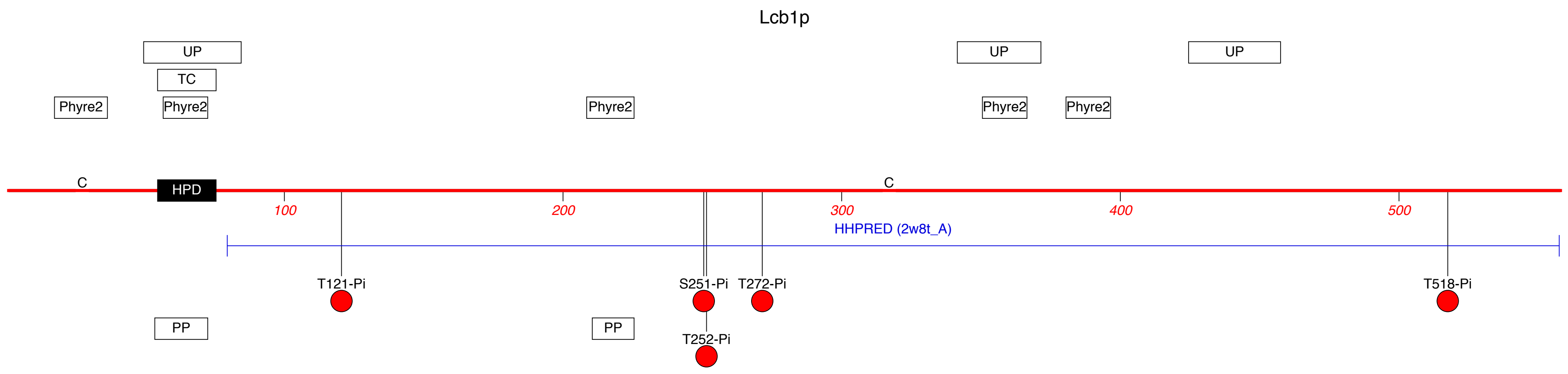
Lcb1p
UP
UP
UP
TC
Phyre2
Phyre2
Phyre2
Phyre2
Phyre2
C
HPD
C
100
200
300
400
500
HHPRED (2w8t_A)
T121-Pi
S251-Pi
T272-Pi
T518-Pi
T252-Pi
PP
PP

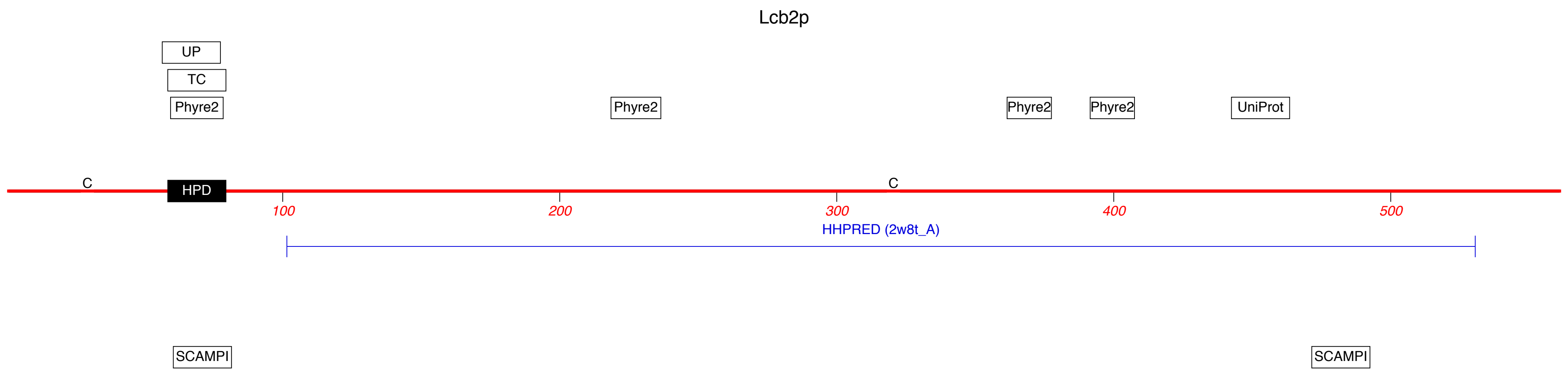
Lcb2p
UP
TC
Phyre2
Phyre2
Phyre2
Phyre2
UniProt
C
HPD
C
100
200
300
400
500
HHPRED (2w8t_A)
SCAMPI
SCAMPI

Lcb3p

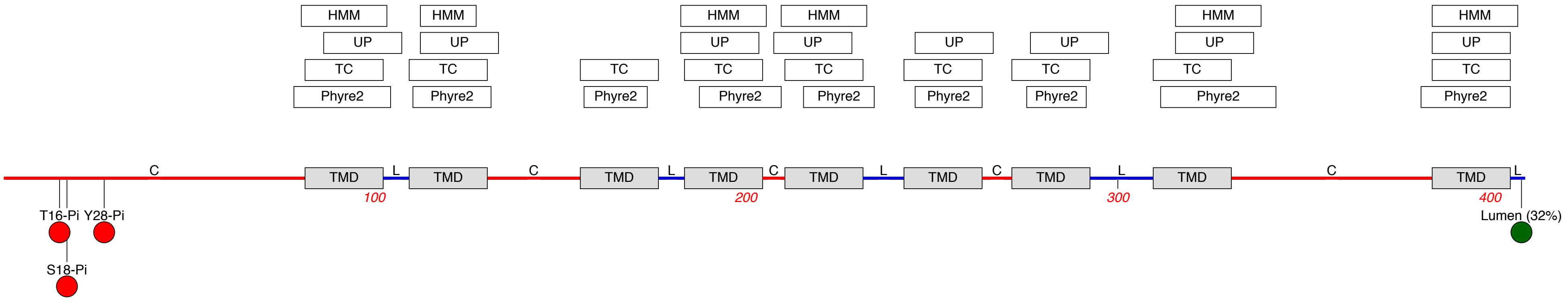

HMM
UP
TC
Phyre2
TMD
C
L
100
200
300
400
T16-Pi
Y28-Pi
S18-Pi
Lumen (32%)

Lpp1p

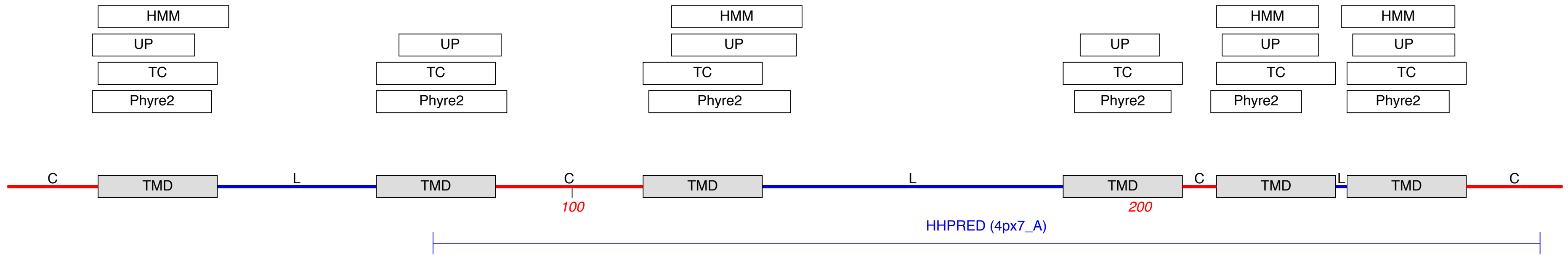

Mir1p

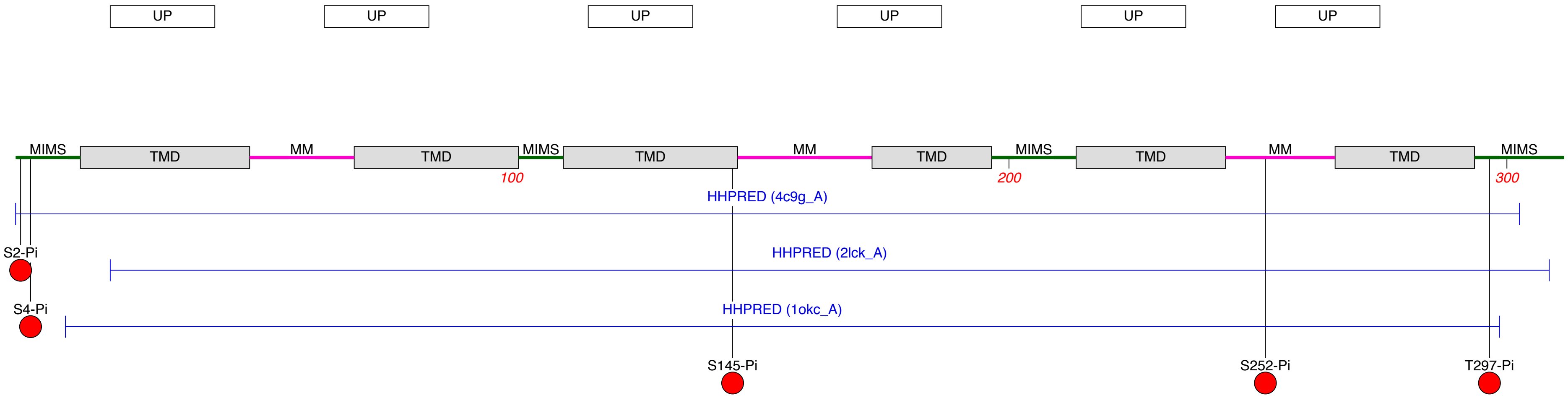
UP
UP
UP
UP
UP
UP
MIMS
TMD
MM
TMD
MIMS
TMD
MM
TMD
MIMS
TMD
MM
TMD
MIMS
100
200
300
HHPRED (4c9g_A)
S2-Pi
HHPRED (2lck_A)
S4-Pi
HHPRED (1okc_A)
S145-Pi
S252-Pi
T297-Pi

Neo1p

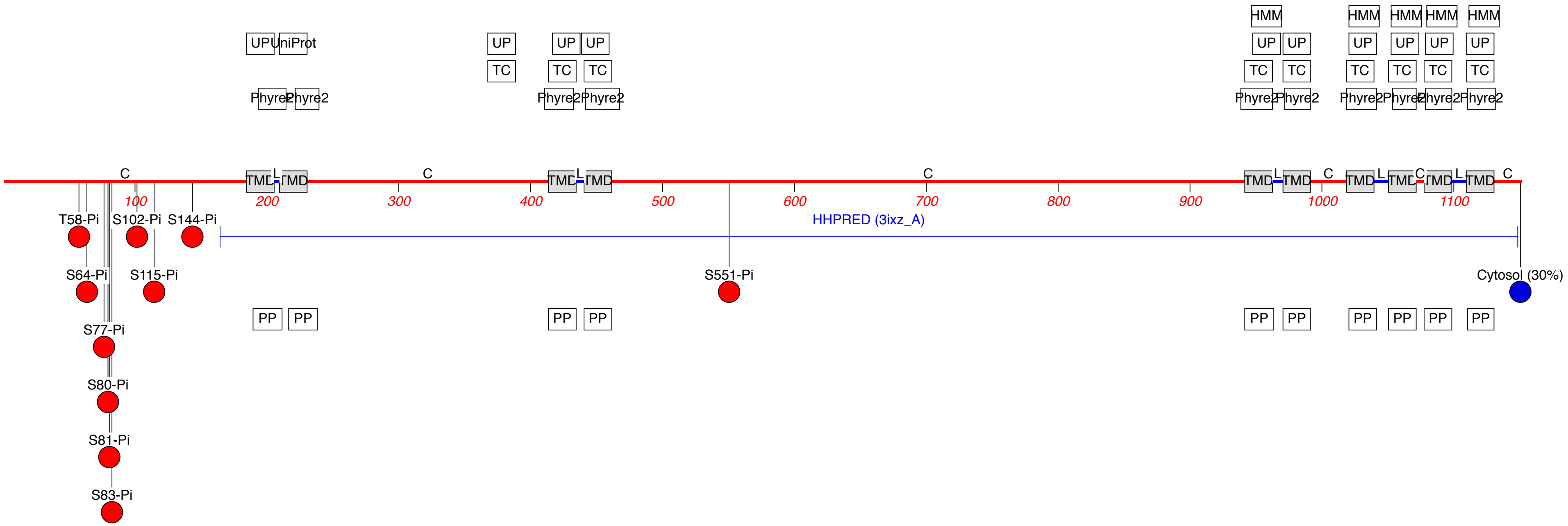
HMM
UP
UniProt
TC
Phyre2
C
L
TMD
100
200
300
400
500
600
700
800
900
1000
1100
HHPRED (3ixz_A)
T58-Pi
S64-Pi
S77-Pi
S80-Pi
S81-Pi
S83-Pi
S102-Pi
S115-Pi
S144-Pi
S551-Pi
Cytosol (30%)
PP

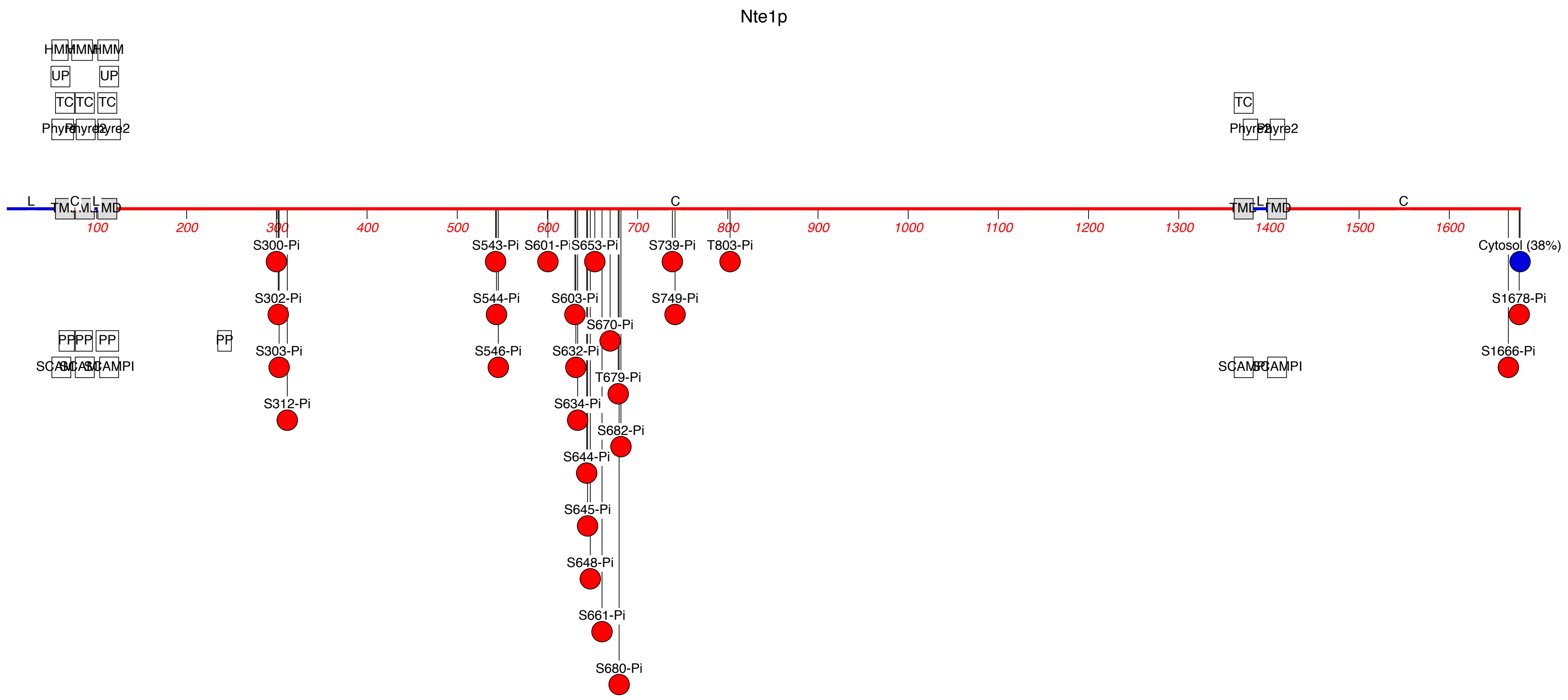
Nte1p
HMM
UP
TC
Phyre2
SCAMPI
PP
L
C
TMD
100
200
300
400
500
600
700
800
900
1000
1100
1200
1300
1400
1500
1600
S300-Pi
S302-Pi
S303-Pi
S312-Pi
S543-Pi
S544-Pi
S546-Pi
S601-Pi
S603-Pi
S632-Pi
S634-Pi
S644-Pi
S645-Pi
S648-Pi
S653-Pi
S661-Pi
S670-Pi
T679-Pi
S680-Pi
S682-Pi
S739-Pi
S749-Pi
T803-Pi
Cytosol (38%)
S1678-Pi
S1666-Pi

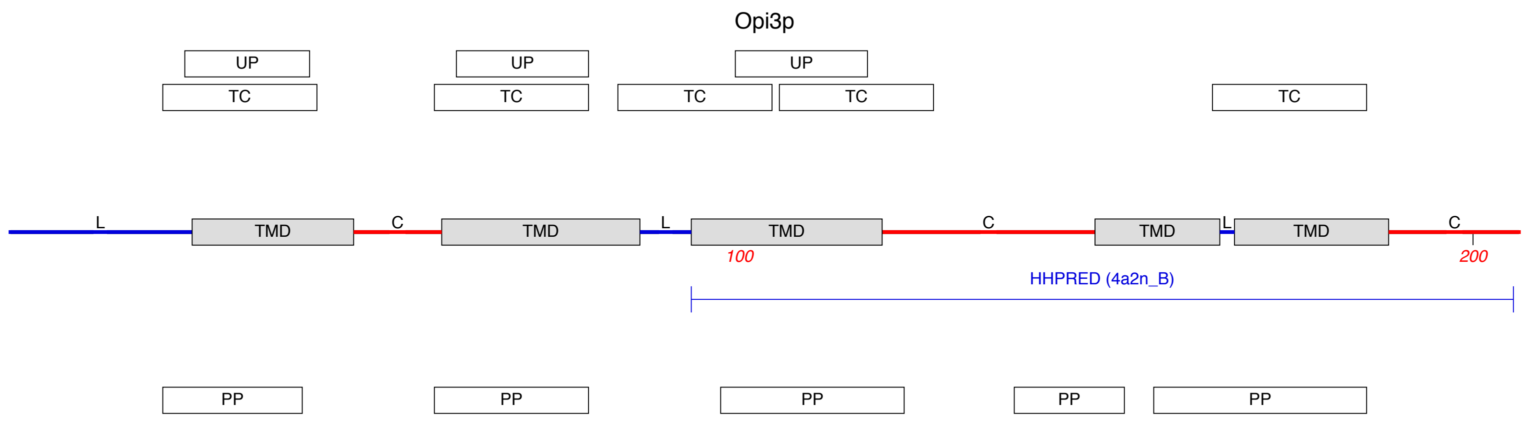
Opi3p
UP
UP
UP
TC
TC
TC
TC
TC
L
TMD
C
TMD
L
TMD
C
TMD
L
TMD
C
100
200
HHPRED (4a2n_B)
PP
PP
PP
PP
PP

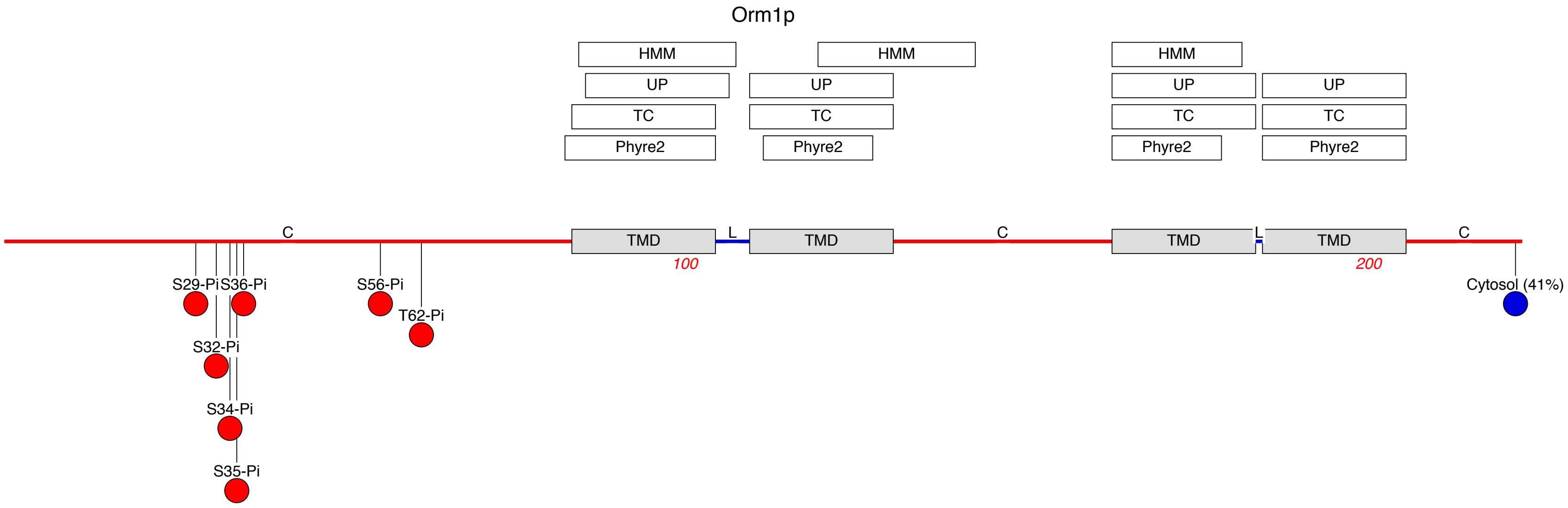
Orm1p
HMM
HMM
HMM
UP
UP
UP
UP
TC
TC
TC
TC
Phyre2
Phyre2
Phyre2
Phyre2
C
TMD
L
TMD
C
TMD
L
TMD
C
100
200
S29-Pi
S36-Pi
S32-Pi
S34-Pi
S35-Pi
S56-Pi
T62-Pi
Cytosol (41%)

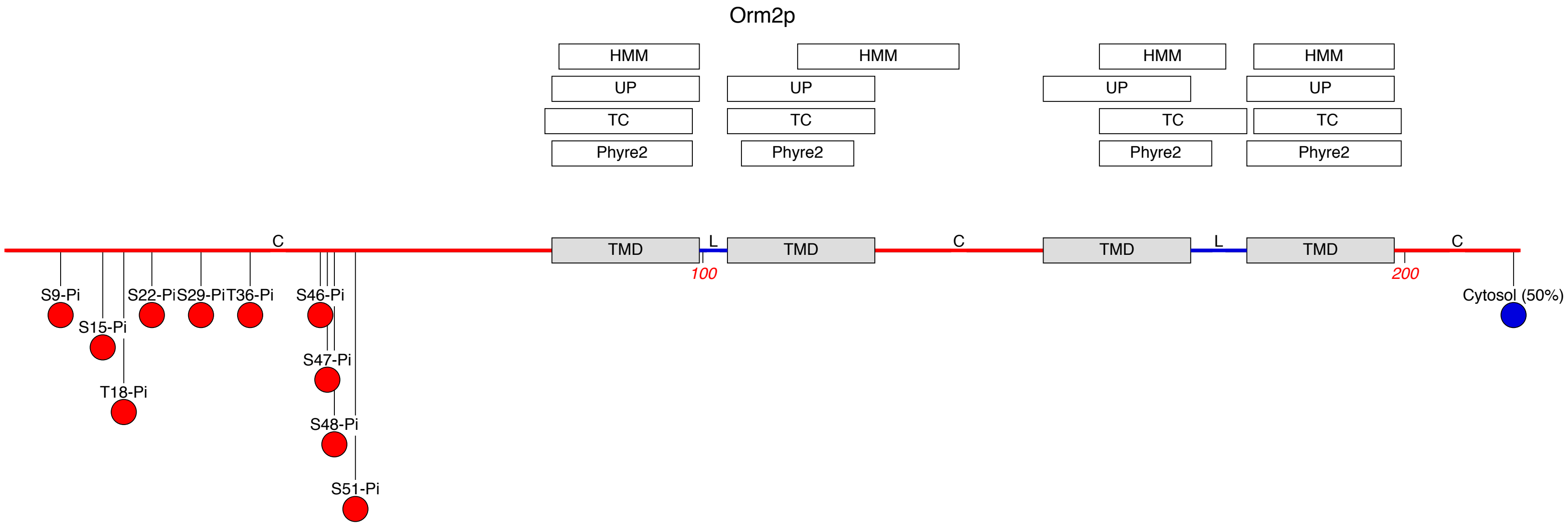
Orm2p
HMM
HMM
HMM
HMM
UP
UP
UP
UP
TC
TC
TC
TC
Phyre2
Phyre2
Phyre2
Phyre2
C
L
C
L
C
TMD
TMD
TMD
TMD
100
200
S9-Pi
S15-Pi
T18-Pi
S22-Pi
S29-Pi
T36-Pi
S46-Pi
S47-Pi
S48-Pi
S51-Pi
Cytosol (50%)

Ost1p

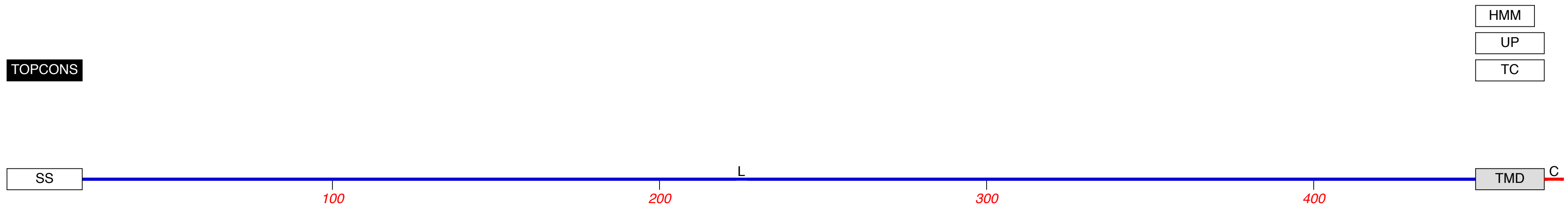

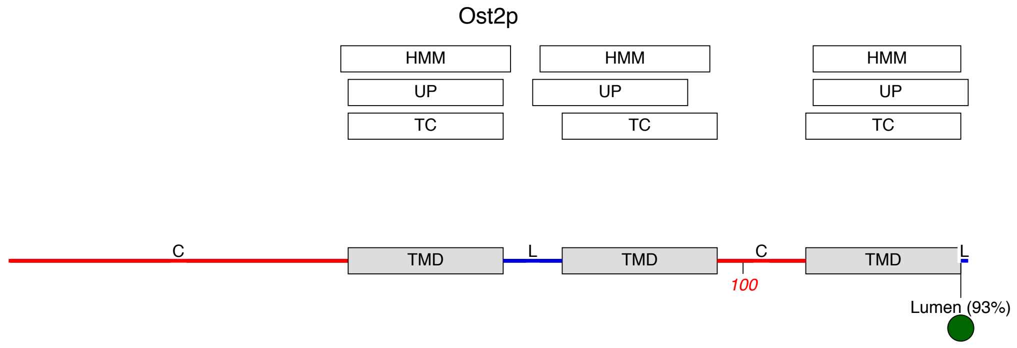
Ost2p
HMM
HMM
HMM
UP
UP
UP
TC
TC
TC
C
TMD
L
TMD
C
TMD
L
100
Lumen (93%)

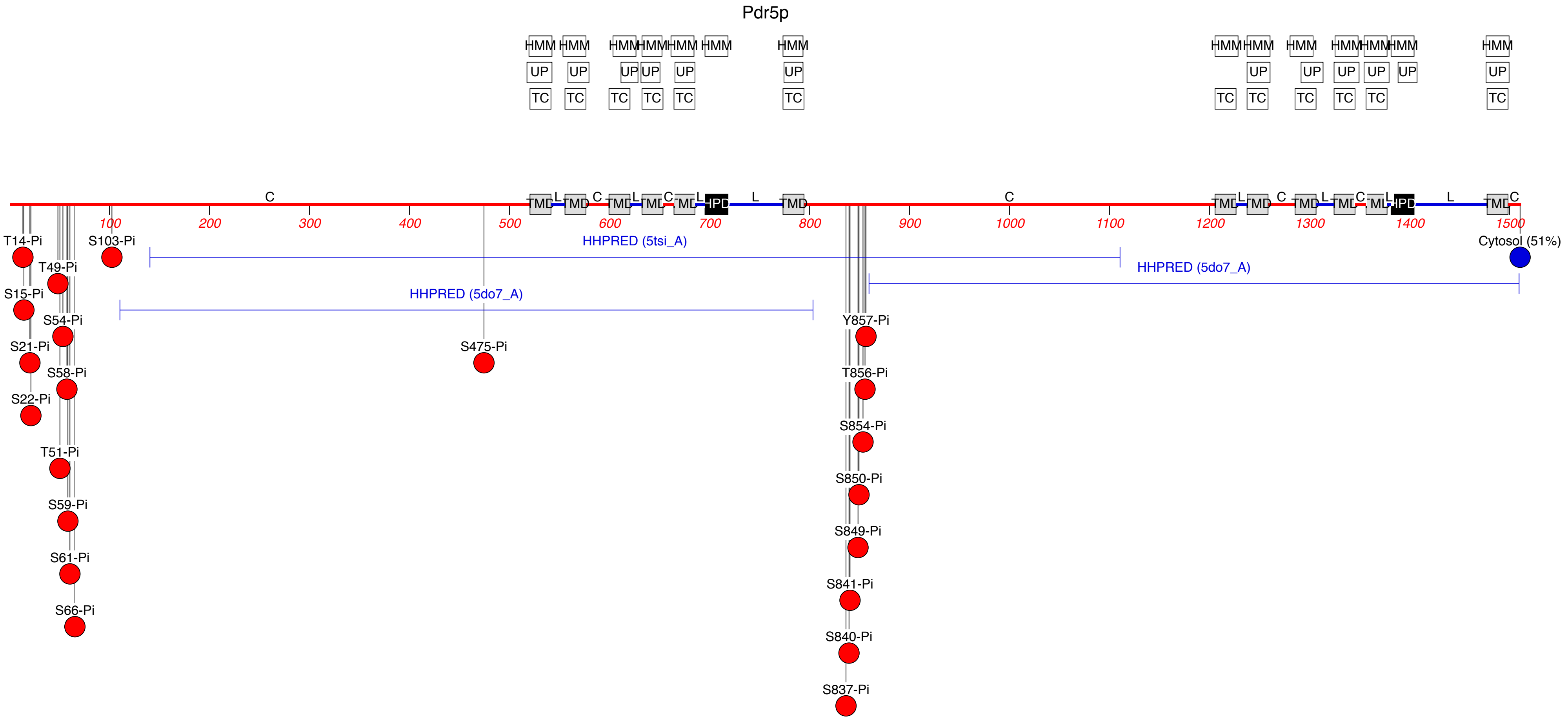
Pdr5p
HMM
UP
TC
TMD
HPD
C
L
100
200
300
400
500
600
700
800
900
1000
1100
1200
1300
1400
1500
HHPRED (5tsi_A)
HHPRED (5do7_A)
HHPRED (5do7_A)
Cytosol (51%)
T14-Pi
S15-Pi
S21-Pi
S22-Pi
T49-Pi
S54-Pi
S58-Pi
T51-Pi
S59-Pi
S61-Pi
S66-Pi
S103-Pi
S475-Pi
Y857-Pi
T856-Pi
S854-Pi
S850-Pi
S849-Pi
S841-Pi
S840-Pi
S837-Pi

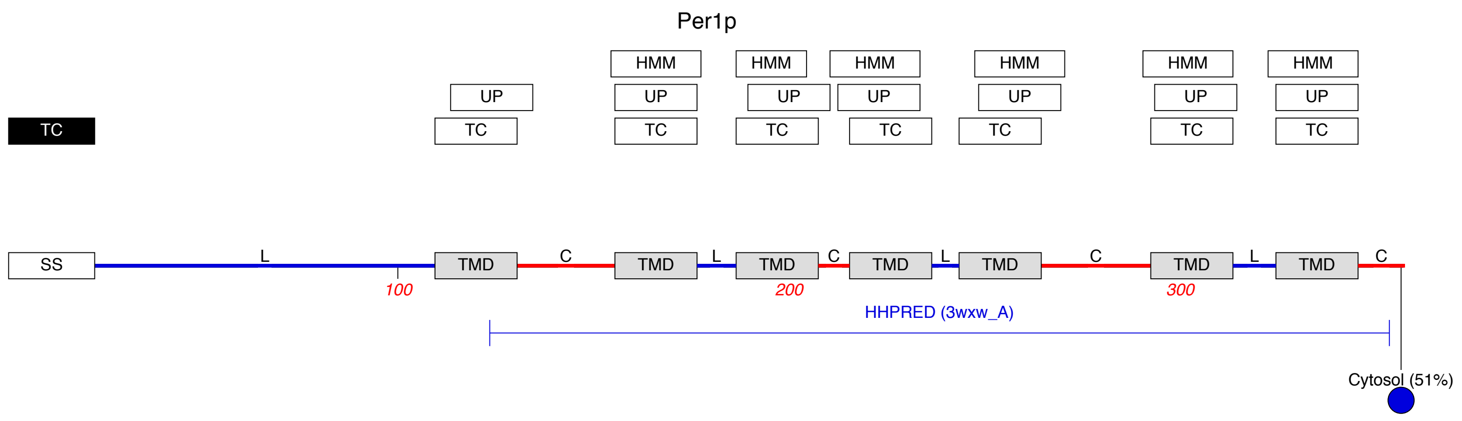

Per1p
HMM
UP
TC
SS
L
TMD
C
100
200
300
HHPRED (3wxw_A)
Cytosol (51%)

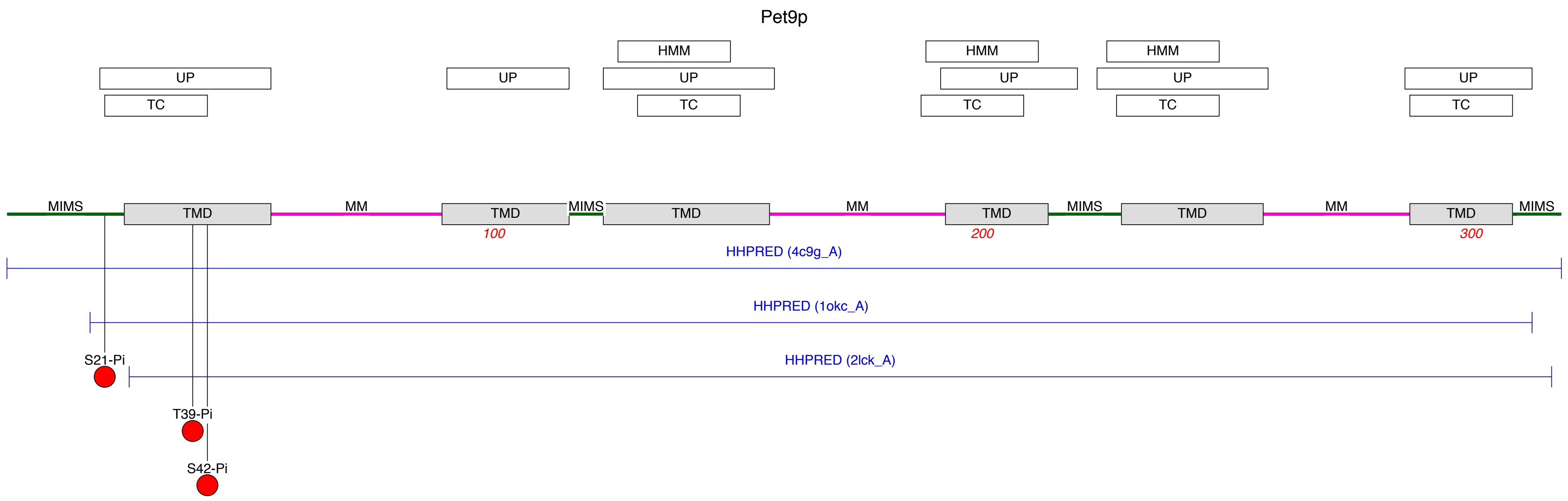

Pet9p
HMM
HMM
HMM
UP
UP
UP
UP
UP
UP
TC
TC
TC
TC
TC
MIMS
TMD
MM
TMD
MIMS
TMD
MM
TMD
MIMS
TMD
MM
TMD
MIMS
100
200
300
HHPRED (4c9g_A)
HHPRED (1okc_A)
HHPRED (2lck_A)
S21-Pi
T39-Pi
S42-Pi

Pfa3p

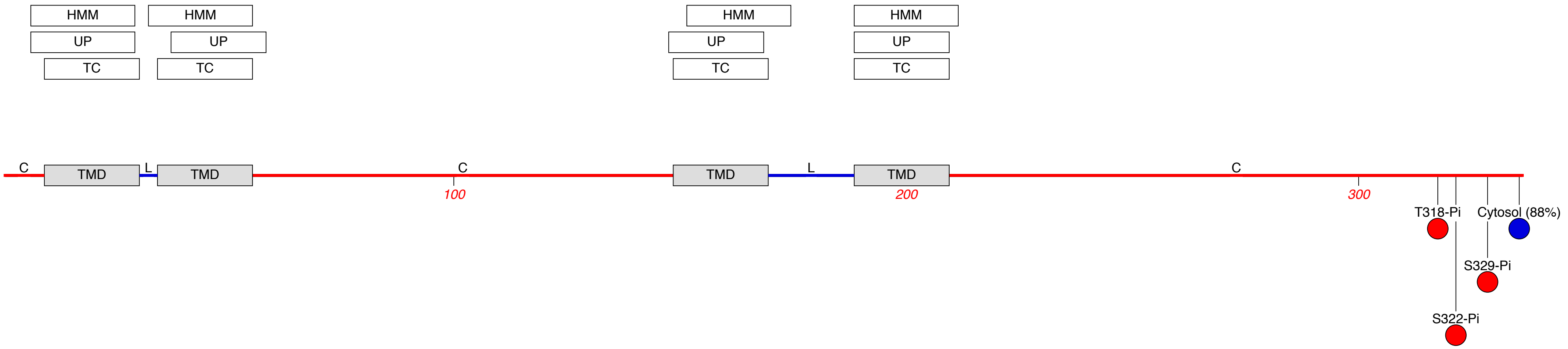
HMM
HMM
HMM
HMM
UP
UP
UP
UP
TC
TC
TC
TC
C
TMD
L
TMD
C
TMD
L
TMD
C
100
200
300
T318-Pi
Cytosol (88%)
S329-Pi
S322-Pi

Pfa4p

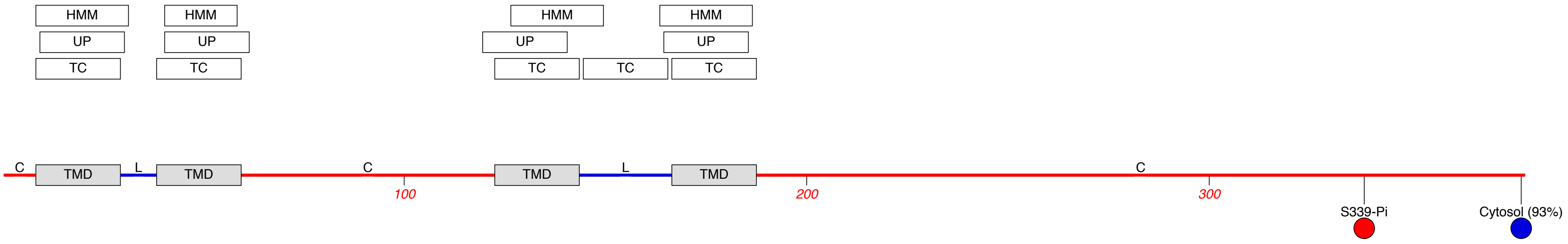
HMM
HMM
HMM
HMM
UP
UP
UP
UP
TC
TC
TC
TC
TC
C
TMD
L
TMD
C
TMD
L
TMD
C
100
200
300
S339-Pi
Cytosol (93%)

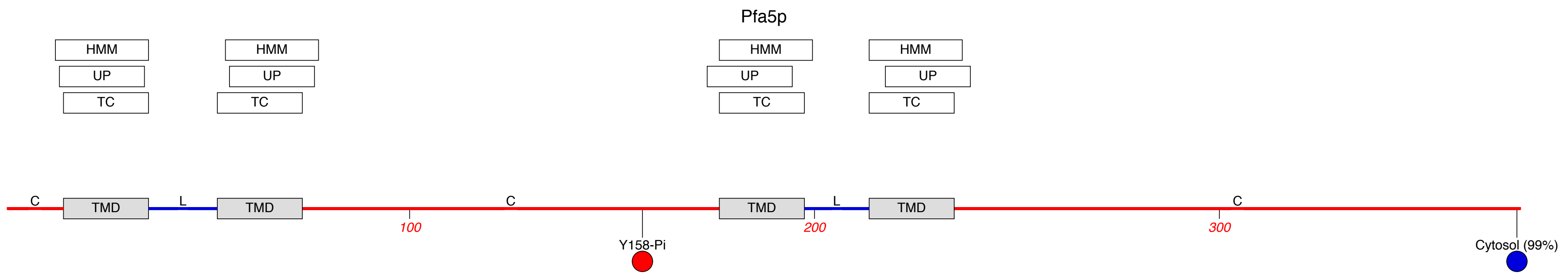
Pfa5p
HMM
UP
TC
HMM
UP
TC
HMM
UP
TC
HMM
UP
TC
C
TMD
L
TMD
C
TMD
L
TMD
C
100
200
300
Y158-Pi
Cytosol (99%)

Phs1p

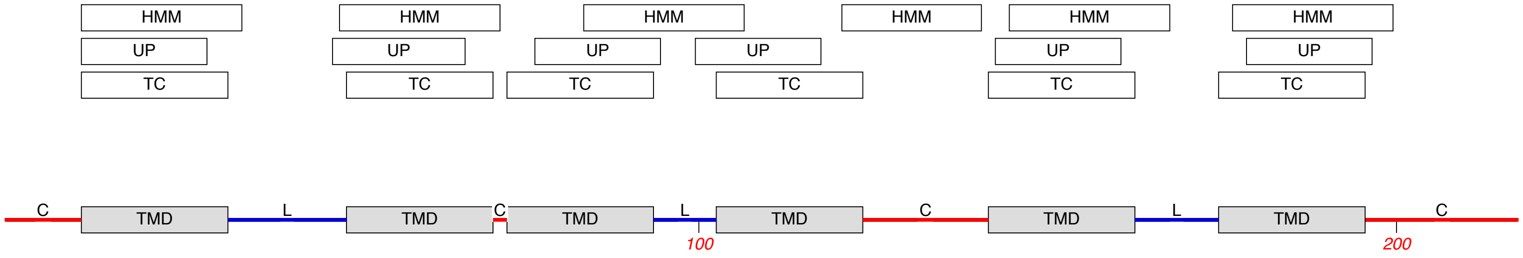
HMM
HMM
HMM
HMM
HMM
HMM
UP
UP
UP
UP
UP
UP
TC
TC
TC
TC
TC
TC
C
TMD
L
TMD
C
TMD
L
TMD
C
TMD
L
TMD
C
100
200

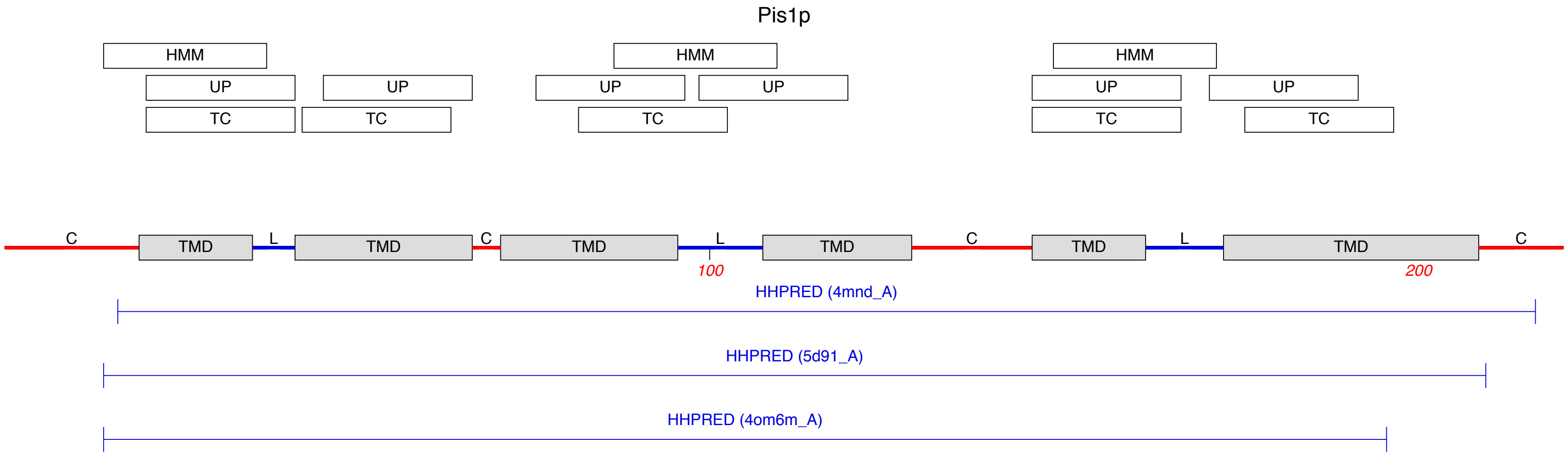
Pis1p
HMM
UP
UP
TC
TC
HMM
UP
UP
TC
HMM
UP
UP
TC
TC
C
TMD
L
TMD
C
TMD
L
TMD
C
TMD
L
TMD
C
100
200
HHPRED (4mnd_A)
HHPRED (5d91_A)
HHPRED (4om6m_A)

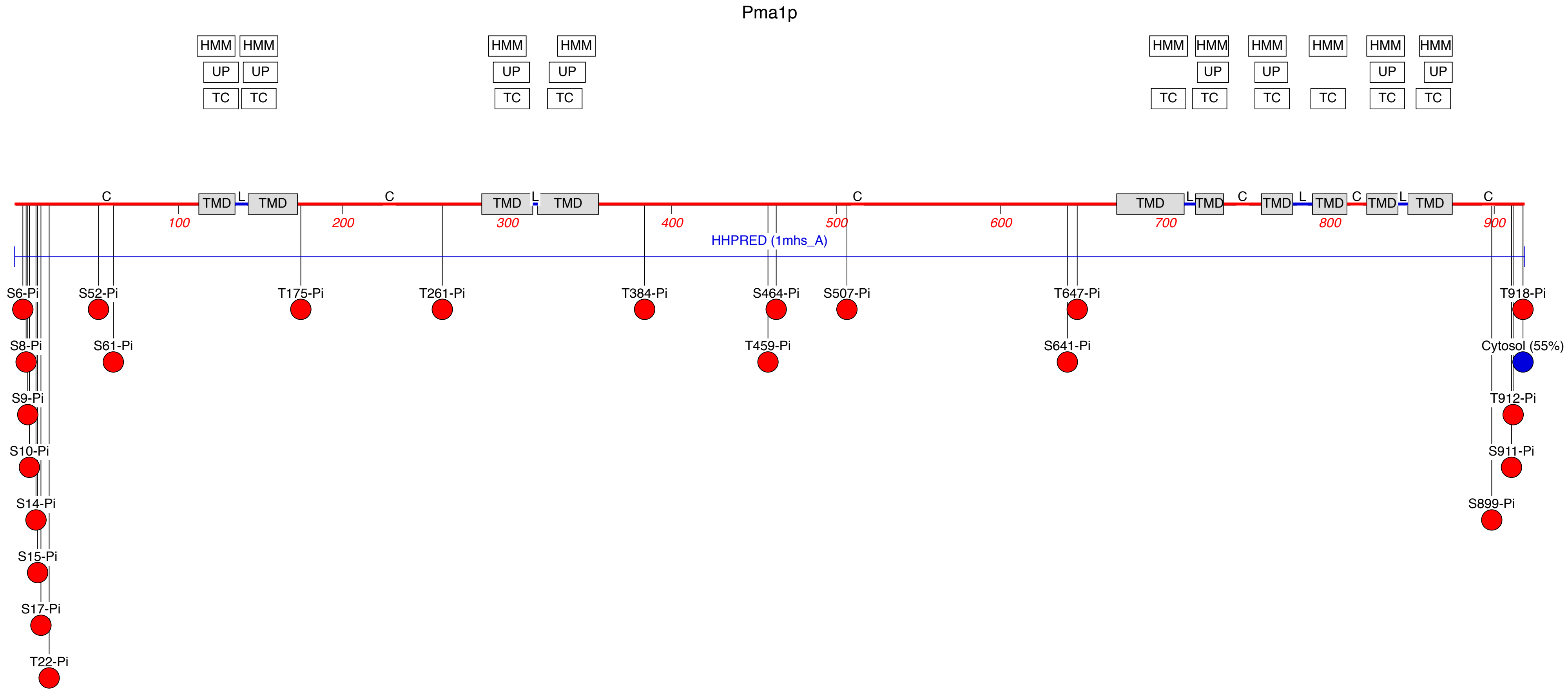

Pma1p
HMM
UP
TC
TMD
L
C
100
200
300
400
500
600
700
800
900
HHPRED (1mhs_A)
S6-Pi
S8-Pi
S9-Pi
S10-Pi
S14-Pi
S15-Pi
S17-Pi
T22-Pi
S52-Pi
S61-Pi
T175-Pi
T261-Pi
T384-Pi
S464-Pi
T459-Pi
S507-Pi
T647-Pi
S641-Pi
T918-Pi
Cytosol (55%)
T912-Pi
S911-Pi
S899-Pi

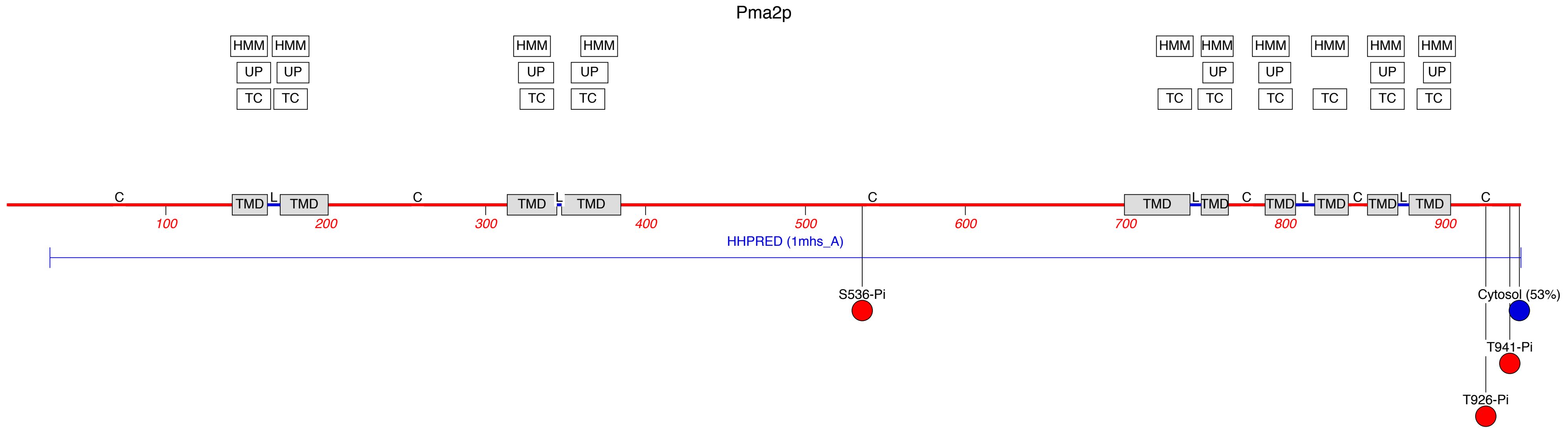

Pma2p
HMM
UP
TC
TMD
L
C
100
200
300
400
500
600
700
800
900
HHPRED (1mhs_A)
S536-Pi
Cytosol (53%)
T941-Pi
T926-Pi

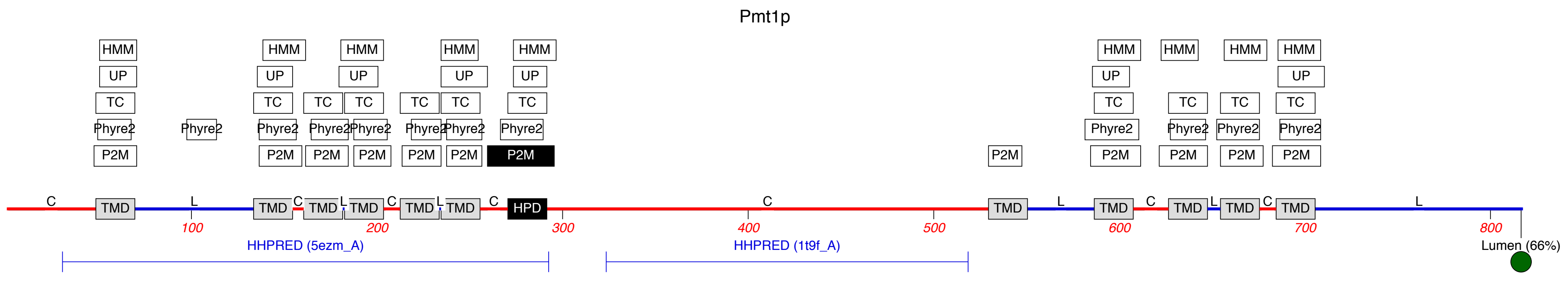
Pmt1p
HMM
UP
TC
Phyre2
P2M
TMD
HPD
C
L
100
200
300
400
500
600
700
800
HHPRED (5ezm_A)
HHPRED (1t9f_A)
Lumen (66%)

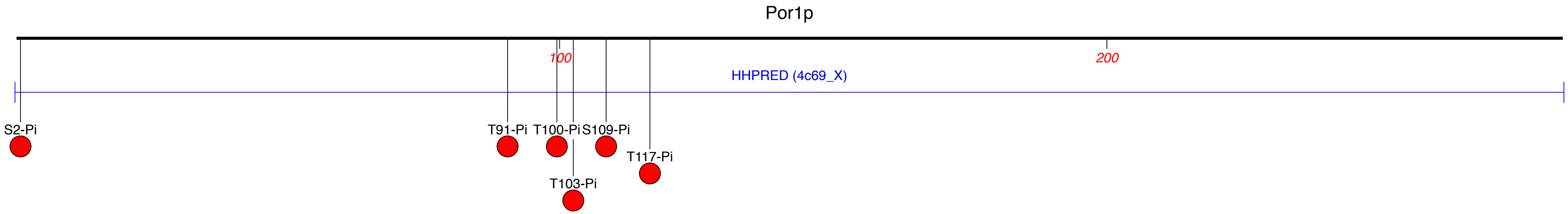
Por1p
100
200
HHPRED (4c69_X)
S2-Pi
T91-Pi
T100-Pi
S109-Pi
T117-Pi
T103-Pi

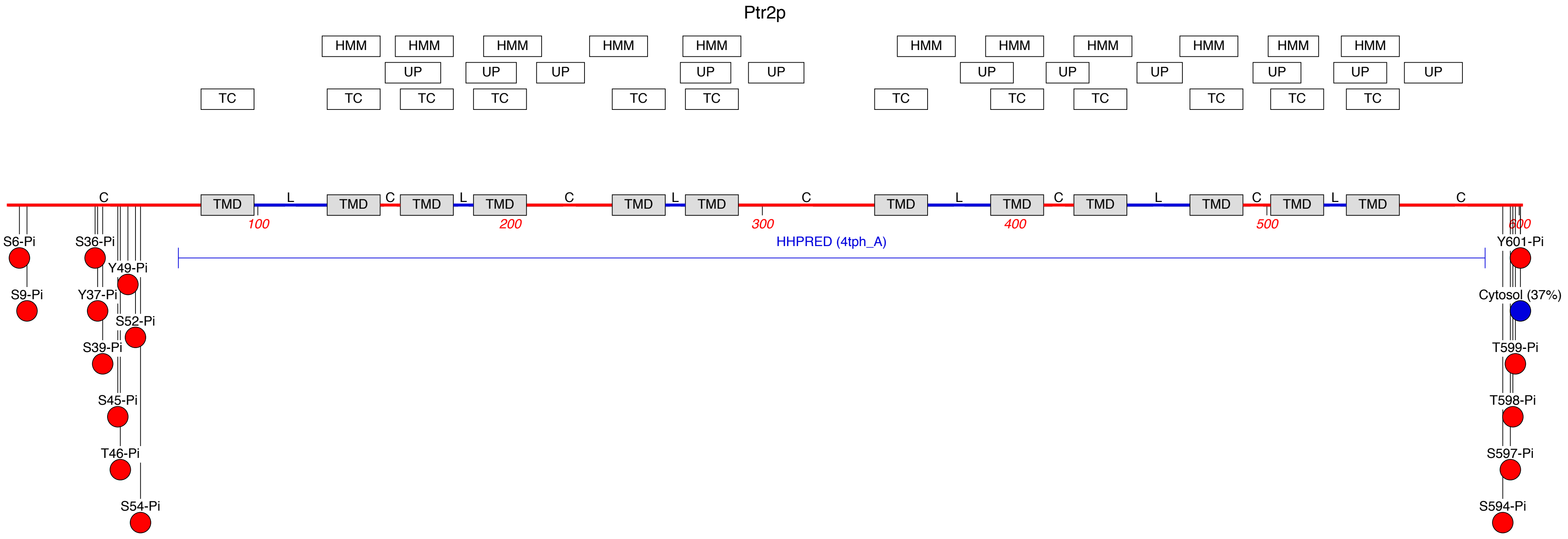

Ptr2p
HMM
UP
TC
TMD
L
C
100
200
300
400
500
600
HHPRED (4tph_A)
S6-Pi
S9-Pi
S36-Pi
Y37-Pi
S39-Pi
S45-Pi
T46-Pi
Y49-Pi
S52-Pi
S54-Pi
Y601-Pi
Cytosol (37%)
T599-Pi
T598-Pi
S597-Pi
S594-Pi

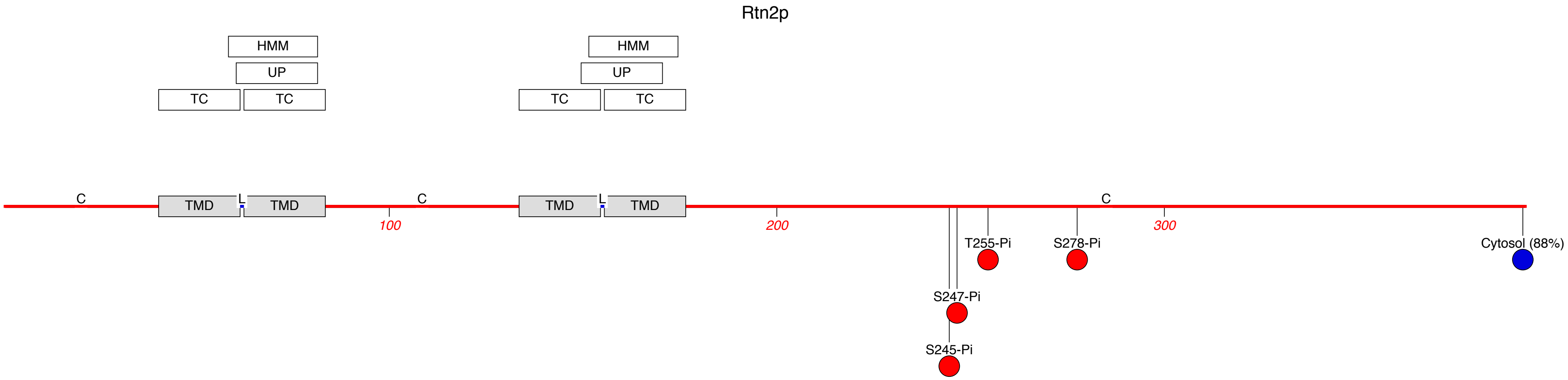

Rtn2p
HMM
UP
TC
TC
HMM
UP
TC
TC
C
TMD
L
TMD
C
TMD
L
TMD
C
100
200
300
T255-Pi
S278-Pi
Cytosol (88%)
S247-Pi
S245-Pi

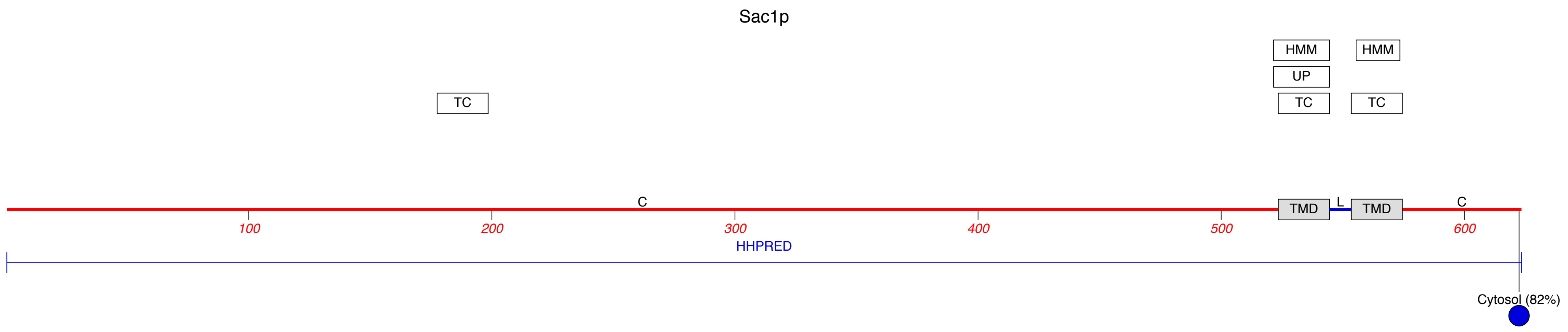

Sac1p
HMM
HMM
UP
TC
TC
TC
C
TMD
L
TMD
C
100
200
300
400
500
600
HHPRED
Cytosol (82%)

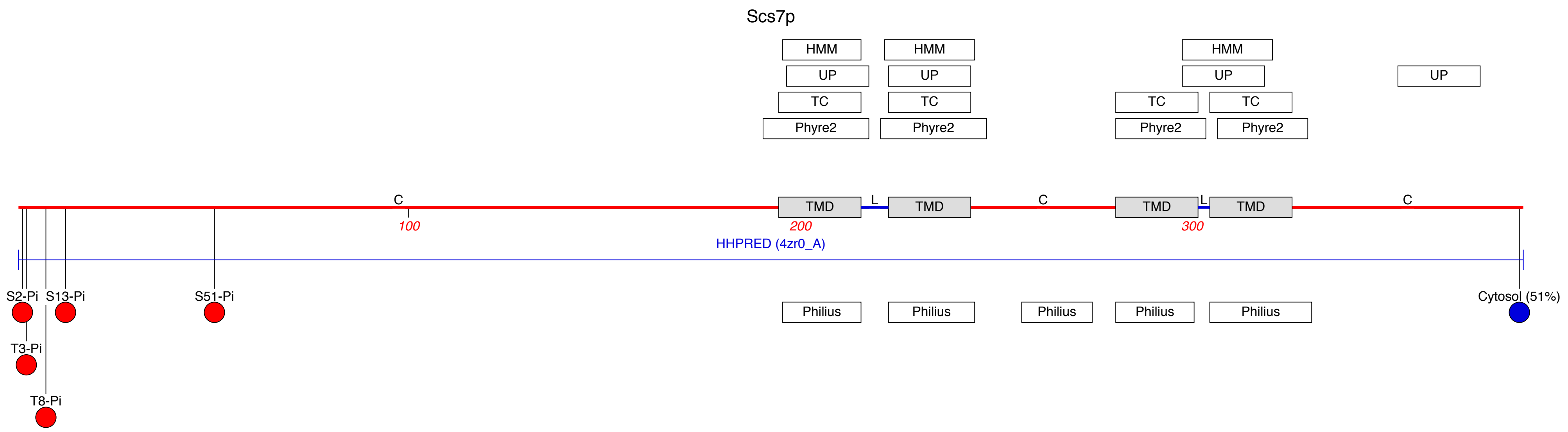

Scs7p
HMM
HMM
HMM
UP
UP
UP
UP
TC
TC
TC
TC
Phyre2
Phyre2
Phyre2
Phyre2
C
L
C
L
C
TMD
TMD
TMD
TMD
100
200
300
HHPRED (4zr0_A)
S2-Pi
S13-Pi
S51-Pi
T3-Pi
T8-Pi
Philius
Philius
Philius
Philius
Philius
Cytosol (51%)

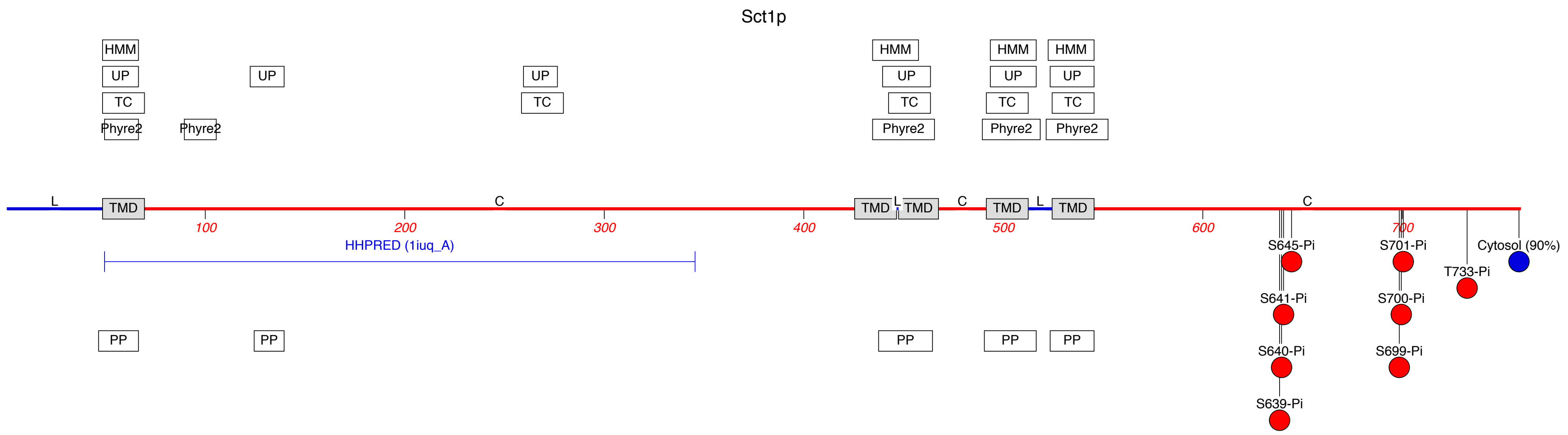
Sct1p
HMM
UP
TC
Phyre2
L
TMD
C
100
200
300
400
500
600
700
HHPRED (1iuq_A)
PP
S645-Pi
S641-Pi
S640-Pi
S639-Pi
S701-Pi
S700-Pi
S699-Pi
T733-Pi
Cytosol (90%)

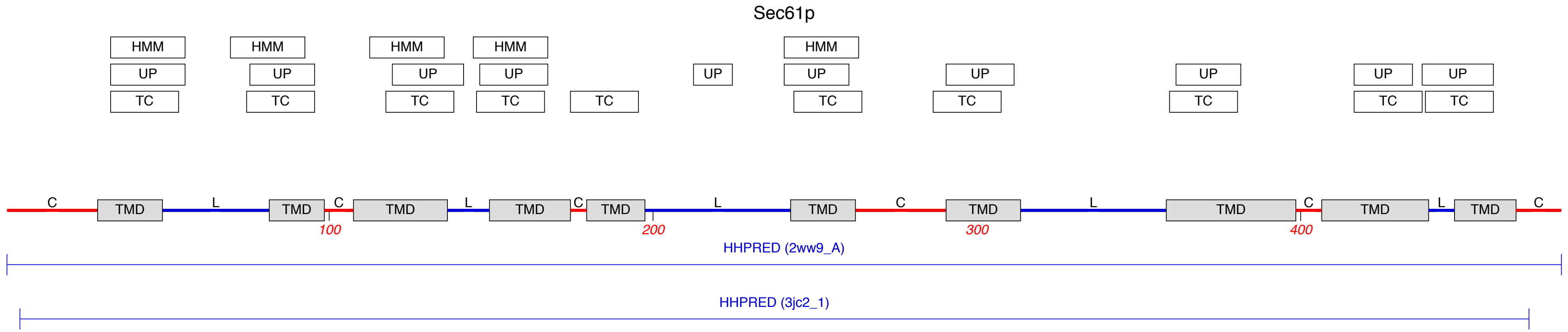
Sec61p
HMM
HMM
HMM
HMM
HMM
UP
UP
UP
UP
UP
UP
UP
UP
UP
UP
TC
TC
TC
TC
TC
TC
TC
TC
TC
TC
C
TMD
L
TMD
C
TMD
L
TMD
C
TMD
L
TMD
C
TMD
L
TMD
C
TMD
L
TMD
C
100
200
300
400
HHPRED (2ww9_A)
HHPRED (3jc2_1)

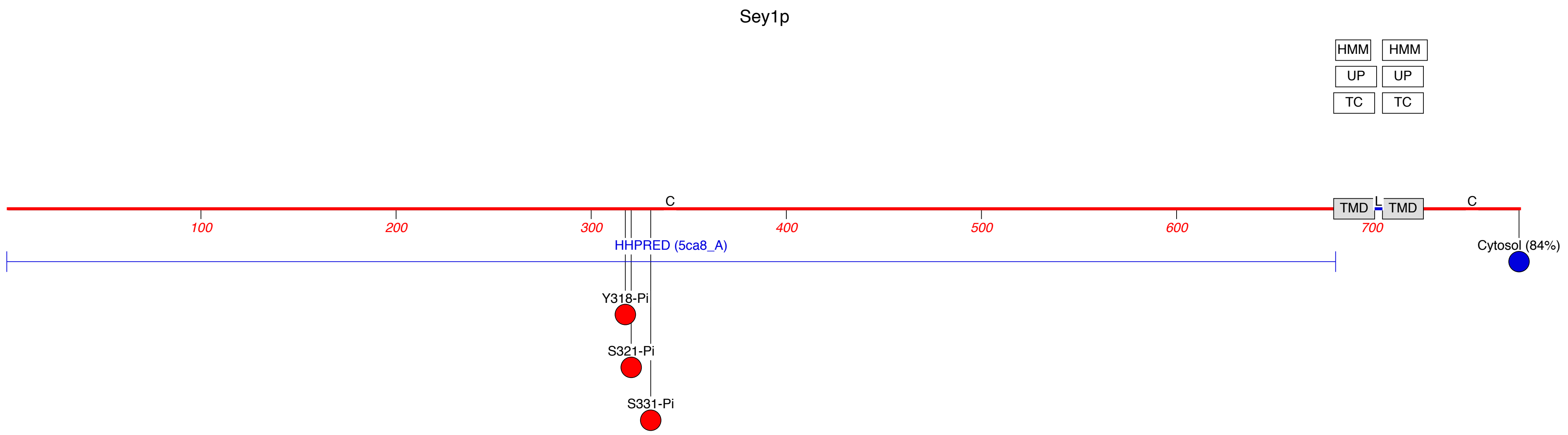

Sey1p
HMM
HMM
UP
UP
TC
TC
C
TMD
L
TMD
C
100
200
300
400
500
600
700
HHPRED (5ca8_A)
Cytosol (84%)
Y318-Pi
S321-Pi
S331-Pi

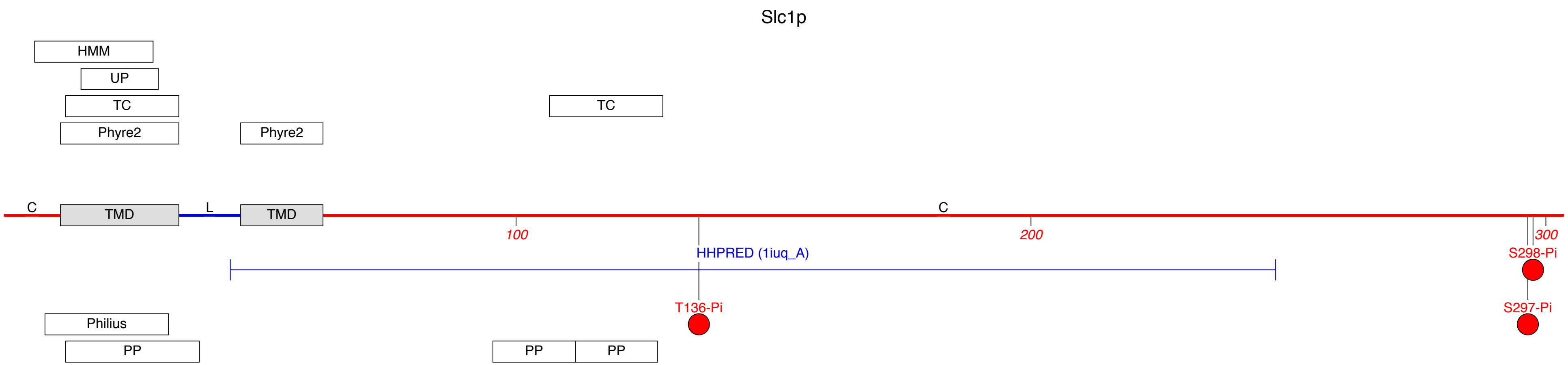
Slc1p
HMM
UP
TC
TC
Phyre2
Phyre2
C
TMD
L
TMD
C
100
200
300
HHPRED (1iuq_A)
S298-Pi
T136-Pi
S297-Pi
Philius
PP
PP
PP

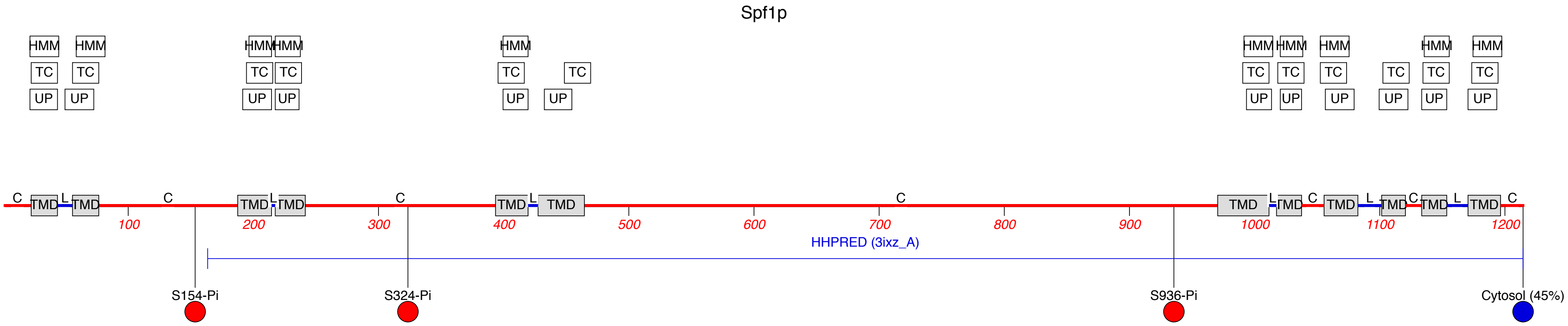

Spf1p
HMM
TC
UP
TMD
C
L
100
200
300
400
500
600
700
800
900
1000
1100
1200
HHPRED (3ixz_A)
S154-Pi
S324-Pi
S936-Pi
Cytosol (45%)

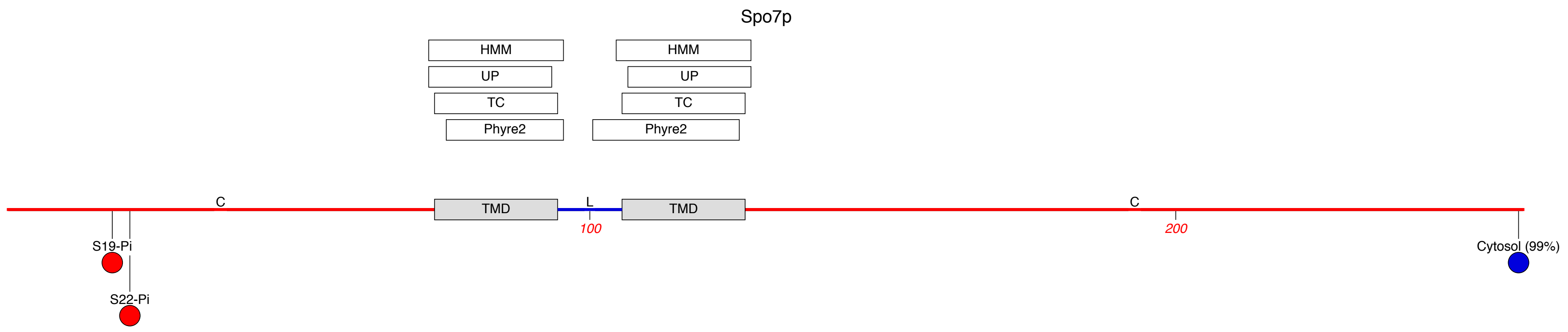
Spo7p
HMM
UP
TC
Phyre2
HMM
UP
TC
Phyre2
C
TMD
L
TMD
C
100
200
S19-Pi
S22-Pi
Cytosol (99%)

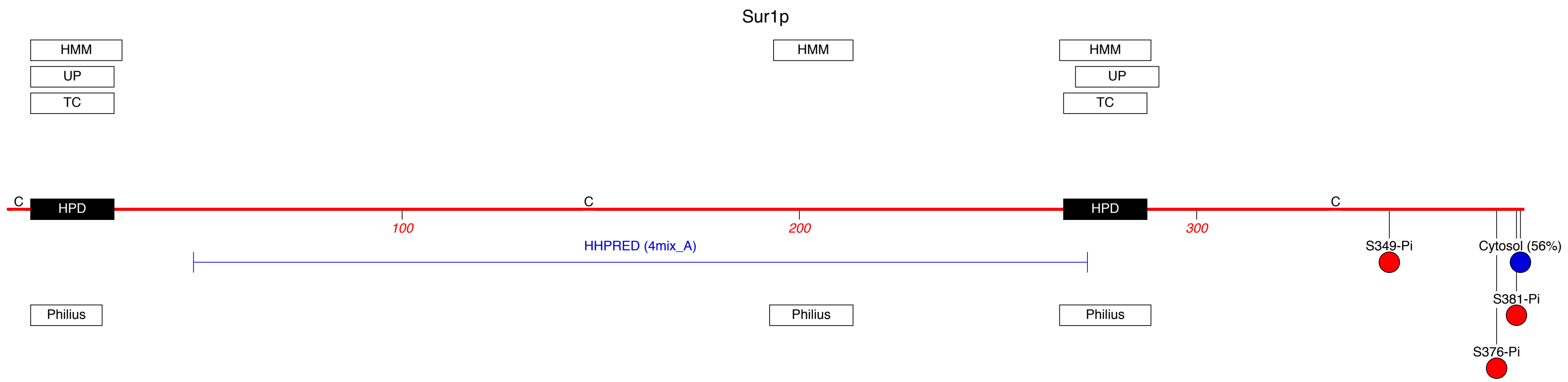
Sur1p
HMM
UP
TC
HMM
HMM
UP
TC
C
HPD
C
HPD
C
100
200
300
HHPRED (4mix_A)
S349-Pi
Cytosol (56%)
S381-Pi
S376-Pi
Philius
Philius
Philius

Sur2p

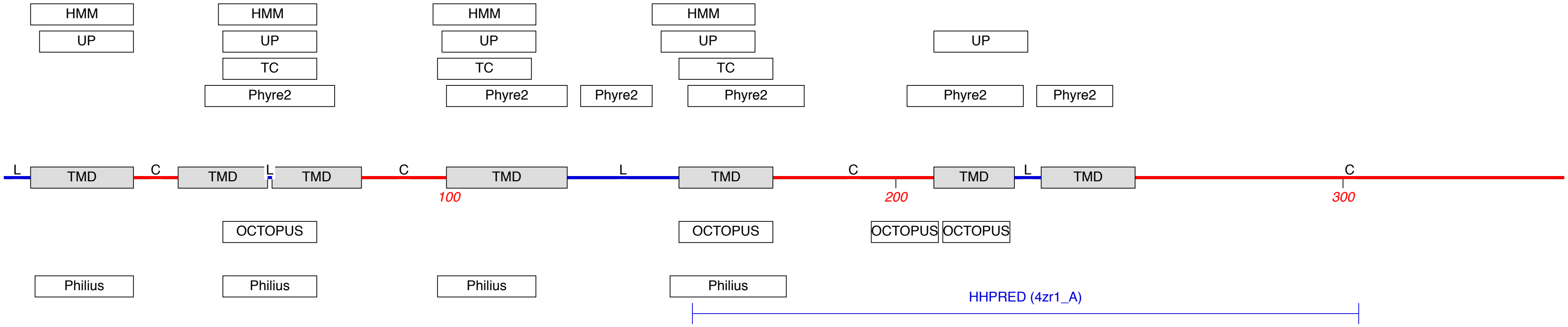
HMM
UP
TC
Phyre2
TMD
L
C
100
200
300
OCTOPUS
Philius
HHPRED (4zr1_A)

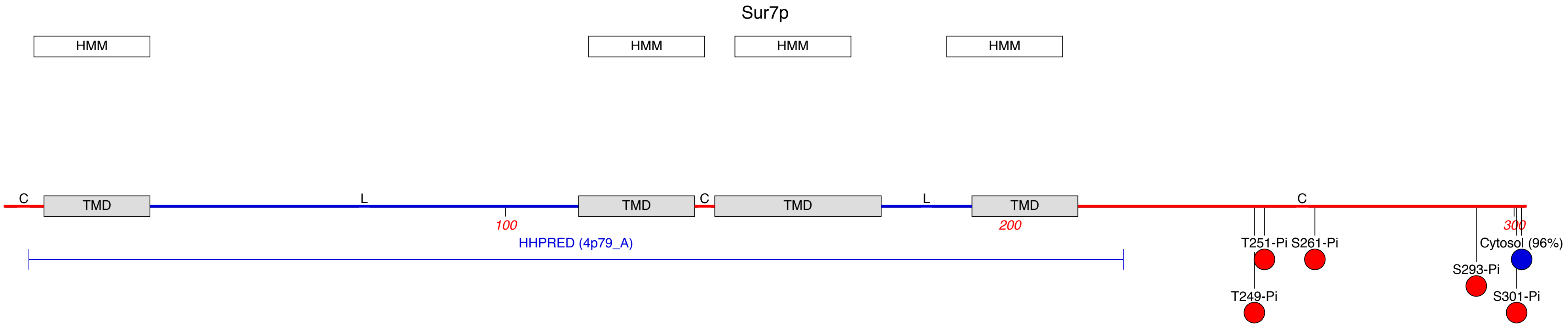
Sur7p
HMM
HMM
HMM
HMM
C
TMD
L
TMD
C
TMD
L
TMD
C
100
200
300
HHPRED (4p79_A)
T251-Pi
S261-Pi
Cytosol (96%)
S293-Pi
T249-Pi
S301-Pi

Swf1p

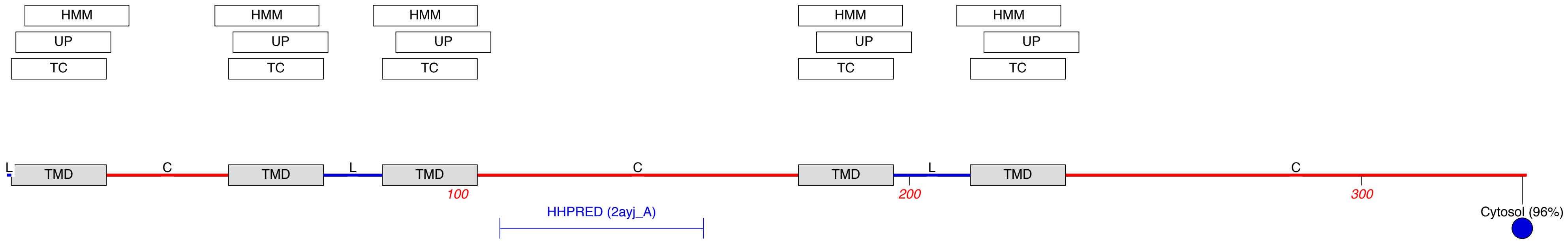

Taz1p

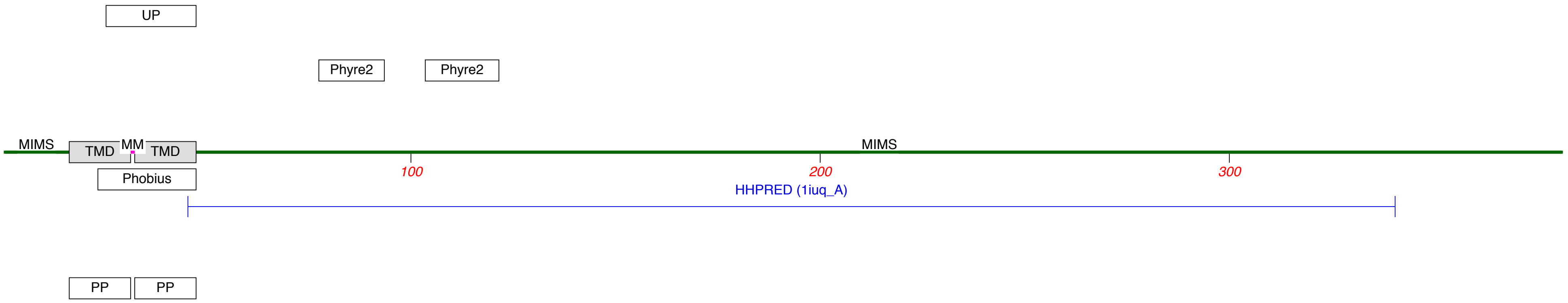
UP
Phyre2
Phyre2
MIMS
TMD
MM
TMD
MIMS
Phobius
100
200
300
HHPRED (1iuq_A)
PP
PP

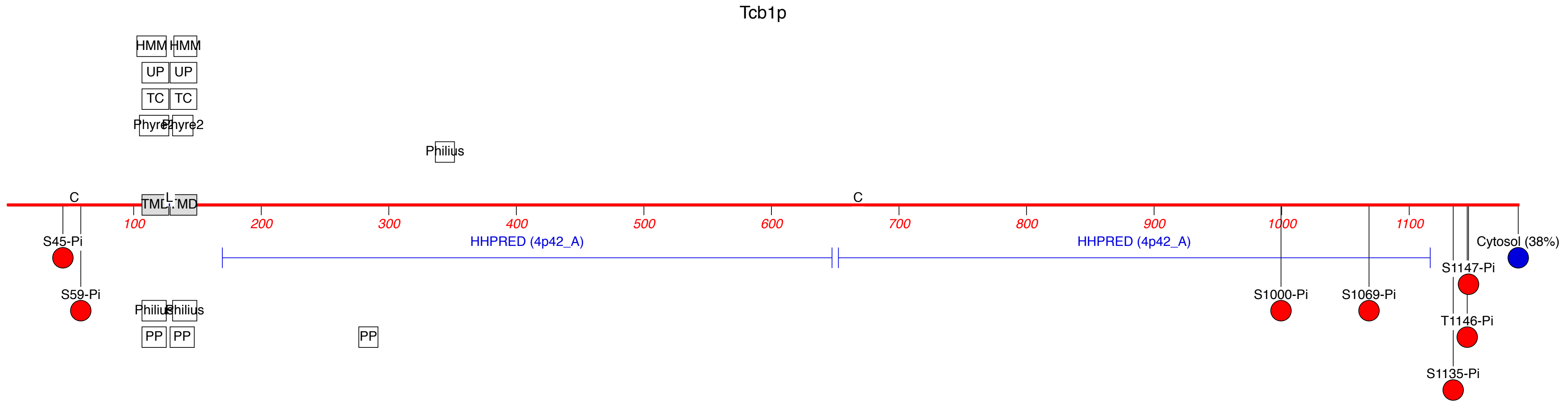
Tcb1p
HMM
HMM
UP
UP
TC
TC
Phyre2
Phyre2
Philius
C
TMD
L
TMD
C
100
200
300
400
500
600
700
800
900
1000
1100
S45-Pi
S59-Pi
HHPRED (4p42_A)
HHPRED (4p42_A)
Cytosol (38%)
S1147-Pi
S1000-Pi
S1069-Pi
T1146-Pi
S1135-Pi
Philius
Philius
PP
PP
PP

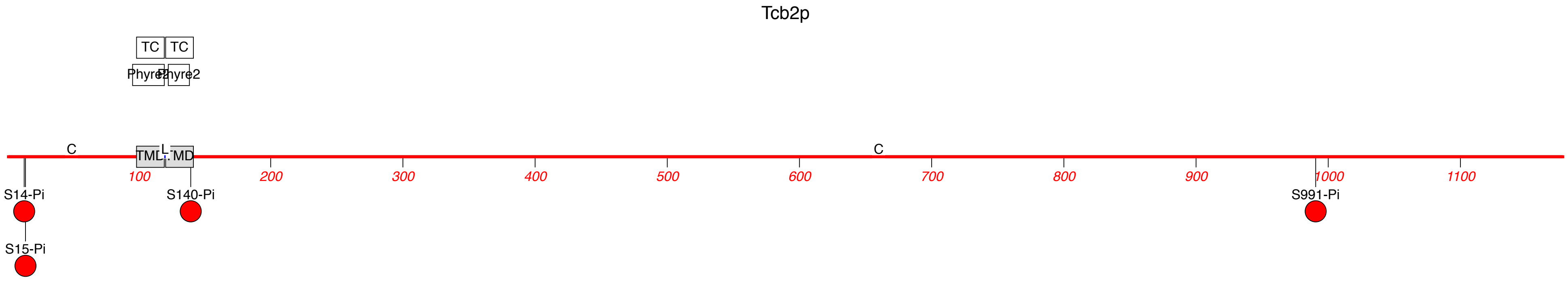
Tcb2p
TC
TC
Phyre2
Phyre2
C
TMD
L
TMD
C
100
200
300
400
500
600
700
800
900
1000
1100
S14-Pi
S15-Pi
S140-Pi
S991-Pi

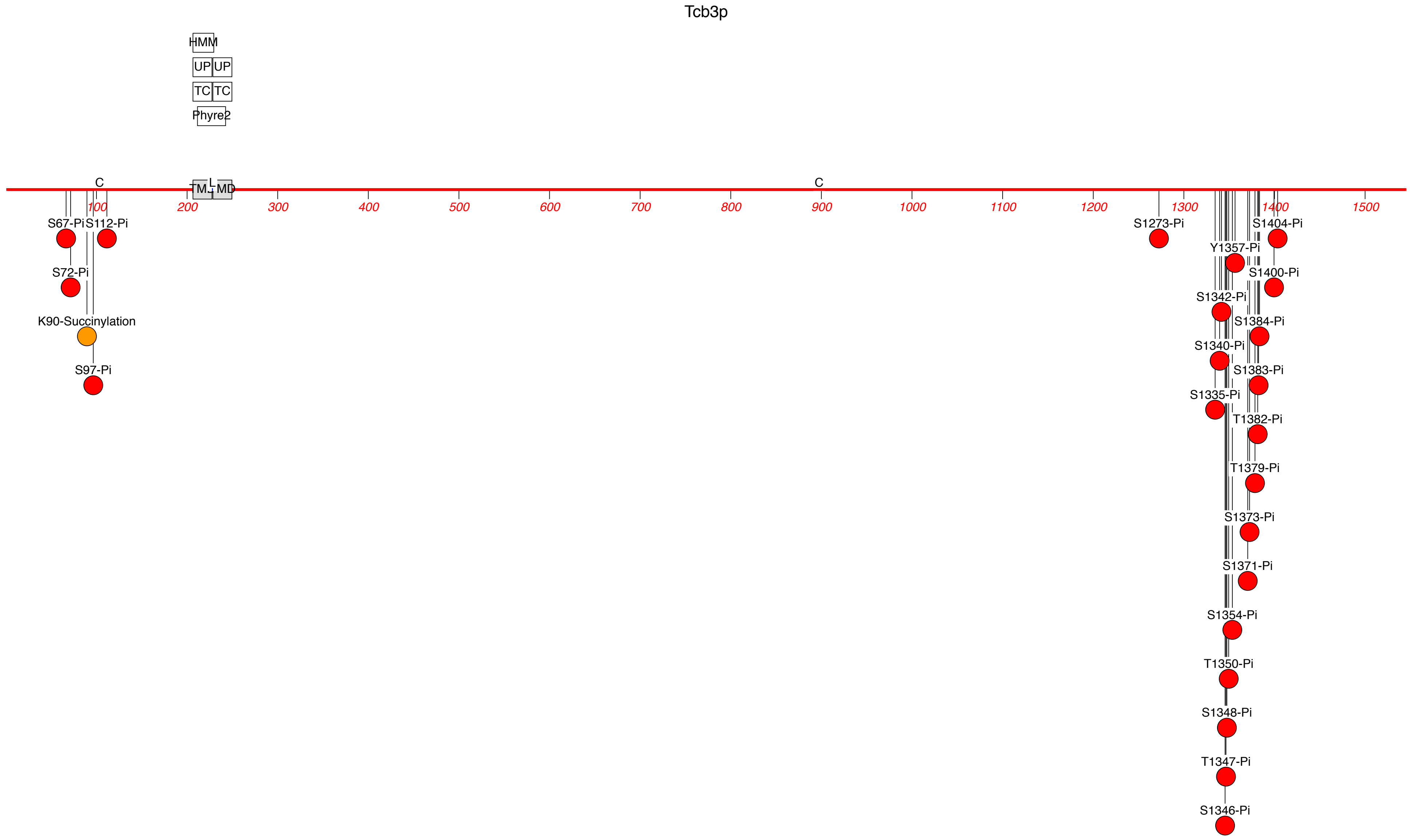
Tcb3p
HMM
UP
UP
TC
TC
Phyre2
C
TM
L
MD
C
100
200
300
400
500
600
700
800
900
1000
1100
1200
1300
1400
1500
S67-Pi
S112-Pi
S72-Pi
K90-Succinylation
S97-Pi
S1273-Pi
S1404-Pi
Y1357-Pi
S1400-Pi
S1342-Pi
S1384-Pi
S1340-Pi
S1383-Pi
S1335-Pi
T1382-Pi
T1379-Pi
S1373-Pi
S1371-Pi
S1354-Pi
T1350-Pi
S1348-Pi
T1347-Pi
S1346-Pi

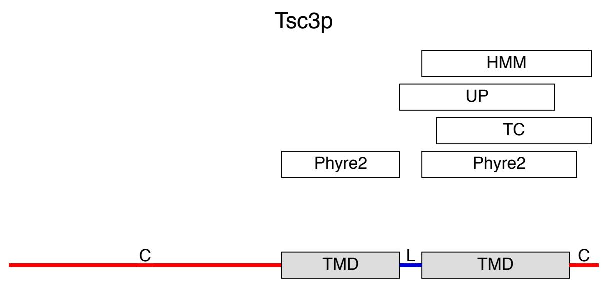
Tsc3p
HMM
UP
TC
Phyre2
Phyre2
C
TMD
L
TMD
C

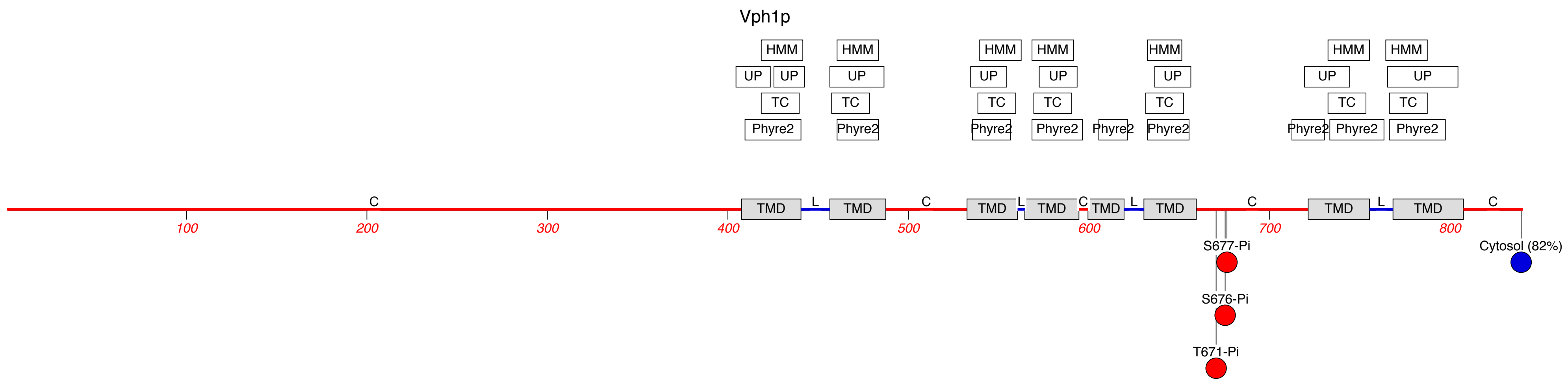
Vph1p
HMM
UP
TC
Phyre2
TMD
L
C
100
200
300
400
500
600
700
800
S677-Pi
S676-Pi
T671-Pi
Cytosol (82%)

Wbp1p

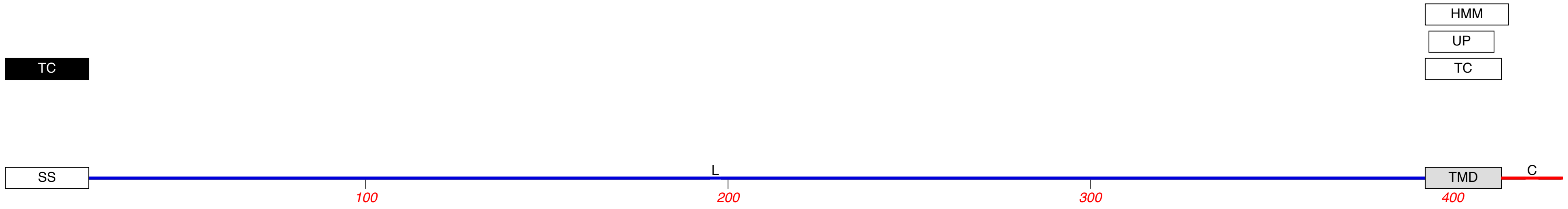

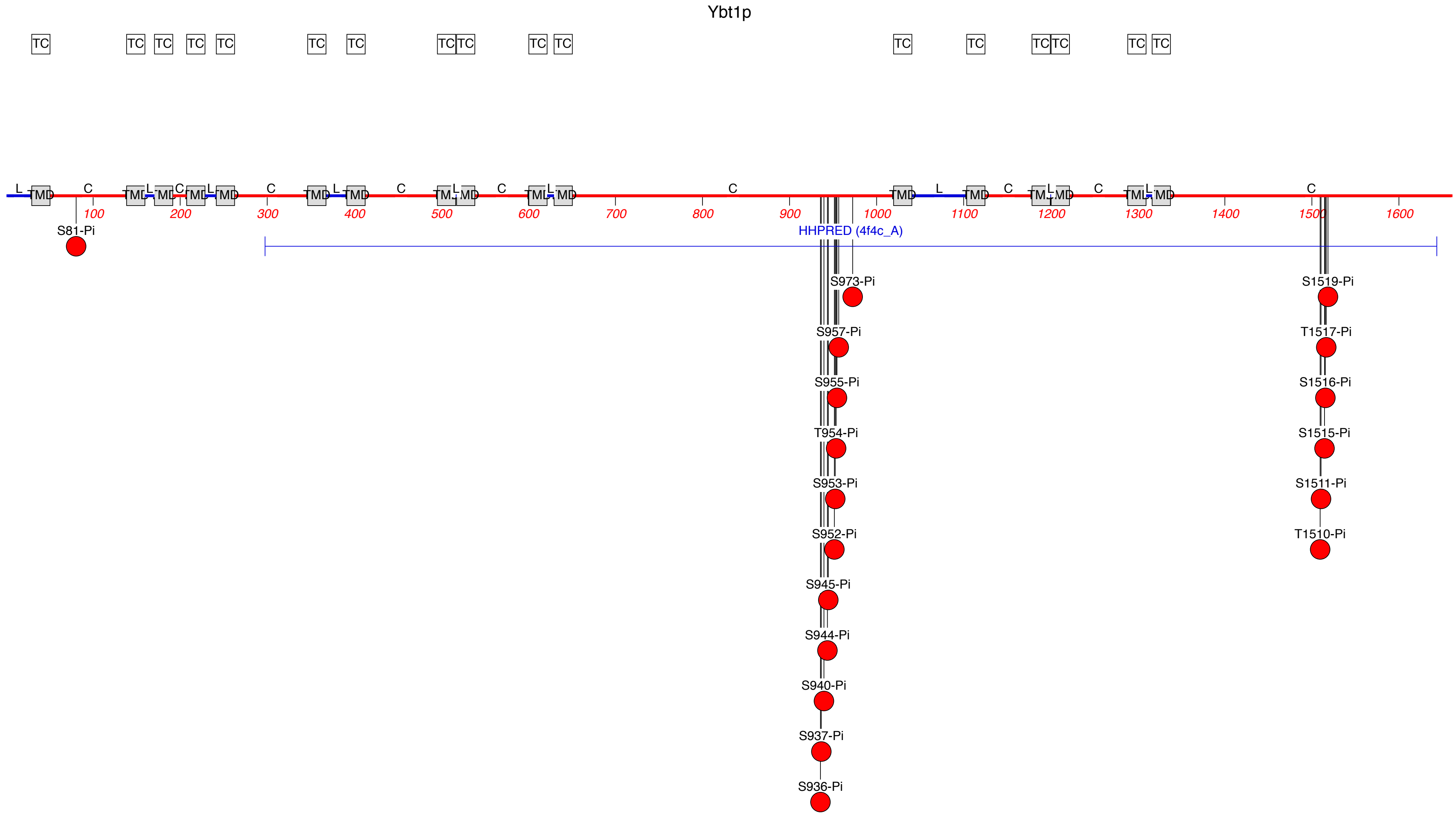
Ybt1p
TC TC TC TC TC TC TC TC TC TC TC TC TC TC TC TC TC
L TMD C TMD L TMD C TMD L TMD C TMD L TMD C TMD L TMD C TMD L TMD C TMD L TMD C TMD L TMD C TMD L TMD C
100 200 300 400 500 600 700 800 900 1000 1100 1200 1300 1400 1500 1600
S81-Pi
HHPRED (4f4c_A)
S973-Pi
S957-Pi
S955-Pi
T954-Pi
S953-Pi
S952-Pi
S945-Pi
S944-Pi
S940-Pi
S937-Pi
S936-Pi
S1519-Pi
T1517-Pi
S1516-Pi
S1515-Pi
S1511-Pi
T1510-Pi

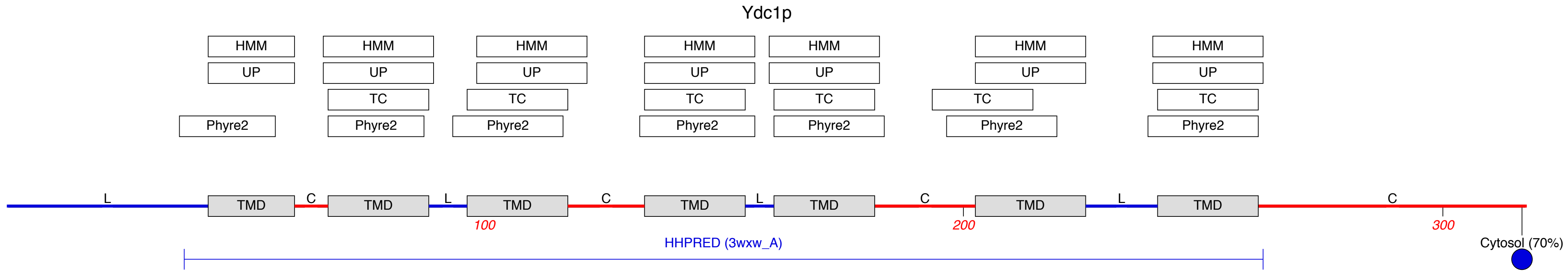
Ydc1p
HMM
UP
TC
Phyre2
L
C
TMD
100
200
300
HHPRED (3wxw_A)
Cytosol (70%)

YDR018C

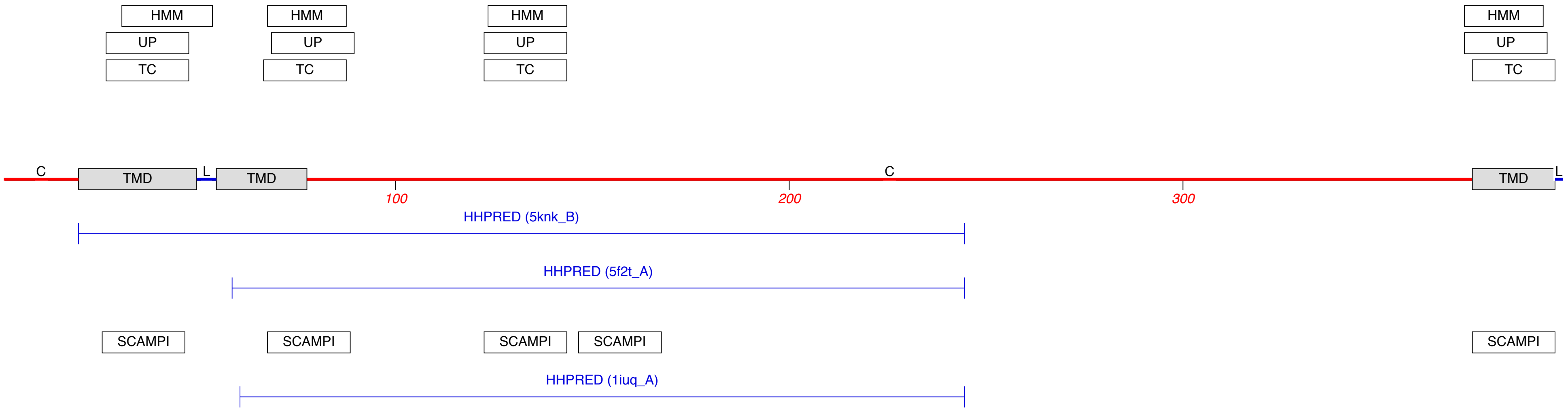
HMM
UP
TC
HMM
UP
TC
HMM
UP
TC
HMM
UP
TC
C
TMD
L
TMD
C
TMD
L
100
200
300
HHPRED (5knk_B)
HHPRED (5f2t_A)
SCAMPI
SCAMPI
SCAMPI
SCAMPI
SCAMPI
HHPRED (1iuq_A)

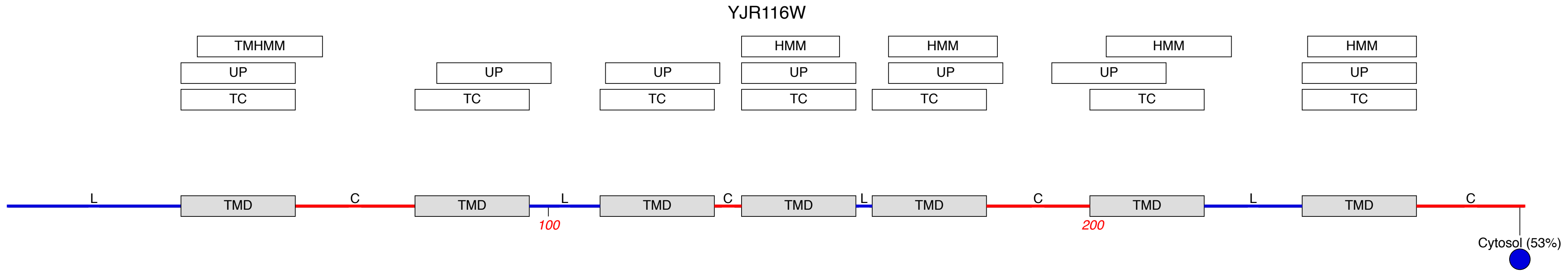
YJR116W
TMHMM
HMM
HMM
HMM
HMM
UP
UP
UP
UP
UP
UP
UP
TC
TC
TC
TC
TC
TC
TC
L
TMD
C
TMD
L
TMD
C
TMD
L
TMD
C
TMD
L
TMD
C
100
200
Cytosol (53%)

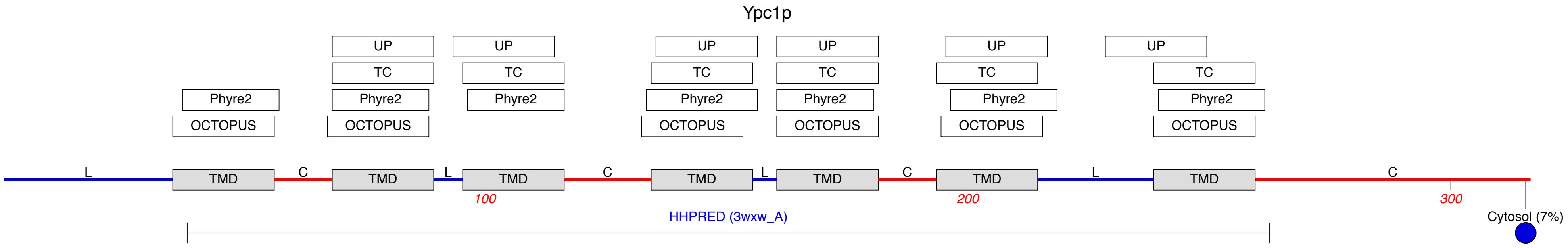
Ypc1p
UP
TC
Phyre2
OCTOPUS
TMD
L
C
100
200
300
HHPRED (3wxw_A)
Cytosol (7%)

YPR114W

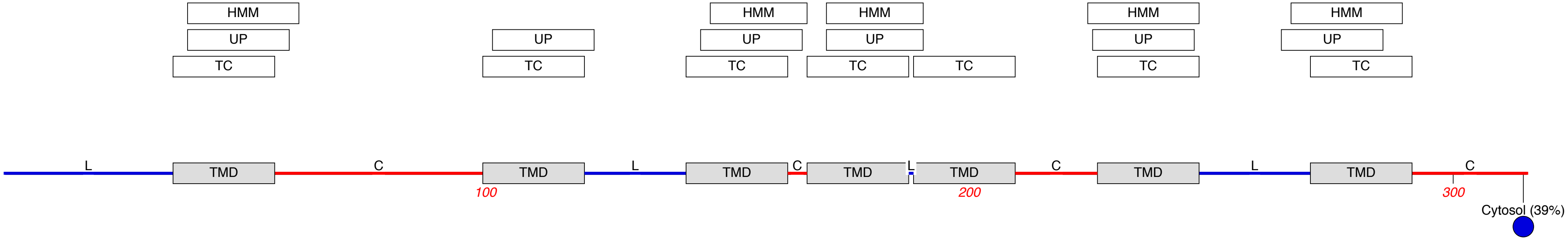

Ysr3p

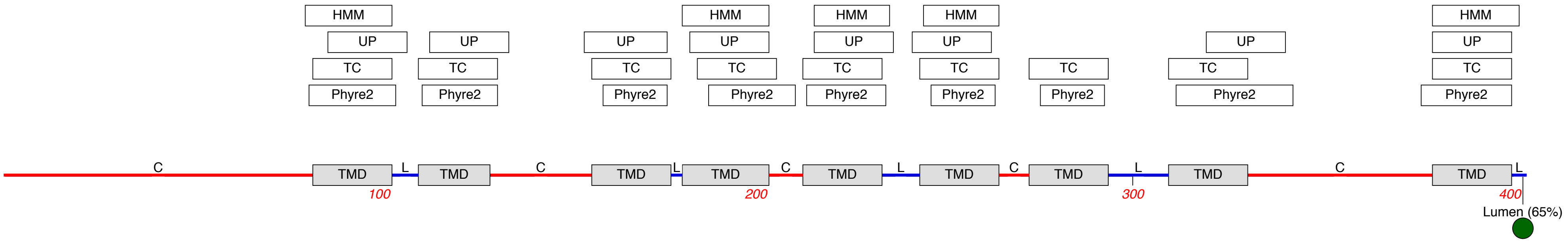

Supplement: S3 Fig — (PDF) [file pone.0186840.s018.pdf]
